# Comparative Analyses of Complete Chloroplast Genomes of *Microula sikkimensis* and Related Species of Boraginaceae

**DOI:** 10.3390/genes15020226

**Published:** 2024-02-10

**Authors:** Yunqing Gao, Zhenjiang Chen, Xiuzhang Li, Kamran Malik, Chunjie Li

**Affiliations:** 1 State Key Laboratory of Herbage Improvement and Grassland Agro-Ecosystems, Lanzhou University, Lanzhou 730000, China; 220220902301@lzu.edu.cn (Y.G.); malik@lzu.edu.cn (K.M.); 2 Key Laboratory of Grassland Livestock Industry Innovation, Ministry of Agriculture and Rural Affairs, Lanzhou University, Lanzhou 730000, China; 3 College of Pastoral Agriculture Science and Technology, Lanzhou University, Lanzhou 730000, China; 4 Engineering Research Center of Grassland Industry, Ministry of Education, Lanzhou University, Lanzhou 730000, China; 5 Gansu Tech Innovation Centre of Western China Grassland Industry, Lanzhou University, Lanzhou 730000, China; 6 Centre for Grassland Microbiome, Lanzhou University, Lanzhou 730000, China; 7 Qinghai Academy of Animal and Veterinary Science, Qinghai University, Xining 810016, China; xiuzhang11@163.com

**Keywords:** *Microula sikkimensis*, chloroplast genomes, comparative analysis, phylogenetic analysis, Boraginaceae

## Abstract

The present study provides a detailed analysis of the chloroplast genome of *Microula sikkimensis*. The genome consisted of a total of 149,428 bp and four distinct regions, including a large single-copy region (81,329 bp), a small single-copy region (17,261 bp), and an inverted repeat region (25,419 bp). The genome contained 112 genes, including 78 protein-coding genes, 30 tRNA genes, and 4 rRNA genes, and some exhibited duplication in the inverted repeat region. The chloroplast genome displayed different GC content across regions, with the inverted repeat region exhibiting the highest. Codon usage analysis and the identification of simple sequence repeats (SSRs) offer valuable genetic markers. Comparative analysis with other Boraginaceae species highlighted conservation and diversity in coding and noncoding regions. Phylogenetic analysis placed *M. sikkimensis* within the Boraginaceae family, revealing its distinct relationship with specific species.

## 1. Introduction

*Microula sikkimensis*, a biennial herbaceous plant from the Boraginaceae family, thrives in the high-altitude grasslands, forests, shrublands, and secondary vegetation at elevations ranging from 2500 to 4000 m on the eastern edge of the Qinghai-Tibet Plateau [1]. *M. sikkimensis* is a plant resource rich in γ-linolenic acid, with promising development prospects. Abundant experiments have confirmed that *M. sikkimensis* oil significantly reduces the levels of total cholesterol (TC), triglycerides (TG), and serum malondialdehyde (MDA) in the liver and serum. It also increases the ratio of high-density lipoprotein cholesterol to total cholesterol (HDL-C/TC) in the serum. Moreover, it effectively reduces the deposition of cholesterol in peripheral tissue cells, thus preventing atherosclerosis and maintaining the integrity of the biomembrane structure. Its capacity to lower triglycerides in the liver and serum surpasses that of atorvastatin. *M. sikkimensis* oil can improve the high blood lipidemia by reducing blood viscosity, preventing thrombosis, and exhibiting unique solvent properties [1,2,3]. The harvested stalks of *M. sikkimensis* exhibit heightened palatability and are nutritionally dense, making them a crucial coarse fodder for supplementing livestock in the winter and spring seasons in high-altitude pastoral regions. The research of *M. sikkimensis* oil will provide enough supply of raw materials for the production of a series of high-nutrition health foods, fortified dairy products, healthful edible oils, as well as specialized pharmaceuticals and novel cosmetics [4].

Chloroplasts are specialized energy converters unique to higher plants and certain algae. These organelles are essential for their role in carrying autonomous genetic information, necessary for cellular functions [5,6,7]. The plant chloroplast genome is generally a double-stranded circular molecule composed of four main regions: a large single copy (LSC) region, a small single copy (SSC) region, and two inverted repeat (IRA and IRB) regions, which are identical in sequence but opposite in orientation [8,9,10,11]. The relatively small (115–165 kb) and conservative nature of the chloroplast genome makes it a crucial tool for the exploration of genomic evolution and the analysis of phylogenetic relationships within angiosperms [12,13,14]. Additionally, the chloroplast genome finds widespread applications in diverse domains, including population genetics, molecular-assisted breeding, gene mapping, plant barcode sequence screening and gene diversity studies [15,16,17,18,19].

Currently, research findings on the chloroplast genome of *M. sikkimensis* remain unpublished. In this research, we presented the first report and analysis of the complete chloroplast genome sequence of *M. sikkimensis* by acquiring insights into the fundamental genome structure, simple sequence repeats (SSRs), and codon usage bias. Additionally, comparative genomic analysis and phylogenetic analysis of *M. sikkimensis* chloroplast genome was conducted in relation to other species within the Boraginaceae family. These research findings not only fill the existing gap in scientific research regarding the chloroplast genome of *M. sikkimensis* but also lay a foundational stone for future genetic and evolutionary studies.

## 2. Materials and Methods

### 2.1. Sample Collection and DNA Extraction

The *M. sikkimensis* plants were collected from Zaduo county, Qinghai province, China. The fresh leaves were frozen in liquid nitrogen and stored at −80 °C. The cetyltrimethylammonium bromide (CTAB) method was used to extract total genomic DNA. The quality of DNA was measured by NanoDrop 2000 (Thermo Scientific, Wilmington, NC, USA) and agarose gel electrophoresis.

### 2.2. Genome Sequencing, Assembly and Annotation

Library construction was carried out by NexteraXT DNA Library Preparation Kit (Illumina, Shanghai, China), with 300 bp-short-insert fragments. Library quality was assessed using GeneRead DNA QuantiMIZE Assay Kit (Qiagen, Duesseldorf, Germany). Sequencing of qualified library was conducted utilizing Illumina NovaSeq 6000 (Wuhan Benagen Tech Solutions Co., Ltd., Wuhan, China). Initial raw sequencing output was subjected to quality control using SOAPnuke (Version: 2.1.0), with filtering parameters set to remove reads with more than 5% N base content, reads where the number of low-quality bases (quality score less than or equal to 5) reaches 50%, reads contaminated with adapters and duplicate sequences caused by PCR amplification. GetOrganelle software (version 1.7.7.0) [20] was utilized for assembling the chloroplast genome with the default settings. CPGAVAS software (v2) [21] was used to annotate the chloroplast genome while OGDRAW software (v1.1.1) [22] was used to visualize the circular chloroplast genome map of *M. sikkimensis*. The tRNA of the chloroplast genome was annotated using tRNAscanSE software (v.2.0.11) [23]. The rRNA of the chloroplast genome was annotated using BLASTN software (v2.13.0) [24]. The annotation errors of each chloroplast genome were manually corrected using online tool CPGView [25] and Apollo software (v1.11.8) [26]. The fully annotated chloroplast genome was subsequently submitted to the GenBank database (Accession Number: OR866440). 

### 2.3. Comparative Genome Analysis

The complete chloroplast genomes of *M. sikkimensis* and other four species were compared using the MVISTA program [27] with the shuffle-LAGAN model, with *M. sikkimensis* as the reference. The IRSCOPE program [28] was applied to analyze the LSC, IR and LSC boundary locations in five Boraginaceae species complete chloroplast genomes.

The complete chloroplast genomes of the five Boraginaceae plants were multiple aligned by MAFFT (v7) [29], and nucleotide variations (Pi) was calculated by DnaSP (v6) [30] with the following parameters: window length, 600; and step size, 200. The protein-coding sequences of genomes were extracted using Phylosuite software (v1.1.16) [31]. The codon preference of protein-coding genes in chloroplast genome was analyzed and the RSCU value was calculated using Mega software (v7.0) [32].

### 2.4. Repeat Sequence Analysis

MISA (v2.1) (https://webblast.ipk-gatersleben.de/misa/ (accessed on 4 May 2023) [33], TRF (v4.09 (https://tandem.bu.edu/trf/trf.unix.help.html (accessed on 4 May 2023) [34] and REPuter web server (https://bibiserv.cebitec.uni-bielefeld.de/reputer/ (accessed on 4 May 2023) [35] were used to identify the repeat sequences including microsatellite sequence repeats, tandem repeats and scattered repeats. The results were visualized using Excel (2021) software (v2207).

### 2.5. Phylogenetic Analysis

The complete chloroplast genomes of 30 species were downloaded from the National Center for Biotechnology Information (NCBI). *Isodon serra* and *Forsythia suspensa* were chosen as the outgroups. The detailed list of all the species along with their respective accession numbers for the chloroplast genomes available in the NCBI database can be found in Table S1. All the sequences were aligned using MAFFT with the default parameters. Ambiguously aligned fragments were filtered by GBLOCKS 0.91b [36], and the parameter settings were as follows: minimum number of sequences for a conserved position, 20; minimum number of sequences for a flank position, 20; maximum number of contiguous nonconserved positions, 6; minimum length of a block, 11; and allowed gap positions, 0. Based on the result of alignment, a phylogenetic tree was constructed using IQ-TREE 2 [37] with 5000 bootstraps. Bayesian inference (BI) phylogenies were conducted through MRBAYES 3.2.0 [38], employing the GTR+I+G model across eight parallel runs for a total of 2,000,000 generations. We discarded the initial 25% of the sampled data as burn-in. For visualization, the tree files were uploaded to the online tool iTOL (v6) [39].

## 3. Results

### 3.1. Chloroplast Genome Assembly and Genome Features 

**Figure 1:**
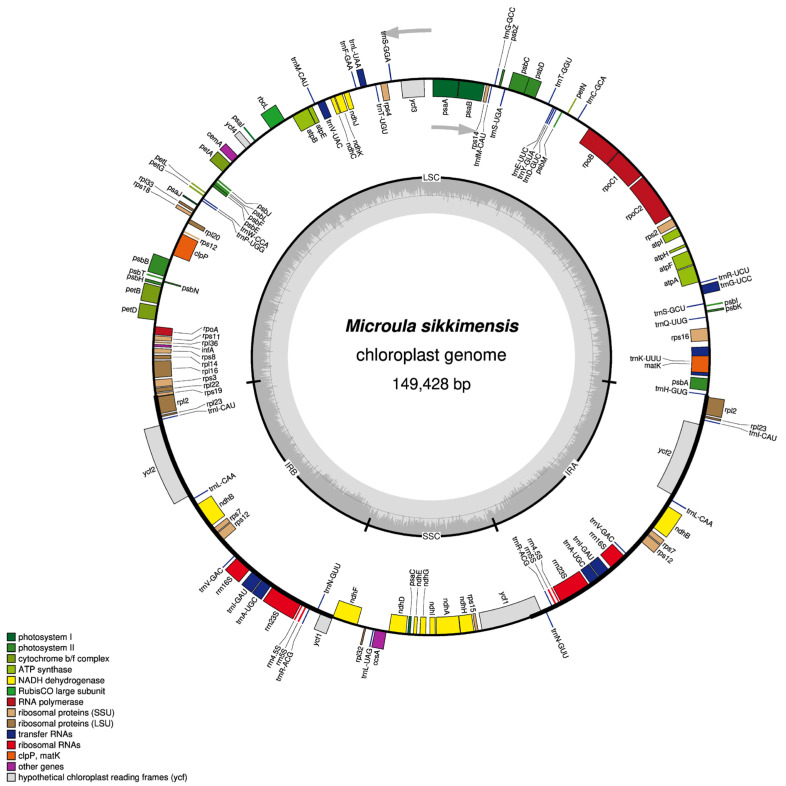
Chloroplast genome map of *M. sikkimensis*. Genes inside the circle are transcribed clockwise, while those outside the circle are transcribed anticlockwise. The large single copy (LSC) region, inverted repeat (IRA, IRB) regions and small single copy (SSC) region are shown in the figure. The darker gray in the inner circle shows the GC content, while the lighter gray shows the AT content. Genes with different functions are represented by different colors.

In *M. sikkimensis*, the chloroplast genome spans a total of 149,428 bp, characterized by a conventional quadripartite circular structure, which is divided into four specific regions: the LSC region (81,329 bp), the SSC region (17,261 bp), and two identical IR regions (25,419 bp of each), which separated SSC and LSC (Figure 1).

In the chloroplast genome of *M. sikkimensis*, gene annotation efforts revealed the presence of 112 genes in total, including 78 protein-coding genes, 30 transfer RNA (tRNA) genes, and 4 ribosomal RNA (rRNA) genes. The protein-coding genes could be grouped into 15 distinct gene families, including 16 photosystem II genes, 12 ribosome small genes, 11 NADH dehydrogenase genes, 9 ribosome major genes, 6 cytochrome b/f complex genes, 6 ATP synthase genes, 5 photosystem I genes, 4 DNA-dependent RNA polymerase genes, 3 conserved open reading frame genes, 1 1, 5-diphosphate ribulose carboxylase/oxygenase large subunit gene, 1 mature enzyme gene, 1 membrane protein gene, 1 protease gene, 1 C-type cytochrome synthesis gene, 1 translation initiation factor (Table 1). Among the 112 genes, 7 protein-coding genes, 7 tRNA genes, and 4 rRNA genes were duplicated in IR regions (Figure 1).

The chloroplast genome of *M. sikkimensis* exhibited a GC content of 37.51%. Notably, the GC content varies considerably across the different regions within the chloroplast genome. The IR region invariably displayed the highest GC content at (43.13%). This high GC content in the IR regions stands in sharp contrast to that of the LSC region, which exhibits a GC content of 35.38%, and the SSC region, which is further characterized by an even lower GC content of 30.99%.

In the chloroplast genome of *M. sikkimensis*, 12 genes contained introns, with 10 genes containing only one intron and the 2 genes harboring two introns. Among them, the intron of rpoc1 gene was the largest (1617 bp), while the intron of ycf3 gene was the smallest (153 bp) (Table S2).

Among the protein-coding genes of *M. sikkimensis*, unlike the conventional ATG initiation codon, the initiation codon of rps19 gene with GTG, while ndhD gene started with ACG (Table S3), which were similar to the other species [40,41,42,43,44].

### 3.2. Codon Usage Bias

**Figure 2:**
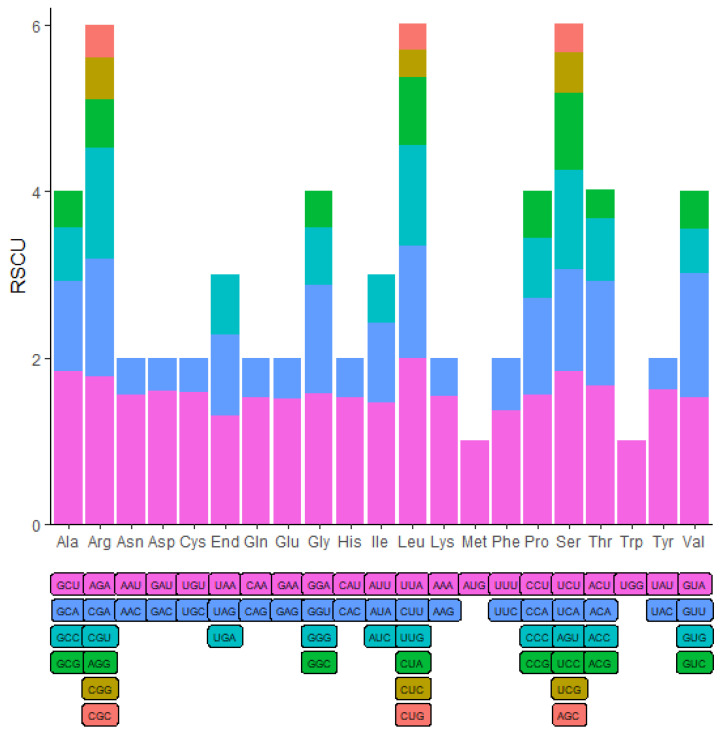
Codon content and RSCU value of the 20 amino acid and stop codons in all protein-coding genes of *M. sikkimensis* chloroplast genome.

The analysis of codon usage bias within the chloroplast genome of *M. sikkimensis*, which encompassed 78 protein-coding genes, had been conducted to help comprehend the patterns in amino acid representation. Codons that exhibit a relative synonymous codon usage (RSCU) value exceeding 1 were identified as being preferentially utilized for amino acid encoding, which allows for a more clear understanding of genomic preferences in codon selection [45]. The majority of genes displayed codon preference, with the exception of the initiation codon AUG and the tryptophan codon UGG, each of which maintained an RSCU value of exactly 1. The codon UUA, encoding for leucine, exhibited the highest RSCU value of 2. Conversely, codon CUG for leucine presented the lowest RSCU value of 0.32 (Figure 2).

### 3.3. Repeat Sequences and SSR Analysis

In the comprehensive analysis of the *M. sikkimensis* chloroplast genome, 38 SSRs were detected, distributed as follows: 26 located in the LSC region, 11 located in the SSC region, and 1 located in the IR region. Mononucleotide repeats consisted of 10–12 repetitions, dinucleotide repeats had 5–8 repetitions, trinucleotide repeats had 4 repetitions, while tetra- and pentanucleotide repeats had 3 repetitions. The analysis revealed a predominance of SSRs characterized by A/T motifs over those with G/C motifs. Specifically, A/T mononucleotide repeats were identified as the most prevalent type (*n* = 22), with AT dinucleotide-containing repeats emerging as the second most common (*n* = 8) (Table S4).

**Figure 3:**
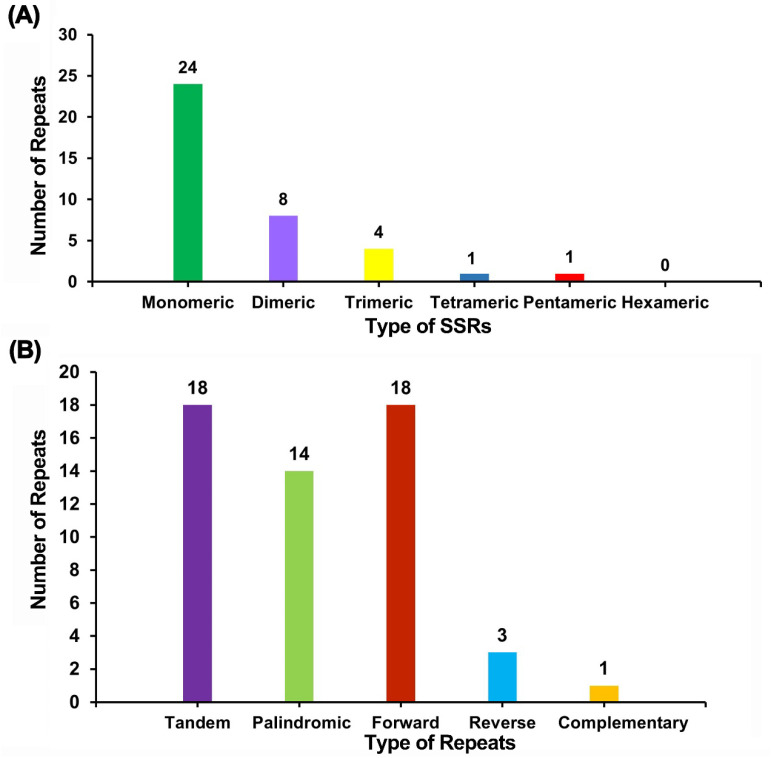
Repeat sequence and SSR analysis of *M. sikkimensis* chloroplast genome. (**A**) The horizontal coordinate represents the type of SSRs, the vertical coordinate represents the number of repeats, the green represents monomer SSRs, the purple represents dimer SSRs, the yellow represents trimer SSRs, the blue represents tetramer SSRs, and the red represents pentamer SSRs. No hexamer SSRs were detected in the chloroplast genome. (**B**) The horizontal coordinate indicates the type of repeat sequence, the vertical coordinate indicates the number of repeat segments, purple indicates tandem repeats, green indicates palindromic repeats, red indicates forward repeats, blue indicates reverse repeats, and yellow indicates complementary repeats.

Tandem repeats, often referred to as satellite DNA, consist of core sequences ranging from 7 to 200 bases in length, which are repetitively aligned in the sequence. Such repeats exist widely in eukaryotic and prokaryotes genomes. Within the chloroplast genome of *M. sikkimensis*, a total of 18 tandem repeats have been identified, each of which exhibited a similarity of over 74% and lengths ranging from 8 to 24 bp (Figure 3).

The analysis of dispersed repeat sequences within the *M. sikkimensis* chloroplast genome, conducted by REPuter, identified 36 pairs of repeats each with a minimum length of 30 base pairs. Among these pairs, 14 were identified as palindromic repeats, 18 as forward repeats, 3 as reverse repeats, and 1 as complementary repeat. Notably, the maximum length recorded for palindromic repeats was 46 bp, and for forward repeats, it extended to 52 bp (Figure 3).

### 3.4. Comparative Genome Analysis

**Figure 4:**
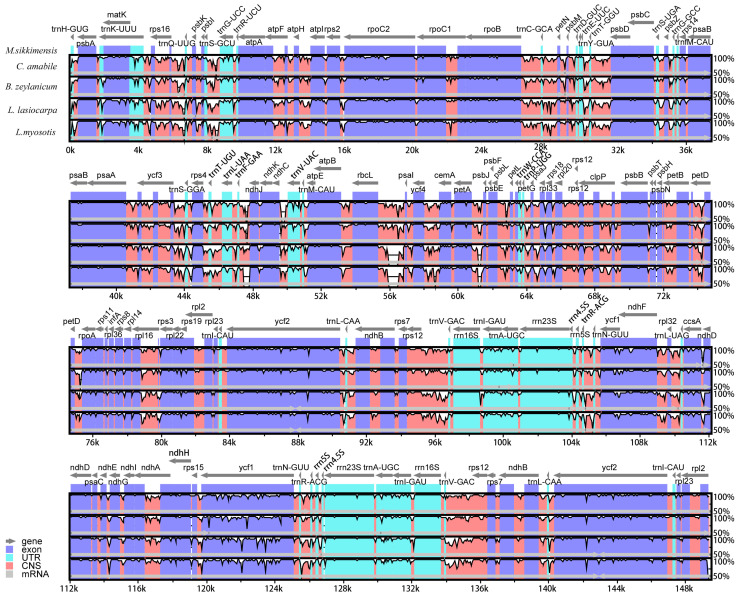
Sequence alignment of five Boraginaceae genomes in MVISTA. The grey arrows above the alignment indicate the genes transcription directions. The *Y*-axis represents identity, ranging from 50% to 100%.

Comparative analysis of the chloroplast genomes from five Boraginaceae species was conducted using the mVISTA tool, with the *M. sikkimensis* genome serving as the reference sequence. As a result, these five genomes were basically identical in coding regions, whereas were more diverse in noncoding regions. Significant variation was observed in the intergenic spacers of the chloroplast genomes, notably within regions such as *matK*-*rps16*, *rps16*-*trnQ-UUG*, *trnS*-*GCU*, *trnF-GAA*-*ndhJ*, *rbcL*-*psaI*, *ycf4*-*cemA*, and *petA*-*psbJ* in the LSC region, along with *ccsA*-*ndhD* and *rps15*-*ycf1* in the SSC region (Figure 4). These regions may serve as potential molecular markers for the identification of Boraginaceae species.

**Figure 5:**
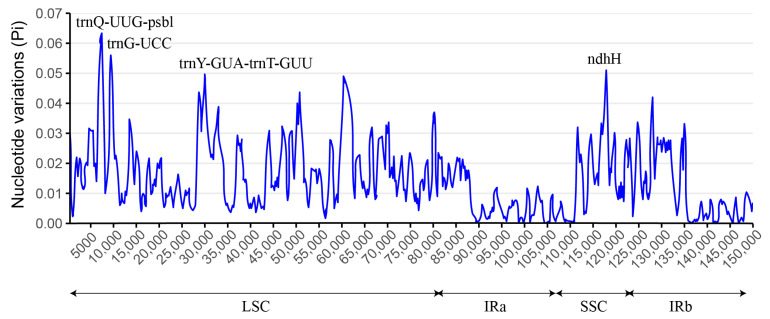
Nucleotide diversity (Pi) analysis for chloroplast genomes from the Boraginaceae plants. (window length: 600 bp; step size: 200 bp).

To evaluate the extent of genetic diversity among chloroplast genomes from five Boraginaceae species, alignments were generated, and nucleotide variability (Pi) was calculated using the DnaSP software (v6). The findings, illustrated in Figure 5, indicated that Pi values varied from 0 to 0.062, with a mean of approximately 0.013. Moreover, analysis pointed out four loci with high Pi values (≥0.05) as mutational hotspots within these species, including 1 protein coding gene (*ndhH*), 1 tRNA coding gene (*trnG-UCC*) and 2 intergenic regions (*trnQ-UUG*-*psbl* and *trnY-GUA*-*trnT-GUU*). These identified regions could serve as potential molecular markers for Boraginaceae species. Additionally, the region with the minimum Pi value was found within the IR region, indicating their significant conservation across the Boraginaceae family.

**Figure 6:**
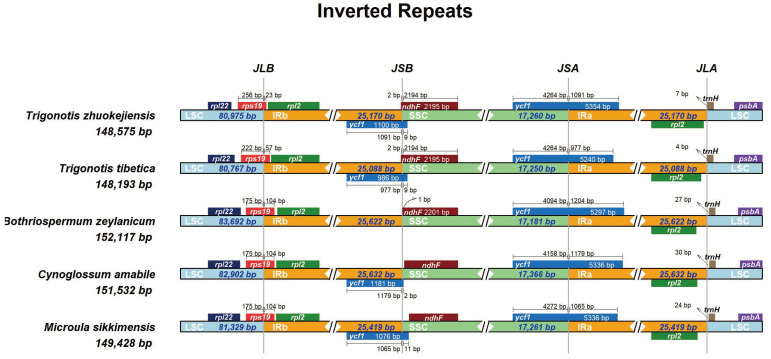
Comparison of the junction positions between the LSC, SSC and IR regions among the chloroplast genomes of five species.

The dynamics of the IR region, through its expansion and contraction, played a crucial role in plant evolutionary processes. These changes could induce structural modifications within the chloroplast genome, potentially influencing the expression and functionality of genes within the chloroplast genes [10,42,46]. The comparative analysis was performed on the boundaries of the chloroplast genome regions across five species: *M. sikkimensis*, *Bothriospermum zeylanicum*, *Trigonotis zhuokejiensis*, *Trigonotis tibetica*, and *Cynoglossum amabile*. The results revealed variations in the chloroplast genome sizes among the five examined species, with lengths extending from 148,193 bp (*T. tibetica*) to 152,532 bp (*C. amabile*). Specifically, the LSC regions displayed lengths from 80,767 bp (*T. tibetica*) to 83,692 bp (*B. zeylanicum*). The lengths of SSC regions were observed to range from 17,181 bp (*B. zeylanicum*) to 17,366 bp (*C. amabile*). In addition, the IR regions exhibited lengths from 25,088 bp (*T. tibetica*) to 25,632 bp (*C. amabile*). Across all examined species, the boundaries of the LSC-IRb and SSC-IRa regions were identified within the rps19 and ycf1 genes, respectively. Notably, the junction between the SSC and IRb regions occurred within the ycf1 gene across most species. However, an exception was observed in *B. zeylanicum*, where the ycf1 gene was absent near the IRb-SSC junction. Instead, the *ndhF* gene, located entirely within the SSC region, was found to be next to the IRb/SSC boundary, with only a 1 bp distance. In *T. zhuokejiensis* and *T. tibetica* chloroplast genomes, the boundary between the IRb and SSC regions was situated within the *ndhF* gene, which had a 2 bp insertion in IRb. Additionally, genes such as rpl22, rpl2, and *trnH* were also identified at the boundaries of LSC/IR and SSC/IR across the five species examined (Figure 6).

### 3.5. Phylogenetic Analysis

**Figure 7:**
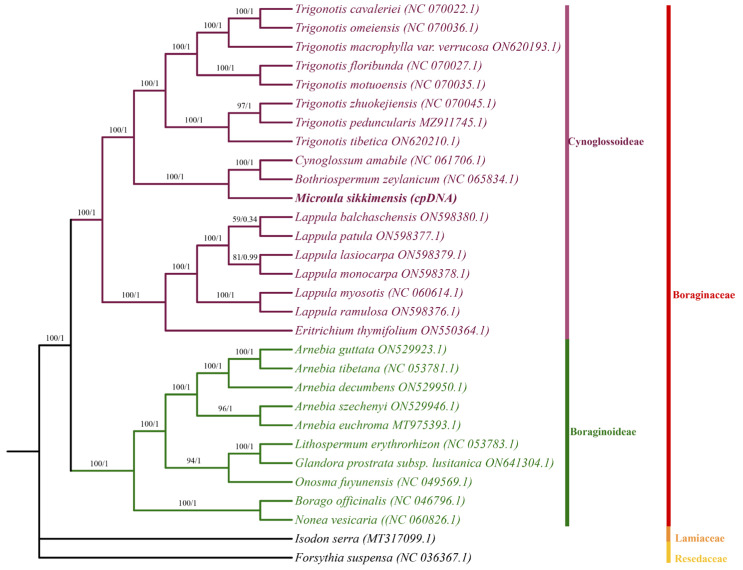
Phylogenetic tree reconstructed based on the complete chloroplast genome sequences from 30 species using ML method. The numerical annotations above the branches represented the ML bootstrap support values/BI probability support values.

Phylogenetic analyses employing ML and BI approaches were conducted on the chloroplast genome sequences of 30 Boraginaceae species, utilizing *I. serra* and *F. suspensa* as the outgroups. As shown in Figure 7, the generated phylogenetic trees demonstrated high support for most clades. This comprehensive analysis led to the categorization of the examined Boraginaceae species into two distinct subfamilies: Cynoglossoideae and Boraginoideae.

*M. sikkimensis* displayed a sister relationship with *C. amabile* and *B. zeylanicum*, but failed to cluster into the same branch, indicating significant distinctions in their chloroplast genomes, albeit with high similarity. This study had provided crucial genetic data that were essential for elucidating the phylogenetic relationships within the Boraginaceae family.

## 4. Discussion

This study had for the first time successfully sequenced the complete chloroplast genome of *M. sikkimensis*, presenting its genetic information. The analysis demonstrated that the *M. sikkimensis* chloroplast genome spanned a length of 149,428 bp, and it featured the circular quadripartite typical of chloroplast genomes, consisting of the LSC, SSC, and a pair of IR regions. Gene annotation indicated that the genome contains 112 genes, which include 78 protein-coding genes, 30 tRNA genes, and 4 rRNA genes, which is consistent with the genomic structure and gene quantity observed in the chloroplasts of other species within the Boraginaceae family [47,48,49,50,51]. Introns were crucial for the regulatory mechanisms that control gene expression. They influenced the synthesis and function of proteins through splicing regulation, thus significantly impacting the development, growth and environment adaptation of organisms [52]. In the *M. sikkimensis* chloroplast genome, our analysis identified 12 genes containing introns, 10 of these genes possess a single intron, while 2 contain a pair of introns. GC content was one of the important characteristics of the nucleic acid sequence composition, exhibiting variation across different species. This variability influenced the distribution, adaptability to environmental conditions, and lifestyles of species [53]. The chloroplast genome of *M. sikkimensis* had an overall GC content of 37.51%. Within this genome, the GC content varied considerably across different regions, reflecting the diversity in structural and functional demands of these regions. The highest GC content was found in IR regions at 43.13%, suggesting a possible role in enhancing the stability of the genome structure or in specific functional aspects related to gene expression.

SSRs, as a crucial category of molecular genetic markers, have found extensive applications across various domains of biological research. They served as important tools applied in genetic relationships, population structures, and evolutionary processes among species [54,55,56]. In plant populations, SSRs could serve as highly effective markers, facilitating the examination of genetic diversity within closely related taxa. Here, we found 38 SSRs in the chloroplast genome in *M. sikkimensis*, including the following five types of SSRs: mononucleotide, dinucleotide, trinucleotide, tetranucleotide, and pentanucleotide. A/T mononucleotide repeats were the most frequent SSR, followed by AT dinucleotide repeats, that were consistent with the previous reports [57,58]. These SSR markers provided insights for genetic diversity studies and conservation strategies in the Boraginaceae.

Research on codon usage bias could help explain the intricacies of gene expression and the mechanisms of translation. The preference for certain synonymous codons was associated with the number of introns and exhibited variability across different exons. This variability in codon usage patterns is distinct among various species. Furthermore, evidence suggests that DNA methylation plays a significant role in influencing synonymous codon usage bias [59]. Additionally, the GC content in codons is recognized as a key factor in the development of codon usage bias [60]. Within the analysis, it was observed that out of the 30 codons RSCU values above 1, 29 concluded with either A or U. Conversely, of the 32 codons showing RSCU values below 1, 29 concluded with G or C. These findings suggest a codon preference in *M. sikkimensis* for ending with A or U at the third base, a pattern also observed in species such as *Fagopyrum dibotrys* and *Salix wilsonii* [61,62]. This preference for A or T in the codon composition could stem from evolutionary pressures and genetic alterations. Within the plant chloroplast genome, it was observed that preferred synonymous codons predominantly terminated with A or U. This trend might be attributed to a higher content of A and T bases, resulting in an obvious bias for A or T ending codons [63].

By comparing the chloroplast genomes from various species, we could enhance our understanding of the evolutionary relationships and genomic structural disparities among these species [64,65]. Comparative analysis of the chloroplast genomes across five Boraginaceae species revealed notable similarities within their coding sequences alongside pronounced differences in their non-coding regions. Such variability in the non-coding regions presents their potential for use as molecular markers in future Boraginaceae investigations, providing crucial insights for species classification and phylogenetic analyses. Additionally, phylogenetic analysis revealed that *M. sikkimensis* had a sister relationship with *C. amabile* and *B. zeylanicum*. However, they failed to cluster into the same branch, indicating that they were highly similar in chloroplast genomes, but there were still discernible differences. It could be inferred that certain regions within the chloroplast genomes of these three species have experienced divergent evolutionary paths, resulting in slight but observable genetic variations.

## 5. Conclusions

This study presented a comprehensive analysis of the chloroplast genome of *M. sikkimensis*, shedding light on its genetic characteristics and evolutionary context within the Boraginaceae family.

The chloroplast genome, spanning over 149,428 bp, exhibited the characteristic circular quadripartite structure common to higher plants. The gene annotation identified a total of 112 genes, consistent with the structure observed in related Boraginaceae species. The identification of SSRs, with a predominance of A/T mononucleotide repeats followed by AT dinucleotide repeats, provided valuable genetic markers for exploring genetic diversity within closely related plant populations. Comparative analysis with other species chloroplast genomes revealed both conservation and variability, particularly in non-coding regions. These highly variable regions served as crucial molecular markers for species classification and phylogenetic research within the Boraginaceae family. Furthermore, phylogenetic analysis placed *M. sikkimensis* in a distinct relationship with *C. amabile* and *B. zeylanicum*, indicating a high degree of similarity in their chloroplast genomes. However, discernible differences existed, suggesting subtle genomic distinctions that endorse further investigation.

Overall, this study not only provided a comprehensive understanding of the chloroplast genome of *M. sikkimensis* but also established a valuable genetic resource for future research in phylogenetics, molecular marker development, and conservation strategies among the Boraginaceae family. These findings could contribute to the broader field of plant genomics and facilitate a deeper appreciation of the evolutionary dynamics within this plant family.

## Figures and Tables

**Table 1:** List of genes annotated in the chloroplast genomes of *M. sikkimensis*.

Group of Genes	Name of Genes	Number of Genes
NADH-dehydrogenase	*ndhA*, *ndhB* (×2), *ndhC*, *ndhD*, *ndhE*, *ndhF*, *ndhG*, *ndhH*, *ndhI*, *ndhJ*, *ndhK*	11
photosystem I	*psaA*, *psaB*, *psaC*, *psaI*, *psaJ*	5
photosystem II	*psbA*, *psbB*, *psbC*, *psbD*, *psbE*, *psbF*, *psbH*, *psbI*, *psbJ*, *psbK*, *psbL*, *psbM*, *psbN*, *psbT*, *psbZ*, *ycf3*	16
cytochrome b/f complex	*petA*, *petB*, *petD*, *petG*, *petL*, *petN*	6
ATP synthase	*atpA*, *atpB*, *atpE*, *atpF*, *atpH*, *atpI*	6
Large subunit of rubisco	*rbcL*	1
Small subunit of ribosome	*rps2*, *rps3*, *rps4*, *rps7* (×2), *rps8*, *rps11*, *rps12* (×2), *rps14*, *rps15*, *rps16*, *rps18*, *rps19*	12
Large subunit of ribosome	*rpl2* (×2), *rpl14*, *rpl16*, *rpl20*, *rpl22*, *rpl23* (×2), *rpl32*, *rpl33*, *rpl36*	9
DNA dependent RNA polymerase	*rpoA*, *rpoB*, *rpoC1*, *rpoC2*	4
rRNA genes	*rrn4.5S* (×2), *rrn5S* (×2), *rrn16S* (×2), *rrn23S* (×2)	4
tRNA genes	30 tRNAs	30
Maturase	*matK*	1
Envelope membrane protein	*cemA*	1
Protease	*clpP*	1
c-type cytochrom synthesis gene	*ccsA*	1
Translation al initiation factor	*infA*	1
Genes of unknown functions Open Reading	*ycf1* (×2), y*cf2* (×2), ycf4	3

## Data Availability

The data presented in this study are openly available in NCBI (GenBank accession number: OR866440).

## Supplementary Materials

The following supporting information can be downloaded at: https://www.mdpi.com/article/10.3390/genes15020226/s1, Table S1 All information of species and the accession numbers of their chloroplast genomes in NCBI; Table S2 The intron-containing genes in the chloroplast genomes of *M. sikkimensis*; Table S3 Sequences of protein-coding genes of *M. sikkimensis* chloroplast genome; Table S4 repeat sequences in the chloroplast genome of *M. sikkimensis*.
