# Supplementary material for: Comparative Analyses of Complete Chloroplast Genomes of Microula sikkimensis and Related Species of Boraginaceae
Source: Genes (Basel). 2024 Feb 10;15(2):226. doi: 10.3390/genes15020226 (PMC10887780; doi:10.3390/genes15020226)
Supplement: Supplementary file 1 [file genes-15-00226-s001.zip › Table S1 All information of species and the accession numbers of their chloroplast genomes in NCBI.pdf]

Table S1 All information of species and the accession numbers of their chloroplast genomes in NCBI

ID	Organism	Family	Subfamily	Genus	Full length (bp)
MT317099.1	*Isodon serra*	Lamiaceae	Nepetoideae	Isodon	152676
ON620210.1	*Trigonotis tibetica*	Boraginaceae	Cynoglossoideae	Trigonotis	148193
ON620193.1	*Trigonotis macrophylla var. verrucosa*	Boraginaceae	Cynoglossoideae	Trigonotis	147247
NC_070022.1	*Trigonotis cavaleriei*	Boraginaceae	Cynoglossoideae	Trigonotis	147645
NC_065834.1	*Bothriospermum zeylanicum*	Boraginaceae	Cynoglossoideae	Bothriospermum	152117
ON529923.1	*Arnebia guttata*	Boraginaceae	Boraginoideae	Arnebia	150375
NC_053781.1	*Arnebia tibetana*	Boraginaceae	Boraginoideae	Arnebia	150465
NC_053783.1	*Lithospermum erythrorhizon*	Boraginaceae	Boraginoideae	Lithospermum	149316
ON641304.1	*Glandora prostrata subsp. lusitanica*	Boraginaceae	Boraginoideae	Glandora	150041
NC_061706.1	*Cynoglossum amabile*	Boraginaceae	Cynoglossoideae	Cynoglossum	151532
NC_070027.1	*Trigonotis floribunda*	Boraginaceae	Cynoglossoideae	Trigonotis	148698
NC_070035.1	*Trigonotis motuoensis*	Boraginaceae	Cynoglossoideae	Trigonotis	148667
ON529946.1	*Arnebia szechenyi*	Boraginaceae	Boraginoideae	Arnebia	151700
NC_070036.1	*Trigonotis omeiensis*	Boraginaceae	Cynoglossoideae	Trigonotis	148671
NC_049569.1	*Onosma fuyunensis*	Boraginaceae	Boraginoideae	Onosma	150612
NC_046796.1	*Borago officinalis*	Boraginaceae	Boraginoideae	Borago	149835
NC_060826.1	*Nonea vesicaria*	Boraginaceae	Boraginoideae	Nonea	151099
ON529950.1	*Arnebia decumbens*	Boraginaceae	Boraginoideae	Arnebia	149546
NC_036367.1	*Forsythia suspensa*	Oleaceae	Forsythieae	Forsythia	156404
MZ911745.1	*Trigonotis peduncularis*	Boraginaceae	Cynoglossoideae	Trigonotis	147508
NC_060614.1	*Lappula myosotis*	Boraginaceae	Cynoglossoideae	Lappula	146668

NC_070045.1	*Trigonotis zhuokejiensis*	Boraginaceae	Cynoglossoideae	Trigonotis	148575
MT975393.1	*Arnebia euchroma*	Boraginaceae	Boraginoideae	Arnebia	150250
ON550364.1	*Eritrichium thymifolium*	Boraginaceae	Cynoglossoideae	Eritrichium	147550
ON598380.1	*Lappula balchaschensis*	Boraginaceae	Cynoglossoideae	Lappula	147561
ON598379.1	*Lappula lasiocarpa*	Boraginaceae	Cynoglossoideae	Lappula	147578
ON598378.1	*Lappula monocarpa*	Boraginaceae	Cynoglossoideae	Lappula	147558
ON598377.1	*Lappula patula*	Boraginaceae	Cynoglossoideae	Lappula	147558
ON598376.1	*Lappula ramulosa*	Boraginaceae	Cynoglossoideae	Lappula	146049
