# Supplementary material for: Comparative Analyses of Complete Chloroplast Genomes of Microula sikkimensis and Related Species of Boraginaceae
Source: Genes (Basel). 2024 Feb 10;15(2):226. doi: 10.3390/genes15020226 (PMC10887780; doi:10.3390/genes15020226)
Supplement: Supplementary file 1 [file genes-15-00226-s001.zip › Table S2 The intron-containing genes in the chloroplast genomes of M. sikkimensis.pdf]

Table S2 The intron-containing genes in the chloroplast genomes of *M. sikkimensis*

No.	Gene	Location		Length
1	rps12	IR		229
2	rps16	LSC		212
3	atpF	LSC		410
4	rpoC1	LSC		1617
5	ycf3	LSC	Intron1	228
			Intron2	153
6	cloP	LSC	Intron1	294
			Intron2	226
7	petB	LSC		642
8	petD	LSC		475
9	rpl16	LSC		396
10	rpl2	IR		431
11	ndhB	IR		758
12	ndhA	SSC		545
