# Supplementary material for: Comparative Analyses of Complete Chloroplast Genomes of Microula sikkimensis and Related Species of Boraginaceae
Source: Genes (Basel). 2024 Feb 10;15(2):226. doi: 10.3390/genes15020226 (PMC10887780; doi:10.3390/genes15020226)
Supplement: Supplementary file 1 [file genes-15-00226-s001.zip › Table S3 Sequences of protein-coding genes of M. sikkimensis chloroplast genome.pdf]

Table S3 Sequences of protein-coding genes of *M. sikkimensis* chloroplast genome

>atpA

ATGGTAACCATTCGAGCCGACGAAATTAGTAATATTATCCGTGAACGTATTGAACAATAT
AATAGAGAAGTAAAGATTGTAAATACCGGTACTGTACTTCAAGTAGGCGACGGCATTTC
TCGTATTCATGGTCTTGATGAAGTAATGGCAGGCGAATTAGTGGAATTTGAAGAGGGTA
CAATAGGCATTGCTCTTAATTTGGAATCAAATAATGTTGGTGTTGTATTAATGGGCGATG
GTTTGATGATACAGGAAGGAAGTTCTGTAAAAGCAACAGGAAGAATTGCTCAGATACC
AGTGAGTGAGGCCTATTTAGGTCGTGTTATAAATGCCCTAGCTAAACCTATTGATGGTAG
AGGTGAAATTTCAGCTTCTGAATCTCGATTAATTGAATCTCCCGCTCCAGGTATTATTTC
GCGTCGTTCCGTATATGAGCCTCTTCAAACCGGGCTTATTGCTATTGATTCAATGATCCC
TATAGGGCGTGGTCAGCGAGAATTAATTATTGGGGACAGACAGACCGGTAAAACAGCA
GTAGCTACAGATACAATTCTCAATCAACAAGGTCAAAATGTAATATGCGTTTATGTAGCT
ATTGGTCAAAAAGCGTCTTCTGTGGCCCAGGTAGTAACTACTTTACAGGAAAGGGGAG
CAATGGAATATACTATTGTGGTAGCTGAAACGGCGGATTCCCCTGCTACATTACAATATC
TCGCTCCTTATACAGGAGCAGCTTTGGCTGAATATTTTATGTATCGTGAACGACACACTT
CAATCATTTATGATGATCTCTCCAAACAAGCACAAGCTTATCGCCAAATGTCTCTTCTAT
TACGAAGACCCCCAGGTCGCGAAGCTTATCCAGGGGATGTTTTTTATTTGCATTCACGT
CTTTTAGAAAGAGCTGCTAAATTAAGTTCTAGTTTAGGTGAAGGAAGTATGACCGCCTT
ACCAATAGTGGAAACTCAATCAGGAGATGTTTCGGCTTATATTCCTACTAATGTAATTTC
CATTACTGATGGGCAAATATTCTTATCCGCCGATCTATTCAATGCTGGAATCAGACCTGC
TATTAATGTGGGTATCTCCGTTTCCAGAGTGGGGTCCGCGGCTCAAATTAAAGCTATGA
AACAAGTAGCTGGTAAATTAAAATTGGAACTGGCGCAATTCGCAGAATTAGAAGCCTT
TGCACAATTTTCTTCTGATCTCGATAAAGCTACTCAGAATCAATTGGCAAGAGGTCAAC
GATTACGTGAATTGCTTAAACAATCCCAAGCAGCTCCTCTTACGGTGGAAGAACAGAT
AATGACTATTTATACCGGAACAAACGGTTATCTTGATTCATTAGATATTGGACAGGTAAG
GAAATTTCTTGTTGAGTTACGTACTTACGTAAAAACTAATAAGCCTCAGTTCCAAGAAA
TCATATCTTCTACTAAGACATTTACTAAGGACGCGGAAGCTCTTTTGAAAGAAGCTATT
CAGGAACAAATGGACCGTTTTATACTTCAGGAACAAGCATAA

>atpB

ATGAGAATCAATCCTACTCCTTCTGGTTCTGGGGTTTCCACGCTTAAAAAAAATAACCC
GGGGCGTATCGTACAAATCATCGGTCCGGTACTAGATGTAGCCTTTTCGCCCGGCAAGA
TGCCTAATATTTATAACGCCCTTGTAGTTAAAGGTCGAGATACTGTTGGTCAACCAATTA
ATGTGACTTGTGAGGTACAGCAATTATTAGGAAATAATCGAGTTAGAGCTGTAGCTATG
AGTGCTACAGATGGTCTAACGAGGGGAATGGAAGTGATTGACACAGGAGCTCCTCTAA
GTGTTCCGGTTGGTGGAGCGACTCTGGGACGAATTTTCAACGTGCTTGGAGAGCCTGT
TGATAATTTAGGTCCTGTAGATACCCGTACAACATCTCCTATTCATAGATCCGCGCCCGC
TTTTATACAGTTAGATACAAAATTATCTATTTTTGAAACAGGAATTAAAGTAGTAGATCT
TTTAGCTCCTTATCGCCGTGGAGGAAAAATAGGGCTATTCGGGGGAGCTGGAGTGGGT
AAAACAGTACTCATTATGGAATTAATTAACAATATTGCTAAAGCTCATGGAGGTGTATCC
GTATTTGGCGGGGTGGGTGAACGTACTCGTGAAGGAAACGATCTTTACATGGAAATGA
AGGAGTCTGGAGTTATTAATGAAGAAAATATTGCAGAATCAAAAGTGGCTCTAGTTTAC
GGCCAAATGAATGAACCGCCGGGAGCTCGTATGAGAGTTGGTTTGACTGCCCTAACTA
TGGCGGAATATTTCCGGGATGTTAATGAGCAAGACGTACTTCTTTTTATCGACAATATCT

TCCGTTTCGTTCAAGCAGGATCTGAAGTATCGGCCTTATTGGGTAGAATGCCTTCCGCC
GTGGGCTATCAACCTACCCTGAGTACCGAAATGGGTACTTTACAAGAAAGAATTACTTC
TACCAAAGCAGGATCCATAACTTCTATTCAAGCAGTTTATGTACCTGCAGATGATTTAAC
TGACCCTGCCCCTGCTACGACATTTGCACATTTAGATGCTACTACCGTACTATCAAGAG
GATTAGCTGCCAAGGGAATCTATCCAGCAGTAGATCCTTTAGATTCAACGTCAACCATG
CTTCAACCTCGAATCGTTGGTGAAGAACATTATGAAACTGCGCAAAGGGTAAAGCAAA
CTTTACAACGTTACAAAGAACTTCAGGACATTATAGCTATCCTTGGGTTGGACGAATTA
TCTGAAGAGGATCGTTTAACCGTTGCAAGGGCGAGAAAAATTGAACGTTTCTTATCAC
AACCTTTTTTCGTAGCAGAAGTATTTACCGGTTCTCCAGGAAAATATGTAGGTCTAGCA
GAAACAATTAGAGGGTTTCAATTGATCCTTTCCGGAGAATTAGATGGTCTTCCTGAGCA
GGCCTTTTATTTGGTAGGTAATATCGATGAAGCTACCGCGAAAGCTATGAACTTAGAAA
TGGAGAGCTTGAAGAAATGA

>atpE
ATGACCTTAAATCTTTGTGTATTGACCCCAAATCGAATTGTTTGGGATTCAGAAGTGAA
AGAAATTATTTTATCTACAAATAGTGGTCAAATTGGCGTATTACCAAACCACGCCCCTAT
TGCCACAGCTGTAGATATAGGTATTTTGAGAATACGCCTTACTGACCAATGGGTAACGA
TGGCTCTGATGGGCGGTTTTGCTAGAGTAGGGAATAATGAGATCACTGTTTTAGTAAAT
GATGCGGAGAAAGGTAGTGACCTTGATCCACAAGAAGCTCAGCAAACTCTTGAAATA
GCGGAAGCTGATTTGAGAAAAGCTGAAGGAAAGAGACAAACAATTGAGGCAAATCTA
GCTCTCCGTCGAGCTAGGACGCGAGTAGAGGCTATCAATGCGAATGCGGCGGTGATTT
AA

>atpF
ATGAAAAATGTAACCGATTCTTTCGTTTCTTTGGGCCACTGGCCATCTGCCGGGAGTTT
CGGGGTTAATACCGATATTTTAGCAACAAATCCAATAAATCTAAGTGTAGTGATTGGTGT
ATTGATCTTTTTTGGAAAGGGAGTGTTAACCGATTTATTAGATAATCGAAAACAGAAGA
TCTTAAATACTATTCGAAATTCAGAAGAACTTCGTGGGGGGGCCATTGAACAGCTGGA
AAAAGCCCGGGTTCGCTTACGAAAAGTGGAAATAGAAGCCGAGCAATTTCGAGTAAAT
GGATACTCTGAGATAGAGCGAGAAAAATTGAATTTGATTAATTCAACTTCTAAAACTTT
GGAACAATTAGAAAATTACAAAAATGAAACTATTCAGTTTGAACAGCAAAGGGCGATT
AATCAAGTACGACAACGGGTTTTTCAACAAGCCTTACAAGGAGCTCTAGGAACTCTCA
ATAGTTGTTTAAACAGCGAGTTACATTTACGTACAATTAGTGCCAATATTGGCATTTTGG
GGGCGATGAAAGAAATAACTGATTAG

>atpH
ATGAATCCACTGATTTCTGCCGCTTCTGTTATTGCTGCTGGATTGGCTGTAGGGCTTGCT
TCTATTGGACCTGGGGTTGGTCAAGGAACTGCCGCGGGTCAAGCTGTAGAGGGTATCG
CAAGACAGCCTGAAGCTGAGGGAAAAATACGAGGCACTCTATTGCTTAGTCTAGCGTT
TATGGAAGCTTTAACAATTTATGGACTGGTTGTAGCATTAGCACTTTTATTTGCGAATCC
TTTTGTTTAA

>atpI

ATGAACGTTCTATCATGTTCCATCAACACACTAAAAAAGGGGTTATACGATATATCCGGT
GTGGAGGTAGGCCAACATTTCTATTGGCAAATAGGGGGTTTCCAGGTCCACGGTCAAG
TACTTATTACTTCTTGGGTTGTAATTGCTATCTTATTAGGTTCAGCCGCTATAGCTGCTCG
TAACCCACAAACTATTCCGACTGGTGGGCAGAATTTCTTCGAATATGTTCTTGAATTTAT
TCGAGATGTAAGTAAAACTCAAATTGGCGAAGAGTACGGTCCTTGGGTTCCTTTTATTG
GAACTATGTTTCTATTTATTTTTGTTTCTAATTGGTCAGGAGCTCTTTTACCTTGGAAAAT
AATACAATTACCTCATGGAGAGTTAGCCGCACCCACGAATGATATAAATACTACTGTAG
CTTTGGCTTTACTTACGTCAGTGGCATATTTCTATGCGGGTCTTAGCAAAAAGGGATTAA
GTTATTTCGGGAAATATATTCAGCCAACTCCAATCCTTTTACCAATCAACATCTTAGAAG
ATTTCACAAAGCCCTTATCACTTAGTTTTCGACTTTTCGGGAATATCTTAGCTGATGAAT
TAGTCGTTGTTGTTCTTGTTTCTTTAGTACCTTCAGTGGTTCCTATACCTGTCATGTTCCT
TGGATTATTTACAAGTGGTATTCAAGCTCTTATTTTTGCAACTTTAGCTGCGGCGTATATA
GGTGAATCCATGGAGGGCCATCATTGA

>ccsA
ATGATATTTTCAACCTTAGAGCATATATTAACTCATATTTCTTTTTCGATAATTGTAATTGT
AATTACAATTAAGTTGATAACCTTTTTAATTGATGAAATAGTAAAACTATATGATTCGTCA
GAAAAGGGCATGGTTATTTCTTTTTTATGTATAACAGGATTATTAATTACTCGTTGGATTT
ATTCAGGGCATTTTCCACTAAGTGATTTATATGAATCGTTAATTTTCCTTTCCTGGAGTTT
CTCCCTTATTCATATTGTTCCGTATTTAAATTTAAAAAAAAATAAAAATTGGTTAAGCAC
AATAACAGGCCCTAGTGCTATTTTTACCCAAGGTTTTGTTACTTCTTCAGGTCTTTTAAC
TGAAATACATCAATCTAAAATATTAGTACCTGCTCTTCAATCTGAATGGTTAATAATGCA
CGTAAGTATGATGATATTGGGCTACTCAGCCCTTTTATGTGGATCACTATTATCAGTAGC
ACTTTTAGTTATTACATTTCGAAAAAGAAGAAATATTTTTTTGAAGAGTAATCATTTATT
AAATACGCCATTTTTCTTTAGTGAGATCAAATCCATGAATGAAGAAAGTAATGTTTTAGT
TTTTAAAAATACTTCTTTTATTTCTGCTAAGAATTATTACAGGTATCAATTAATTCAACAA
TTGGATTATTGGAGTTATCGGGTTATTAGTCTAGGATTTATCTTTTTAACTGTGGGGATTC
TTTCGGGAGCTGTATGGGCTAACGAAGCATGGGGATCGTATTGGAATTGGGATCCGAA
GGAAACTTGGGCATTTATTACTTGGATCATATTCGCGATTTATTTGCATACTCGGACAAA
TATAAAATTGCAGAGTGTAAATTCCGCAATTGTGGCGTCAATGGGCTTTGTTCTAATTTG
GATATGCTATTTTGGGGTCAATCTGTTAGGAATAGGGCTACACAGTTACGGTTTATTTGC
ATTAACATCTAATTGA

>cemA
ATGACAAAAAAGAAAGCATTCACTCCTCTTTTCTATCTTGTATTTCTAGTATTTTTACCC
TGGTGGGTTTCTCTCTCATTTAATAAAAATATCGAATCTTGGATTACTAATTGGTGGAAT
CTCGAGAAATCCGAAATTTTCTTGAATGATATTCAAGAAAAGAGTATTGTAGAAAAATT
CATAGAATTAGAGGAACTCCTCTTCTTGGAGGAAATGGTCAAGGAATACTCGGAGATA
CATCTACAAAAACTTCATATCGGAATCCACATGGAAACGATCCAATTAATCAGGATACA
CAACGAGGATCGTATCCATACAATTTTGCACTTCTCGACAAATATAATCTGTTTTGTTATT
CTAAGCGGTTATTCTATTTTGGGTAATGAGGAACTTGTAATTCTGAATTCTTGGACTCAG
GAATTCCTATATAACTTAAGCGACACAGTCAAGGCTTTTTCTATTCTTTTATTAACTGATT
TATGTATCGGATTCCATTCACCACACGGCTGGGAACTGATGATTGGTTCTGTCTACAAA
GATTTGGGGTTTGTTCATAATGATCAAATCATATCTGGTCTTGTTTCCACTTTTCCAGTC

ATTCTCGATACTCTTTTTAAATATTGGATTTTCCGTTATTTAAATCGTCTATCTCCATCACT
TGTAGTTATTTATCATTCAATGAATGACTGA

>clpP
ATGCCCATTGGTGTTCCAAAAGTACCTTTCCGACTTCCTGGAGAGGACGATGCATCTTG
GATTGACGTATACAACCGACTTTATCGAGAAAGATTACTTTTTTTAGGCCAAGGGGTTG
ATAGCGAGATCTCGAATCAACTTATTGGTCTTATGGTATATCTTAGTATCGAGGACGATA
CCAAAGATATTTATTTGTTTATAAACTCTCCTGGGGGTTGGGTAATACCTGGAATAGCTA
TTTACGATACTATGCAATTTGTACGACCAGATGTCCATACAGTATGCATGGGGTTAGCTG
CCTCCATGGGATCTTTTCTCCTGGTCGGAGGAGAAATTACCAAACGTCTAGCATTCCCT
CACGCTCGGGTAATGATCCATCAACCTGCTAGTGGTTTTTATGAGACACAAGTGGGAG
AATTTATCTTGGAAGCGGAAGAACTGCTGAAACTGCGTGAAACCATCACAAGGGTTTA
TGTACAAAGAACGGGTAAACCATTATGGGTTGTATCCGAAGACATGGAAAGAGATGTT
TTTATGTCAGCAACAGAAGCACAAGCTTATGGAATTATTGATCTTGTAGCAGTTGAATG
A

>infA
ATGAAAGAACAAAAAATGGATTCATGAAAGTTTAATTACTGAATCGCTTCCCAACGGTAT
GTTCCGGGTTCGTTTGGATAATGCGGATCTGATTCTAGGTTATGTTTCAGGAAAGATCC
GACGTAGTTTTATACGGATACTCCCGGGAGATAGAGTCAAAATTGAAGTAAGTCGTTAT
GATTCCACCAGAGGACGTATAATATATCGACTCCGTAACAAGGATTCGAAAGATTAG

>matK
ATGGAGGAACTTCAAAGATATTTACAGCTAGATAGATCTCAACAACACGACTTTCAATA
TCCACTTATACTTCAAGAGTATATTTATGCACTTGCTCATGATCATGATTTAAATCAATCA
ATTTTTTTAGAAAATTCAGGTTATGACAAGAAATCTAGTTTACTAATTGTGAAACGTTTA
ATTACTCGAATGTATCAACAGAATTTTTTTTTTATTTCTGTTAATGATTCTAACAAAAATT
TGTATTATCAAATAATATCCGAGGGATTTGCATTTATTGTCGAAATACCATTTTCTATACG
ACTAATATCTTCTCTAGAACGGAAAAAGACATTCCAATCTCATAATTTACGATCAATTCA
TTCAATATTTCCTTTTTTAGAGGACAATCTTTCACATTTAAATTGTGTGTTAGATATACTA
ATACCCCACCCTGTCCATCCGGAAATCTTGGTTCAAACTCTTCGTTTTTGGGTAAAAGA
TGCCTCTTCTTTGCATTTATTACGATTCTTTCTACACGAGTATTGGAATACTTTTATTATCC
TAAAGAAAACTAGCTCTTCTTTTTCAAAAAAAAGAAATCAAAGATTCTTCTTATATAAT
TCTCATGTATATGAATACGAATCCCTTTTTTTCTTTCTCCGCAAACAATCTTCTCATTTAC
GATCAACATCCTCTGTAATCTTTCTTGAACGAATATATTTCTATGGAAAAATAGAGCGTC
TTGTAGAAGTCTTTTCTAAAGATTTTCAAAGCAACCTTTGGTTGTTCAAGGATCCGTTC
GTGCATTATGTTAGGTATCAAGGAAAATCCCTTTTGGCTTCAAAAGAAACTTCTTTTTTA
ATAAAGAAATGGAAATATTACATTGTTCATTTTTGGCAATGTTATTTTTACCTGTGGTCG
CGCCCAGGAAGAATCTATATAAACCATATATCCGAGCATTCCCTTGACCTTATAGGCTAT
CTTTCAAGTGTACGACTAAACCCTTCAATGGTAGGGAGTCAAATCCTAGAAAATTCATT
TCTAGTCAATAATGCTATTAAGAAATTCGATACTCTTCTTCCAATTATGCCAATGATTGCA
TCATTGGCTAAAGCTAAATTTTGTAATATATTAGGGAATCCCATTAGTAAGCCGGTTCGG
TCTGATTTATCAGATTTTGATATTATTGACCGATTTGGGCGTATATCCAGAAATCTTTCTC
ATTATCATAGTGGATCTTCCAAAAAAAAAAAGTTTGTACCGTATAAAGTATATACTTCGAC

TTTCTTGTGCTAAAACTTTGGCCCGGAAACACAAAAGTAGTGTACGTGCTTTTTTGAA
AAAATTAGGCTCGGAATTATTGGAAGAGTTCCTTACGTCGGAAGAACAAGTCCTTTCTT
TGACCTTTCCCAAAGCTTGTTCTTTACGGGGAGTCTATAGAAGTAGGATTTGGTATTTA
GATATTATTTGTATCAATGATTTAGTCAATGACCAATGA

>ndhA
ATGATAATTGATACAACAGAAATAAAAGCTATCAATTCCTTTTCCAGATTGGAATCCTTA
AAAGAACTCTACGGAATCATATGGATTCTTGTCTCTATTTTAGCTCTTGTATTAGGAATTA
TAATCGGCGTACTAGTAATTGTTTGGTTAGAAAGAGAAATTTCTGCGGGGATACAACAA
CGTATTGGTCCTGAGTATGCCGGCCCCTTGGGAATCCTTCAAGCCCTAGCAGATGGTAC
AAAACTACTTTTCAAAGAGAATATTCTTACATCTAGAGGAGATGCTCGTTTGTTTAGTAT
TGGACCATCTATAGCAGTCATATCCATTTTACTCAGTTTTTCAGTAATTCCTTTTAGCTAT
AACCTTGTTCTAACCGATCTTAGTATTGGTGTTTTTTTATGGATCGCTATTTCAAGTATTG
CTCCCCTTGGACTTCTTATGTCAGGATATGGATCAAACAATAAATATTCCTTTTTAGGTG
GTTTACGGGCTGCTGCCCAATCAATTAGTTATGAAATACCATTAACTCTATGTGTATTATC
AATATCTCTACTATCTAACAGTTTAAGTACAGTTGATATAGTTGAAGCACAATCAAAGTA
TGGTTTTTGGGGGTGGAATTTGTGGCGTCAACCTATAGGGTTTATCATTTTTCTAATTTC
TTCTCTAGCCGAGTGTGAAAGATTACCCTTTGATTTACCAGAAGCAGAAGAAGAATTA
GTAGCAGGCTATCAAACCGAATATTCCGGTATCAAATTTGGTTTATTTTACGTTGCTTCG
TATCTTAATTTATTAGTTTCTTCATTATTTGTAACAGTTCTTTACTTGGGGGGTTGGAATT
TTTCTATTCCGTATATATTCGTCCCTAGATTTATTGATATAGATATAAATAAAGCCGGTAAA
GTCTTTGGAACAATAATAGGTATCTTTATTACATTAGCTAAAACTTATTTATTCTTGTTCA
TTCCTATTGCAACAAGATGGACTTTACCGAGACTTAGAATGGACCAACTTTTAAATCTT
GGATGGAAATTTCTTTTACCTATTTCTCTAGGTAATCTATTATTAACAACTTCGTCCCAAC
TTCTTTCACTCTAA

>ndhB_copy2
ATGATCTGGCATGTACAGAATGAAAACTTCATTCTCGATTCTACGAGAATTTTTATGAAA
GCCTTTCATTTGCTTCTCTTCGATGGAAGTTTGATTTTCCCAGAATGTATCCTCATTTTTG
GCCTAATTCTTCTTCTGATGATCGATTCAACCTCTGATCAAAAAGATATACCTTGGTTAT
ATTTCATCTCTTCAACAAGTTTAGTAATGAGCATAATGGCCCTATTGTTCCGATGGAGAG
AAGAACCTATGATTAGCTTTTCGGGAAATTTCCAAACGAACAATTTCAACGAAATTTTT
CAATTTCTTATTTTACTATGTTCAACTCTATGTATTCCTCTATCCGTAGAGTACATTGAAT
GTACAGAAATGGCTATAACAGAGTTTCTCTTATTCGTATTAACAGCTACTCTAGGAGGA
ATGTTTTTATGCGGTGCTAACGATTTAATAACTATCTTTGTAGCTCCAGAATGTTTCAGTT
TATGCTCCTACCTATTATCTGGATATACCAAGAAAGATGTACGGTCTAATGAGGCTACTA
TGAAATATTTACTCATGGGTGGGGCAAGCTCTTCTATTCTGGTTCATGGTTTCTCTTGGC
TATATGGTTTATCCGGGGGAGAGATCGAGCTTCAAGAAATAGTGAATGGTCTTATCAAT
ACACAAATGTATAACTCCCCAGGAATTTCAATTGCGCTCATATTCATCACTGTAGGAATT
GGGTTCAAGCTTTCCCCAGCCCCTTCTCATCAATGGACTCCTGACGTATACGAAGACTC
TCCCACTCCAGTTGTTGCTTTTCTTTCTGTTACTTCGAAAGTAGCTGCTTCAGCTTCAG
CCACTCGAATTTTCGATATTTCTTTTTATTTCTCATCAAACGAATGGCATCTTCTTCTGGA
AATCCTAGCTATTCTTAGCATGATATTGGGAAATCTCATTGCTATTACTCAAACAAGCAT
GAAACGTATGCTTGCATATTCGTCCATAGGTCAAATCGGATATGTAATTATTGGAATAATT

GTTGGAGACTCAAATGATGGATATGCAAGCATGATAACTTATATGCTGTTCTATATCTCC
ATGAATCTAGGAACTTTTGCTTGCATTGTATTATTTGGTCTACGTACCGGAACTGATAAC
ATTCGAGATTATGCAGGATTATACACGAAAGATCCTTTTTTGGCTCTCTCTTTAGCCCTA
TGTCTCTTATCCCTAGGAGGTCTTCCTCCACTAGCAGGTTTTTTCGGAAAACTCTATTTA
TTCTGGTGTGGATGGCAGGCAGGCCTATATTTCTTGGTTTTAATAGGACTCCTTACAAGC
GTTGTTTCTATCTACTATTATCTAAAAATAATCAAGTTATTAATGACTGGACGAAACCAA
GAAATAACCCCTCACGTGCGAAATTATAGAAGATCTCCTTTAAGATCAAACAATTCCAT
CGAATTGAGTATGATTGTATGTGTGATAGCATCTACTATACCAGGAATCTCAATGAACCC
AATTATTGCAATTGCTCAGGATACCCTTTTTTAG

>ndhB
ATGATCTGGCATGTACAGAATGAAAACTTCATTCTCGATTCTACGAGAATTTTTATGAAA
GCCTTTCATTTGCTTCTCTTCGATGGAAGTTTGATTTTCCCAGAATGTATCCTCATTTTTG
GCCTAATTCTTCTTCTGATGATCGATTCAACCTCTGATCAAAAAGATATACCTTGGTTAT
ATTTCATCTCTTCAACAAGTTTAGTAATGAGCATAATGGCCCTATTGTTCCGATGGAGAG
AAGAACCTATGATTAGCTTTTCGGGAAATTTCCAAACGAACAATTTCAACGAAATTTTT
CAATTTCTTATTTTACTATGTTCAACTCTATGTATTCCTCTATCCGTAGAGTACATTGAAT
GTACAGAAATGGCTATAACAGAGTTTCTCTTATTCGTATTAACAGCTACTCTAGGAGGA
ATGTTTTTATGCGGTGCTAACGATTTAATAACTATCTTTGTAGCTCCAGAATGTTTCAGTT
TATGCTCCTACCTATTATCTGGATATACCAAGAAAGATGTACGGTCTAATGAGGCTACTA
TGAAATATTTACTCATGGGTGGGGCAAGCTCTTCTATTCTGGTTCATGGTTTCTCTTGGC
TATATGGTTTATCCGGGGGAGAGATCGAGCTTCAAGAAATAGTGAATGGTCTTATCAAT
ACACAAATGTATAACTCCCCAGGAATTTCAATTGCGCTCATATTCATCACTGTAGGAATT
GGGTTCAAGCTTTCCCCAGCCCCTTCTCATCAATGGACTCCTGACGTATACGAAGACTC
TCCCACTCCAGTTGTTGCTTTTCTTTCTGTTACTTCGAAAGTAGCTGCTTCAGCTTCAG
CCACTCGAATTTTCGATATTTCTTTTTATTTCTCATCAAACGAATGGCATCTTCTTCTGGA
AATCCTAGCTATTCTTAGCATGATATTGGGAAATCTCATTGCTATTACTCAAACAAGCAT
GAAACGTATGCTTGCATATTCGTCCATAGGTCAAATCGGATATGTAATTATTGGAATAATT
GTTGGAGACTCAAATGATGGATATGCAAGCATGATAACTTATATGCTGTTCTATATCTCC
ATGAATCTAGGAACTTTTGCTTGCATTGTATTATTTGGTCTACGTACCGGAACTGATAAC
ATTCGAGATTATGCAGGATTATACACGAAAGATCCTTTTTTGGCTCTCTCTTTAGCCCTA
TGTCTCTTATCCCTAGGAGGTCTTCCTCCACTAGCAGGTTTTTTCGGAAAACTCTATTTA
TTCTGGTGTGGATGGCAGGCAGGCCTATATTTCTTGGTTTTAATAGGACTCCTTACAAGC
GTTGTTTCTATCTACTATTATCTAAAAATAATCAAGTTATTAATGACTGGACGAAACCAA
GAAATAACCCCTCACGTGCGAAATTATAGAAGATCTCCTTTAAGATCAAACAATTCCAT
CGAATTGAGTATGATTGTATGTGTGATAGCATCTACTATACCAGGAATCTCAATGAACCC
AATTATTGCAATTGCTCAGGATACCCTTTTTTAG

>ndhC
ATGTTTCTGCTCTATGAATATGATATTCTTGGGACATTTTTAATAATATCAAGTCTTATTCC
TATTTTGGCATTTTTGATTTCAGGAGTTTTAGCCCCGATTAGCAAAGGGCCAGAGAAAC
TTTCTAGTTATGAATCGGGCATAGAACCGATGGGTGATGCTTGGTTACAATTTAGAATCC
GTTATTATATGTTTGCTCTAGTTTTTGTTGTTTTTGATGTTGAAACGGTTTTTCTTTATCC

ATGGGCAATGAGTTTCGATGTATTGGGTGTATCCGTATTTATAGAAGCTTTAATATTCGTG
CTTATCTTAATTTTTGGTTTAGTTTATGCATGGCGGAAGGGGGCGTTGGAATGGTCTTAG

>ndhD
ACGAATTACTTTCCTTGGTTAACAATAATTGTTGTTTTCCCAATATTCACTGGCTCCCTTA
TTTTCTTTCTTCCTCATAGAGGGAATAGGCTAATCCGGTGGTATACTATAAGTATATGTAT
TTTGGAACTCCTTCTAACAACTTATACATTCTGTTATCATTTTCAACCGGACGATCCATT
AATCCAATTAGTTGAGGATTCTAAATGGATCCTTTTTTTTGATTTCCATTGGAGATTGGG
AATAGACGGACTTTCTATAGGGCCTATTTTACTGACCGGATTTATCACTACTTTAGCTAC
TTTAGCGGCCCGGCCAATTACTAGGGATTCTCGATTATTCCATTTCCTGATGTTAGCAAT
GTACAGCGGTCAAATAGGATTATTTTCTTCTCAGGACCTTTTACTTTTTTTCATCATGTG
GGAGTTAGAATTAATTCCTGTTTATTTACTTCTATCTATGTGGGGAGGAAAGAAACGTCT
GTACTCAGCTACAAAATTTATTTTGTACACTGCAGGAGGTTCCGTTTTTCTATTAATAGG
AGTTCTAGGTATTGGTTTATATGGCTCTAACGAGCCAACATTAAATTTTGAAACATCAGC
TAATCAGTCGTATCCTGCGGTCTTAGAAATAATATTCTATATTGGATTTTTTATTGCTTTTG
CTGTCAAATCGCCGATTATACCCCTACATACATGGTTACCAGATACCCACGGAGAAGCG
CATTACAGTACTTGTATGCTTCTAGCTGGAATCTTATTAAAAATGGGAGCATATGGGTTG
GTTCGTATCAATATGGAACTATTACCTCACGCCCATTCTATATTTGGCCCTTGGTTGGTAA
TAGTAGGTACCTTGCAAATCATCTATGCAGCTTTAACATCTCTCGGCCAACGGAATTTAA
AAAAAAGAATAGCCTATTCCTCTGTATCCCATATGGGTTTCATAATTATAGGAATAGGTT
CTATAACAGATACAGGACTGAATGGAGCCTTTTTACAAATAATCTCTCATGGATTTATTG
GTGCTGCGCTTTTTTTCTTGGCAGGAACAACCTATGATAGAATTCGTCTTGTTTATCTTG
ACGAAATGGGGGGAATAGCTATCCCGCTGCCAAAAATATTCACAATGTTTAGTAGCTTT
TCCATGGCTTCCCTTGCATTACCCGGTATGAGTGGTTTTGTTGCCGAGTTGATAACATTT
TTTGGAATAATTACCAGCCAAAAATATCTTTTAATCCCAAAAATACTAATTACTTTTGTA
ATGGCAATTGGAATGATATTAACTCCTATTTATTTATTATCTATGTTACGCCAGATGTTCTA
TGGATATAAGCTATTTAATGCCCCAAACTCTTATTTTTTTGATTCTGGACCGCGAGAATT
ATTCCTTTCTATTTGTATCTTTTTACCCGTAATAGGTATTGGTATGTACCCCGATCTTGTTC
TTTCATTTTCGATGGATAAGGTTGAAGTTATCCTATCTAATTCCTTTTATAGATAG

>ndhE
ATGATACTCGAACATGTACTTGTTTTGAGTGCCTATTTATTTTCTATCGGTATCTATGGAT
TGATCACAAGTCGAAATATGGTTAGAGCCCTTATGTGTCTGGAACTTATATTAAATGCGG
TTAATATCAATTTTGTAACGTTTTCTGATTTTTTTGATAATCGTCAATTAAAAGGAGACAT
TTTCTCCATTTTTATTATAGCTATTGCAGCCGCTGAAGCAGCTATTGGACTGGCTATTGTT
TCATCAATTTATCGTAATAGAAAATCAACCTGTATCAACCAATCAAATTTGTTGAATAAA
TAG

>ndhF
ATGGAACAGACATATCAATATGCGTGGATCATACCCTTGCTTCCACTTATGGTTCCTATG
TTAATAGGAGTGGGGCTTCTTCTTTTTCCGACAGCAACAAAAAATATTCGCCGTATATG
GGCTTTTCTGAGTATTTTATTATTAAGTATAGTCATGATTTTTTCAATCAATTTGTCTATTC
AACAAATCAATAGAAGTTCCATCTACCAATATATATGGTCTTGGACCATTAATAATGGTT
TTTCTTTAGAATTCGGCTACTTGATAGACCCACTTACTTCTATTATGTCAATATTAATCAC

TACTGTTGGAATTATGGTTCTTATTTATAGTGATAATTATATGTCTCATGATCAAGGATATT
TGAGATTTTTTGCTTATATGAGTTTTTTCAGTACGTCCATGTTAGGATTAGTTACTAGTTC
TAATTTGATACAAATTTATATTTTTTGGGAATTAGTTGGAATGTGTTCCTATCTATTAATAG
GATTTTGGTTTACACGACCTGCTGCGGCAAATGCTTGTCAAAAAGCGTTTGTAACTAAT
CGTTTAGGCGATTTTGGTTTATTATTAGGAATTTTAGGTTTTTATTGGATAACAGGCAGTT
TCGAATTTCAAGATTTATTTGAAATATTCAAAAATTTGATTTCGAACAATGAGGTTAATT
TGATATTTGCTACTTTATGTGCAGTTCTATTATTTGCGGGTGCAATTGCTAAATCTGCACA
ATTTCCACTTCATGTATGGTTACCTGATGCTATGGAGGGACCTACCCCTATTTCGGCTCT
TATCCATGCCGCGACTATGGTAGTCGCGGGAATTTTTCTTGTAGCTCGCCTTCTTCCTCT
TTTCATAGTTATACCCTATATAATGAATTTTATCGCATTGATAGGAATAATAACAGTATTCT
TAGGAGCGACTTTATCTCTTGCTCAAAAAGACATTAAAAGGGGCTTAGCCTATTCTACA
ATGTCTCAATTGGGTTATATGATGTTAGCTCTGGGTATGGGGTCTTATCGAAATGCTTTAT
TTCATTTGATTACTCATGCTTATTCCAAAGCATTATTATTTTTAGGATCTGGATCTGTTATT
CATTCAATGGAAACTATTGTTGGTTATTCTCCAGATAAAAGTCAGAATATGGTTCTTATG
GGAGGTTTAACAAAACATGTACCGATTACCAAAACGTCTTTTTTATTAGGTACACTTTC
TCTTTGTGGTATTCCACCTCTTGCCTGTTTTTGGTCCAAGGATGAAATTCTTAATGATAG
TTGGTTGTATTCACCGATTTTCGCAATAATAGCTTGGTCAACAGCAGGATTAACCGCATT
TTATATGTTTCGTATCTATTTACTTACTTTTGAGGGACATTTAAATGTCCATTTTCAAAAC
TACAGTGGTAAACAAAATACCCTCTCCTATTCCCTATCTCTATGGGGTAAAGGGGGTTC
GAAAGAAATTAACAAAAATTTTCATTTGAATAATAAAGAAAATGTTTCTTTTTTTTCAA
AAAAAATATATAAAAATGGTGAGAATGTAAGAAGCATGAAGAGGCCTTTTATTAGTATT
GCTCAGTTTCAGAATAAAAACATTTTTTTCTATCCTTATGAATCGGACAATACTATGTTA
CTTCCCCTAATTATATTAGGTCTATTTACTTTGTTTGTTGGATGTTTAGGAATTCCTTTCA
ATCAAGAGAGAGCAGATATATTAACTAAATGGTTAGCTCCATCTATAAACCTTTTGAATC
AAGAATCAGAAAATTCAATGGATTGGTATGAATTTTTAAAAGATGCTTTTTTTTCAGTC
AGTATAGCTTATTTCGGAATATTTTTAGCTTCCTTTTTATATAAACCTATTTATTCTTCTTT
ACAAAATTTTAACTTAATTAATTCATTTGTTAAAAGAGGTCCAAAGAGAATTCTTTTGG
ACAAAATTATATATGGTATATATGATTGGTCATATAATCGTGCTTACATAGATTCTTTTTAT
AAAAAATATTTAACAGAGGAGGTAAGGGGATTAGCCAAATTAACTCATTTTTTTGATCG
ACGAGTAATTGATGGAATTACAAATAGCGTTGGGATTATGAATTTTTTTGTAGGAGAAG
GTATAAAATATTTAGGAGGTGGTCGTATCTCTTCATATCTTTTCTTGTATTTTTTTTATGTA
TCAACCTTTTTATTACTAATTAGTACCAATTAG

>ndhG
ATGGATTTACCTGGGCCAATACATGAATTTCTTTTAGTCTTTCTGGGATCGGGTCTTATAT
TAGGAGGTCTAGGAGTAGTAGTACTTCCCAATCCAATTTATTCTGCCTTTTCATTGGGAT
TGGTTCTTGTCTGTATATCTCTATTCTATATTCTATCGAACTCCTATTTTGTAGCTGCCGCG
CAACTCCTTATTTACGTAGGAGCTATAAATGTTTTAATCATTTTTGCTGTTATGTTCATGA
ATGGTTCAGAATATTACAAGGATTTTCATCTTTGGACTGTGGGGGATGGAGTTACTTCG
ATAGTTTGTACAAGTCTTTTTATTTCACTAATTACTACTATTCTAGATACGTCATGGTACG
GGATTATTTGGACTACAAAATCAAATCAGATTGTAGAGCAAGATTTGATAAGTAATAGT
CAACAAATTGGAATTCATTTATCAACAGATTTTTTTATTCCATTCGAATTCATTTCAATAA
TTCTTTTAGTTGCTTTAATAGGTGCAATTGCTGTAGCTCGTCAATAA

>ndhH
ATGACTGCGCCAGTTACTACAAGAAAAGACCTCATGATAGTAAATATGGGTCCTCAGCA
CCCATCAATGCATGGTGTTCTTCGACTCATAGTTACTCTAGATGGTGAAGATGTTATTGA
CTGTGAACCAATATTGGGTTATTTACATAGAGGGATGGAGAAAATTGCGGAAAACCGA
ACAATTATACAATATTTGCCTTATGTAACACGTTGGGATTATTTAGCTACTATGTTCACAG
AAGCAATAACTGTAAATGGACCCGAACAGTTAGGAAATATTCAAGTACCTAAAAGGGC
TAGCTATATCAGAGTCATTATGTTGGAGTTGAGTCGTATAGCTTCTCATTTGTTATGGCTA
GGCCCTTTTATGGCAGATATTGGGGCACAGACCCCCTTCTTCTATATTTTTCGGGAAAG
AGAATTAATATATGACCTATTCGAAGCTGCTACTGGTATGCGAATGATGCATAATTATTTT
CGTATTGGAGGAGTAGCTGCTGATCTACCTTATGGCTGGATAGATAAATGTTTGGATTTT
TGCGATTACTTTTTAACAAGGGTTACTGAGTATCAAAAGCTTATTACACGGAATCCTATA
TTTTTAGAACGTGTTGAAGGTGTAGGCATTATTGGTGTAGAAGAAGCAATAAATTGGGG
TTTATCAGGACCAATGCTACGAGCTTCCGGAATACAATGGGATCTTCGTAAAGTTGATC
GTTATGAGTGTTACGACGAATTAGATTGGAAGGTTCAATGGCAAAAAGAGGGGGATTC
ATTAGCTCGTTATTTAGTACGAATCGGTGAAATGACGGAATCGATAAAAATTATTCAGCA
GGCTCTGGAAGGAATCCCAGGGGGTCCCTATGAAAATTTAGAAACCCGGCGTTTTGTT
AGAATAAACGATCCCGAATGGAATGATTTTGAATATCGATTTTTTAGTAAAAAACCTTCT
CCGACTTTTGAATTGTCGAAACAAGAACTTTATGTGAGAGTCGAAGCCCCAAAAGGAG
AATTGGGAATTTTTTTGATAGGAGATCAGACTGTTTTTCCTTGGAGATGGAAAATCCGA
CCGCCGGGTTTTATCAATTTGCAAATTCTTCCTCATTTAGTTAAAAGAATGAAATTGGCT
GATATTATGACAATACTAGGTAGCATAGATATCATTATGGGAGAAGTTGATCGTTGA

>ndhI
ATGTTCCCTATGGTAACTGAGTTCAAAAATTATGGTCAACAAACAATACGAGCAGCCCG
GTACATCGGTCAAGGTTTCATGATTACCTTGTCCCACGCGAATCGTTTACCAGTAACGA
TTCAATATCCCTATGAAAAATTGATCACCTCGGAACGTTTCCGAGGCCGAATCCACTTT
GAATTTGATAAATGTATTGCTTGTGAAGTATGTGTTCGTGTATGTCCTATAGATCTACCTG
TTGTTGATTGGAAATTGGAAACTTATATTCGAAAGAAACGCTTGCTTAATTACAGTATTG
ATTTTGGAGTCTGTATATTTTGTGGTAATTGTGTTGAGTATTGTCCAACTAATTGTTTATC
AATGACTGAAGAATATGAACTTTCTACCTATGATCGTCATGAATTGAATTATAATCAAAT
TGCGTTGGGTCGGTTACCGGTATCGGTAATTGATGATTATACAATTCGAATAATTTCGAA
TTCCCCTCAAATAAAAAAATAG

>ndhJ
ATGCAGGGTCGTTTGTCTGCTTGGCTAGTCAAGCATGGGGTAATTCATAGATCTTTGGG
CTTTGATTACCAAGGAATAGAGACTTTACAAATAAAGCCCGAGGATTGGCATTCCATTG
CTGTCATTTTATATGTATATGGTTACAACTATCTACGCTCCCAATGTGCATATGATGTAGC
ACCCGGCGGACTGTTAGCTAGTGTGTATCATCTTACGAGAATAGAATATGGTGTGGATC
AGCCAGAAGAGGTATGCATAAAAGTATTTACCCCAAGGAAGGATCCGAGAATTCCGTC
TGTTTTCTGGGTTTGGAAAACCGTGGATTTTCAAGAACGAGAATCTTATGATATGTTGG
GAATCTATTATGATAATCATCCACGCTTGAAACGTATCTTAATGCCTGAAAGTTGGATAG
GATGGCCCCTACGTAAAGATTATATTGCCCCCAATTTTTATGAAATACAAGATGCTCATT
GA

>ndhK
ATGAATTCCATTGAGTTTCCTTTACTTGATCGAACAACCCCCAATTCAGTTATTTCAACT
ACATTAAACGATCTTTCAAATTGGTCAAGACTCTCCAGTTTATGGCCACTTCTCTACGG
TACCAGCTGCTGCTTCATTGAATTTGCTTCACTAATAGGCTCACGATTCGACTTTGATCG
TTATGGATTAGTGCCAAGATCAAGTCCTAGACAAGCAGATCTAATTTTAACAGCCGGAA
CGGTAACAATGAAAATGGCCCCTTCTCTAGTGAGATTATATGAACAAATGCCAGAACCA
AAATATGTTATTGCTATGGGAGCATGTACAATCACAGGGGGAATGTTCAGTACCGATTCT
TATACTACTGTTCGAGGGGTCGATAAACTAATTCCTGTGGATGTTTATTTGCCTGGCTGT
CCACCTAAACCGGAAGCTGTTATAGATGCTATAACAAAACTTCGTAAGAAAATATCTCG
AGAAATCTATGAAGATAGAATTGGGTCTCGACAAGCGAATCGGTGTTTTACTACCACTC
ACAAGTTTCATGTTGGAGGCAGTATTCATACTGGAAATTACGATCAAAGATTTCTTTATC
AGCTGTCATCTACTTCAAAGATCCCTACTGGGAAACTTTTTTCAAATACAAAAGTTCAG
GATCTTCCCACGAATTAG

>petA
ATGCAAACTAGAAATACTTTTTCTTGGATAAAGGAACGGATTACTCGATCTATTTCCGTA
TCGCTTATGATATATATCATAACTTGGACATCCATTTCAAGTGCATATCCCATTTTTGCAC
AGCAGGGTTATGAAAATCCACGAGAAGCAACTGGGCGTATTGTATGTGCCAATTGTCAT
TTAGCTAATAAGCCCGTGGAGATTGAAGTTCCACAAGCAGTACTTCCTGATACTGTATT
TGAAGCAGTAGTTCGAATTCCTTATGATATGCAACTGAAACAAGTTCTTGCTAATGGTA
AAAAAGGGGGCTTGAATGTGGGGGCTGTTCTTATTTTACCGGAGGGGTTTGAATTAGC
CCCTCCCGATCGTATTTCTCCGGAGCTGAAAGAAAAAATGGGCAATTTGTCTTTTCAGA
ACTATCGCCCCAATAAAAAAAATATTCTTGTGGTAGGCCCAGTCCTTGGTAAGAAATAT
AGTGAAATCATCTTTCCTATTCTTTCCCCCGACCCTGCTACTAAGAAGGACGTTCACTTT
CTAAAATATCCTATATACATAGGTGGGAACAGGGGTCGAGGTCAGATTTATCCCGACGG
GAGCAAGAGTAACAATACAGTTTATAATGCTACAGCAGCAGGTATAGTAAATAAAATCA
TACGAAAAGAGAAAGGGGGGTATGAAATAACCATAACAGAAACAGATGCATCGGATG
AACGTCAAGTGGTTGATATTATCCCTCCAGGACCAGAACTTCTTATTTCAGAGGGCGAA
TCTATCAAATTGGATCAACCATTAACGAGTAATCCTAATGTGGGTGGGTTTGGTCAGGG
AGATGCAGAAATAGTACTTCAAGACCCATTACGTGTTCAAGGCCTTTTGTTCTTCTTGG
CATCTGTTATTTTGGCACAAATATTTTTGGTTCTTAAAAAGAAACAGTTTGAGAAGGTT
CAATTGGCAGAAATGAATTTCTAG

>petB
ATGAGTAAAGTCTATGATTGGTTCGAAGAACGTCTTGAGATTCAGGCGATTGCGGATGA
TATAACTAGTAAATACGTTCCTCCTCACGTCAATATATTTTATTGTTTAGGGGGAATTACA
CTTACTTGTTTTTTAGTACAAGTAGCTACAGGGTTTGCTATGACTTTTTACTATCGTCCG
ACCGTTACTGAGGCCTTTGCTTCTGTTCAATACATAATGACTGAAGCTAACTTTGGTTG
GTTAATCCGATCAGTTCATCGATGGTCGGCAAGTATGATGGTCTTAATGATGATCCTGCA
CGTCTTTCGTGTGTATCTCACCGGTGGATTTAAAAAACCTCGCGAATTGACTTGGGTTA
CAGGTGTGGTTTTGGGTGTATTGACCGCATCTTTTGGCGTAACTGGTTATTCCTTACCTC
GGGACCAAGTCGGTTATTGGGCAGTGAAAATTGTAACAGGTGTACCTGAAGCTATTCC
TTTAATAGGATCACCTTTGGTAGAATTATTGCGCGGAAGTGCTAGTGTGGGACAATCCA

CTTTGACTCGTTTTATAGTTTACACACTTTTGTATTGCCGCTTCTGACTGCCGTATTTAT
GTTAATGCACTTTCCAATGATACGTAAACAAGGTATTTCTGGTCCTTTATAG

>petD
ATGGGAGTAACAAAAAAACCAGACTTGAATGATCCTGTATTAAGAGCAAAATTGGCTA
AAGGGATGGGTCATAATTATTACGGAGAGCCCGCATGGCCCAACGACCTTTTATATATTT
TTCCAGTAGTCATTTTAGGTACTATTGCGTGTAACGTAGGCTTAGCGGTTCTAGAACCAT
CAATGATTGGCGAACCAGCAGATCCATTTGCAACTCCTTTGGAGATATTACCGGAATGG
TATTTCTTTCCCGTATTTCAAATACTTCGTACAGTGCCCAATAAATTATTGGGTGTTCTTT
TAATGGTTTCAGTACCCGCTGGATTATTAACAGTACCTTTTTTAGAAAATGTTAATAAAT
TTCAAAATCCATTTCGGCGTCCAGTAGCGACAACCGTTTTTTTGATCGGTACTGTCGTT
TCCCTTTGGTTAGGGATTGGTGCAACATTACCTATTGATAAATCCCTAACTTTAGGTCTT
TTTTAA

>petG
ATGATTGAAGTTTCTCTATTTGGAATCGTGTTAGGCCTAATTCCTATTACTTTGGCTGGAT
TATTCGTAACTGCATATTTACAATACAGGCGAGGTGATCAGTTGGACCTTTGA

>petL
ATGCTTACTATAACCAGTTATTTCGGTTTTCTACTAGCGGCTTTAACTATAACGTCAGCT
CTATTGATTGGTCTGAGCAAAATACGACTTATTTAA

>petN
ATGGATATAGTCAGTCTTGCTTGGGCTGCTTTAATGGTAGTCTTTACATTTTCCCTTTCAC
TCGTAGTATGGGGAAGAAGCGGCCTCTAG

>psaA
ATGATTATTCGTTCGCCGGAACCAGAAGTCAAAATTTTGGTAGATAGGGATCCCGTAAA
AACTTCTTTCGAGGAATGGGCCAAACCGGGCCATTTTTCAAGAACAATAGCTAAGGGG
CCTGATACTACCACATGGATCTGGAACCTACATGCGGATGCTCACGATTTCGATAGCCAT
ACCAGTGATTTGGAGGAGATCTCTCGAAAAGTATTTAGTGCCCATTTCGGGCAACTCTC
CATCATCTTTCTTTGGCTGAGCGGCATGTATTTCCACGGTGCTCGTTTTTCCAATTATGA
AGCGTGGCTAAGTGATCCAACTCACATTGGACCTAGTGCCCAGGTGGTTTGGCCAATA
GTGGGCCAAGAAATATTGAATGGTGATGTGGGCGGCGGTTTCCGAGGAATACAAATAA
CCTCTGGTTTTTTTCAGATTTGGCGAGCATCTGGAATAACTAGTGAATTACAACTTTATT
GTACTGCAATTGGTGCATTGATCTTTGCAGCGTTAATGCTTTTTGCTGGTTGGTTTCATT
ATCATAAAGCAGCTCCAAAATTGGCCTGGTTTCAAGATGTGGAATCTATGTTGAATCAC
CATTTAGCGGGGCTACTAGGACTTGGGTCTCTCTCTTGGGCGGGGCATCAAGTGCATGT
ATCTTTACCGATTAACCAATTTCTAAACGCTGGAGTAGATCCTAAAGAGATACCACTTC
CTCATGAATTTATATTGAATCGGGATCTTTTGTCTCAACTTTATCCCAGTTTTGCCGAAG
GAGCAACCCCCTTTTTCACCTTGAATTGGTCAAAATATGCGGAATTTCTTACTTTTCGTG
GAGGATTAGATCCAGTAACAGGGGGTCTATGGCTGACTGATATTGCTCATCATCATTTAG
CTATTGCAATTGTTTTTCTGATAGCGGGTCACATGTATAGGACCAACTGGGGCATTGGTC
ATGGTATAAAAGATATTTTAGAAGCTCATAAAGGTCCATTTACAGGTCAAGGCCATAAA

GGCCTATATGAGATCCTAACAACGTCATGGCATGCTCAATTATCTCTTAACCTAGCTATG
TTAGGCTCTTTAACCATTGTTGTAGCTCACCATATGTATTCCATGCCGCCTTATCCATATC
TAGCTACTGACTATGGTACACAACTCTCATTGTTCACACATCACATGTGGATTGGTGGAT
TTCTCATAGTTGGCGCTGCTGCGCATGCAGCCATTTTTATGGTAAGAGACTATGATCCAA
CTACTCGATACAACGATCTATTAGATCGTGTCCTTAGACATCGTGATGCAATCATATCAC
ATCTTAACTGGGCATGTATCTTTCTAGGCTTTCACAGTTTTGGTTTGTATATTCACAATGA
TACCATGAGCGCTTTAGGGCGTCCTCAAGATATGTTTTCAGATACCGCTATACAATTACA
ACCCGTTTTTGCTCAATGGATACAAAACACCCATGCTTTAGCACCTGGTGCAACAGCTC
CTGGTGCAACAGCAAGCACCAGTTTAACTTGGGGGGGTGATTTATTAGTAGCAGTGGG
CAGCAAAGTGGCTTTGTTACCTATTCCATTAGGAACCGCGGATTTTTTGGTACATCATAT
TCATGCATTTACGATTCATGTGACAGTTTTGATACTCCTGAAAGGAGTTTTATTTGCTCG
TAGCTCCCGTTTAATACCAGATAAAGCAAATCTTGGTTTTCGTTTTCCTTGTGATGGACC
TGGAAGAGGGGGTACATGTCAAGTATCGGCTTGGGATCATGTCTTCTTAGGACTATTCT
GGATGTACAATGCAATTTCGGTAGTAATATTCCATTTCAGTTGGAAAATGCAGTCAGAT
GTTTGGGGCAGTATAAGCGATCAAGGGGTAGTCACTCATATCACGGGAGGAAACTTTG
CGCAGAGTTCTATTACTATTAATGGGTGGCTCCGCGATTTCTTATGGGCACAGGCATCTC
AGGTAATTCAGTCTTATGGTTCTTCATTATCTGCATATGGTCTTTTTTTCCTAGGTGCTCA
TTTTGTATGGGCTTTTAGTTTAATGTTTCTATTCAGTGGACGTGGTTATTGGCAAGAACT
TATTGAATCTATCGTTTGGGCTCATAATAAATTAAAAGTTGCTCCTGCTACTCAGCCGAG
AGCCTTGAGCATTGTACAAGGACGTGCTGTAGGAGTAACCCATTACCTTCTGGGTGGA
ATTGCCACAACATGGGCGTTCTTCTTAGCAAGAATTATTGCAGTAGGATAA

>psaB
ATGGCATTAAGATTTCCAAAGTTTAGCCAAGGCTTAGCTCAGGACCCCACTACTCGTCG
TATTTGGTTTGGTATTGCTACCGCACATGACTTTGAGAGTCATGATGATATTACTGAGGA
ACGTCTTTATCAGAATATTTTTGCTTCTCACTTCGGTCAATTAGCAATAATTTTTCTGTGG
ACTTCTGGAAATCTGTTTCACGTTGCTTGGCAAGGCAATTTTGAGTCATGGGTACAGGA
CCCTTTACATGTAAGACCTATTGCTCATGCAATTTGGGATCCTCATTTTGGTCAACCGGC
TGTGGAAGCTTATACTCGAGGGGGTGCTCTTGGCCCAGTGAATATCGCTTATTCTGGCG
TTTATCAGTGGTGGTATACTATTGGTTTACGTACTAATGAAGATCTTTATACTGGAGCTCT
TTTTCTATTATTTCTTTCTGCCATATTCTTAATAGCGGGTTGGTTACATCTACAACCGAAA
TGGAAACCGAGCGTTTCCTGGTTCAAAAATGCTGAATCTCGTCTAAATCATCATTTATC
AGGACTCTTTGGTGTAAGTTCCTTGGCTTGGACAGGGCATTTAGTGCATGTCGCTATTC
CTGGATCCAGAGGGGAGTATGTTCGATGGAATAATTTCTTAGACGTATTACCGCATCCCC
AAGGGTTAGGCCCCCTTTTTACAGGTCAATGGAATCTTTATGCTCAAAACCCCGATTCA
AGTGGTCATTTATTTGGGACCTCCCAAGGGGCGGGAACTGCCATTCTAACCCTTCTCGG
GGGATTCCATCCACAAACGCAAAGTTTATGGCTGACTGATATGGCTCATCATCATTTAGC
TATTGCAATTGTTTTTCTCGTTGCTGGTCATATGTATAGAACTAATTTCGGGATTGGTCAT
AGTATAAAAGATCTTTTAGATGCACACATTCCTCCGGGGGGACGGTTGGGACGCGGGC
ATAAGGGTCTTTATGACACAATCAATAATTCGCTTCATTTTCAATTAGGCCTTGCTCTAG
CTTCTTTAGGGGTTATTACTTCCTTGGTAGCTCAACACATGTACTCTTTACCTGCTTATGC
ATTCATAGCACAAGATTTTACTACCCAAGCTGCATTATATACTCATCACCAATATATCGCA
GGCTTCATTATGACAGGAGCTTTTGCTCATGGAGCTATATTTTTCATTAGAGATTACAAT
CCGGAGCAAAATGAAGATAATGTATTAGCGAGAATGTTAGAGCATAAAGAAGCTATCAT

ATCTCATTTAAGTTGGGCCAGCCTCTTCCTGGGATTCCATACTTTAGGACTTTATGTTCA
TAATGATGTCATGCTTGCCTTCGGTACTCCAGAGAAGCAAATCTTGATCGAACCCATATT
TGCTCAATGGATACAATCTGCTCACGGTAAAACTTCATATGGGTTCGATGTACTTTTATC
TTCAACGAGCGGCCCGGCATTTAATGCCGGTCGAAGCATCTGGTTGCCTGGGTGGTTA
AATGCTATTAATGCAAATAGTAATTCATTATTCTTAACAATAGGTCCTGGAGACTTTTTAG
TTCACCATGCTATTGCTCTAGGTTTACATACAACTACATTGATCTTAGTAAAAGGCGCTT
TAGATGCACGCGGTTCCAAGTTAATGCCAGATAAAAAGGATTTCGGTTATAGTTTTCCG
TGCGATGGTCCAGGCCGAGGAGGTACTTGTGATATTTCGGCATGGGACGCATTTTATTT
GGCAGTTTTTTGGATGTTAAATACTATTGGGTGGGTTACTTTTTATTGGCATTGGAAGCA
CATCACATTATGGCAAGGTAACGTTTCACAGTTTAATGAATCATCTACTTATTTGATGGG
CTGGTTAAGAGATTATTTATGGTTAAACTCTTCACAACTTATCAATGGATATAACCCTTTT
GGTATGAATAGTTTATCGGTCTGGGCATGGATGTTCTTATTTGGTCATCTTGTTTGGGCT
ACTGGATTTATGTTCTTAATTTCCTGGCGTGGATATTGGCAGGAATTGATTGAAACTTTA
GCGTGGGCTCATGAACGCACACCTTTGGCCAATTTAATTCGCTGGAGAGATAAACCGG
TGGCCCTTTCCATTGTACAAGCAAGATTGGTTGGATTGGCCCACTTTTCAGTAGGTTAT
ATATTCACTTATGCAGCTTTCTTGATTGCCTCTACATCGGGCAAATTTGGTTAA

>psaC
ATGTCACATTCAGTAAAGATTTATGATACATGTATAGGATGTACTCAATGTGTCCGGGCT
TGTCCTACTGATGTATTAGAAATGATACCTTGGGACGGATGTAAAGCAAAACAAATTGC
TTCTGCTCCAAGAACAGAGGACTGTGTTGGTTGTAAGAGATGTGAATCCGCCTGCCCA
ACGGATTTCTTGAGTGTTCGAGTTTATTTAGGATCTGAAACAACTCGCAGTATGGGTCT
AGCTTATTGA

>psaI
ATGACAGATTTCAACCTTCCCTCTATTTTTGTGCCTTTAGTAGGCCTAGTATTTCCGGTA
ATTTCAATGACTTCTTTATTTCTTTATGTTCAAAAAAACGAGATTGTTTAG

>psaJ
ATGCGAGATCTAAAAACATATCTCTCCGTGGCACCAGTTCTAAGCACGTTATGGTTTGG
AGCTTTAGCGGGTTTATTGATAGAGATTAATCGCTTTTTTCCGGATGCGTTGACATTCCC
CTTTTTTTCATTCTAG

>psbA
ATGACTGCAATTTTAGAGAGACGCGAAAGCGAAAGCCTATGGGGTCGCTTCTGTAACT
GGATAACCAGCACTGAAAACCGCCTTTACATTGGATGGTTTGGTGTTTTGATGATCCCG
ACCTTATTGACCGCAACTTCTGTATTTATTATCGCCTTCATTGCTGCCCCTCCAGTAGATA
TTGATGGTATTCGTGAACCTGTTTCTGGATCTCTACTTTACGGAAACAATATTATTTCTG
GTGCCATTATTCCTACTTCTGCAGCTATAGGTTTGCATTTTTACCCAATCTGGGAAGCAG
CATCTGTTGATGAATGGTTATACAACGGTGGTCCTTATGAACTAATTGTTCTACATTTCTT
ACTTGGTGTAGCTTGTTACATGGGTCGTGAGTGGGAGCTTAGTTTCCGTCTGGGTATGC
GACCTTGGATTGCTGTTGCATATTCAGCTCCTGTTGCAGCTGCTACCGCAGTTTTCTTG
ATTTATCCAATTGGTCAAGGAAGTTTTTCTGACGGTATGCCTCTAGGAATCTCTGGTACT
TTCAACTTTATGATTGTATTCCAGGCTGAGCACAACATCCTTATGCACCCATTTCACATG

TTAGGCGTAGCTGGGGTATTCGGCGGCTCCCTATTTAGTGCTATGCATGGTTCCTTGGTA
ACCTCTAGTTTGATCAGGGAAACCACAGAAAATGAATCTGCTAATGAAGGTTACAGAT
TTGGTCAAGAGGAAGAAACTTATAATATCGTAGCTGCTCATGGTTATTTTGGCCGATTG
ATCTTCCAATATGCTAGTTTCAACAACTCTCGTTCATTACACTTCTTCCTAGCTGCTTGG
CCTGTAGTAGGTATCTGGTTTACTGCTTTAGGTATCAGCACTATGGCTTTCAACCTAAAT
GGTTTTAATTTCAACCAATCCGTGGTTGATAGTCAAGGCCGTGTAATTAATACTTGGGCT
GATATCATCAACCGTGCTAACCTTGGTATGGAAGTTATGCATGAACGTAATGCTCATAAC
TTCCCTCTAGACTTAGCTGCTATCGAAGCTCCATCTACAAATGGATAA

>psbB
ATGGGTTTACCTTGGTATCGTGTTCATACCGTTGTATTGAATGATCCTGGTAGGTTGATTT
CTGTTCATATAATGCATACAGCTCTGGTTGCTGGTTGGGCCGGTTCGATGGCTCTGTATG
AATTAGCAGTTTTTGATCCCTCTGACCCTGTTCTTGATCCAATGTGGAGACAGGGCATG
TTCGTTATACCCTTCATGACTCGCTTGGGAATAACCAATTCATGGGGCGGTTGGAGTATC
ACAGGAGGGACTGTAACGAATCCAGGTATTTGGAGTTACGAAGGTGTAGCTGGAGCAC
ATATTGTTTTTTCCGGCTTATGCTTTTTGGCAGCTATCTGGCATTGGGTATATTGGGATCT
AGAAATATTTACCGATGAACGTACAGGAAAACCCTCTTTGGATTTGCCCAAGATCTTTG
GAATTCATTTATTTCTTTCAGGGGTGGCTTGCTTTGGTTTTGGTGCATTTCATGTAACAG
GCTTGTACGGTCCTGGAATATGGGTGTCCGATCCTTATGGACTAACCGGAAAAGTCCAA
CCTGTAAATCCATCGTGGGGCGTGGAAGGTTTTGATCCTTTTGTTCCAGGAGGAATAGC
TTCTCATCATATTGCAGCAGGGACTTTGGGTATATTGGCGGGTTTATTCCATCTTAGCGT
CCGCCCACCACAACGTCTATACAAAGGGTTGCGCATGGGAAATATTGAAACTGTCCTTT
CCAGTAGTATCGCTGCTGTTTTTTTCGCAGCTTTTGTTGTTGCTGGAACCATGTGGTATG
GCTCAGCAACTACTCCCATCGAATTGTTTGGTCCTACCCGTTATCAATGGGATCAGGGG
TACTTCCAGCAAGAGATATATCGAAGAGTCAGTGCCGGGCTAACAGAAAATAAAAATT
TATCAGAAGCCTGGTCTAAAATTCCTGAAAAATTAGCTTTTTATGATTACATCGGCAATA
ATCCGGCAAAAGGAGGATTATTCAGAGCTGGCTCAATGGATAACGGGGATGGAATAGC
GGTTGGGTGGTTAGGACACCCAATCTTTAGAGACAAAGAAGGGCATGAACTTTTTGTA
CGCCGTATGCCTACTTTTTTTGAAACATTTCCGGTCGTTTTGGTAGACGGCGATGGAATT
GTTAGAGCCGATGTTCCTTTTAGAAGGGCCGAGTCGAAGTATAGTGTTGAACAAGTGG
GTGTAACTGTTGAATTCTACGGTGGTGAATTCAATGGAGTTAGTTATAGTGATCCTGCTA
CTGTGAAAAAATATGCTCGGCGTGCTCAATTGGGTGAAATTTTTGAATTAGATCGTGCT
ACTTTGAAATCTGATGGTGTTTTTCGAAGCAGTCCAAGGGGTTGGTTTACTTTTGGACA
TGCTTCATTTGCTTTGCTTTTCTTCTTCGGACACATTTGGCACGGTGCTAGAACCTTGTT
CAGAGATGTTTTTGCTGGTATTGACCCAGATTTGGATGCTCAAGTTGAATTTGGAGCAT
TCCAGAAACTTGGAGATCCAACTACAAGAAGACAGGTAGTCTGA

>psbC
ATGAAAACCTTATATTCCCTGAGGAGGTTCTACCACGTGGAAACGCTCTTTAATGGAAC
TTTAGCTTTAGCTGGCCGTGACCAAGAAACCACCGGTTTCGCTTGGTGGGCCGGGAAT
GCCCGACTTATTAATTTATCCGGTAAATTACTAGGGGCTCATGTGGCCCATGCTGGATTA
ATCGTATTCTGGGCCGGAGCAATGAACCTATTTGAAGTGGCCCATTTCGTACCAGAGAA
ACCTATGTATGAACAAGGATTAATTTTACTTCCTCATCTAGCTACTCTAGGCTGGGGGT
AGGTCCGGGGGGGGAAGTTATAGACACTTTTCCATACTTTGTATCTGGAGTACTTCATT

TAATTTCCTCCGCAGTATTGGGCTTTGGCGGTATTTATCATGCACTTCTGGGACCTGAAA
CGCTTGAAGAATCCTTTCCATTCTTTGGTTATGTATGGAAAGATAGAAATAAAATGACC
ACAATTTTAGGTATTCACTTAATCTTGTTAGGTCTAGGTGCTTTTCTTCTAGTATTAAAGG
CTCTTTATTTTGGGGGCGTATATGATACTTGGGCTCCCGGAGGGGGAGATGTAAGAAAA
ATTACCAACTTGACCCTTAGTCCAAGTATTATATTTGGTTATTTACTAAAATCTCCCTTTG
GAGGGGAGGGGTGGATTGTTAGTGTGGACGATTTAGAAGATATAATCGGAGGTCATGT
ATGGTTAGGTTCCATTTGTGTACTTGGTGGAATCTGGCATATCTTAACCAAACCCTTCGC
ATGGGCTCGACGCGCATTTGTATGGTCTGGAGAAGCTTACTTATCTTATAGTTTAGCGGC
TTTATCCGTTTTTGGTTTCATTGCTTGTTGTTTTGTTTGGTTCAACAATACCGCTTATCCT
AGTGAGTTTTACGGACCCACTGGACCAGAAGCTTCTCAAGCCCAAGCATTTACTTTTC
TAGTTAGAGACCAACGCCTTGGGGCTAACGTGGGATCCGCTCAAGGACCTACTGGTTT
AGGTAAATATTTAATGCGTTCCCCAACCGGAGAAGTCATTTTTGGAGGAGAAACTATGC
GTTTTTGGGATCTGCGTGCTCCTTGGTTAGAACCTCTAAGGGGTCCAAATGGGTTGGAC
TTGAGTAGGTTGAAAAAAGATATACAACCTTGGCAAGAACGGCGTTCCGCAGAATATA
TGACTCACGCTCCTTTAGGTTCTTTAAATTCCGTGGGTGGCGTAGCTACCGAAATCAAT
GCAGTCAATTATGTCTCTCCTAGAAGTTGGTTATCTACCTCTCATTTTGTTCTAGGATTCT
TCTTCTTCGTAGGTCATTTGTGGCATGCGGGAAGAGCTCGCGCAGCTGCAGCAGGGTT
TGAAAAAGGAATTGATCGTGATTTTGAACCTGTTCTTTCCATGACCCCTCTTAATTGA

>psbD
ATGACTATAGCCCTTGGTAAATTTACCAAAGACCAAAATGATTTATTTGATATTATGGAT
GACTGGTTGCGGAGGGACCGTTTCGTTTTTGTAGGCTGGTCCGGTCTATTACTCTTTCC
TTGTGCCTATTTCGCTGTAGGGGGTTGGTTCACAGGTACAACCTTTGTAACTTCATGGT
ATACCCATGGATTGGCTAGTTCCTATTTGGAAGGATGCAATTTCTTAACCGCCGCAGTTT
CTACTCCTGCTAATAGTTTAGCGCATTCTTTGTTGTTACTATGGGGTCCTGAAGCACAAG
GAGATTTTACTCGTTGGTGTCAATTGGGGGGTCTATGGACTTTTGTTGCTCTCCATGGC
GCTTTCGGACTAATAGGTTTCATGTTACGTCAATTCGAGCTTGCTCGATCTGTTCAATTG
CGACCTTATAATGCAATCGCATTCTCTGGTCCAATTGCTGTTTTTGTTTCTGTATTTCTTA
TTTATCCGCTAGGGCAGTCTGGTTGGTTTTTTGCACCTAGTTTTGGCGTAGCAGCTATAT
TTCGATTCATCCTCTTTTTTCAAGGGTTTCATAATTGGACGTTGAACCCATTTCATATGAT
GGGAGTTGCCGGTGTATTGGGCGCTGCTTTGCTATGCGCTATCCACGGTGCTACTGTAG
AAAATACTTTATTTGAAGATGGTGATGGTGCAAATACATTCCGTGCTTTTAACCCAACTC
AGGCTGAAGAAACTTATTCAATGGTCACTGCTAACCGCTTTTGGTCTCAAATCTTTGGG
GTTGCTTTTTCCAATAAACGTTGGTTACATTTCTTTATGTTATTTGTACCAGTAACCGGTT
TATGGATGAGTGCTCTTGGAGTAGTCGGTCTGGCGTTGAACCTACGTGCCTATGACTTC
GTTTCTCAGGAAATTCGCGCAGCGGAAGATCCTGAATTTGAGACTTTCTACACCAAAA
ATATTCTCTTAAACGAAGGTATTCGTGCTTGGATGGCGGCTCAAGATCAGCCTCATGAA
AACCTTATATTCCCTGAGGAGGTTCTACCACGTGGAAACGCTCTTTAA

>psbE
ATGTCTGGAAGCACAGGAGAACGTTCTTTTGCTGATATTATTACCAGTATTCGATACTGG
GTCATTCATAGCATTACTATACCTTCCCTATTCATTGCAGGTTGGTTATTCGTCAGCACTG
GTTTAGCTTACGATGTATTTGGAAGCCCTCGACCAAACGAGTATTTCACAGAGAGCCG

ACAAGGTATTCCATTAATAACTGGCCGTTTTGATTCTTTGGAGCAACTCGATGAATTTAG
TAGATCGTTTTAG

>psbF
ATGACTATAGATCGAACCTATCCCATTTTTACCGTACGATGGTTGGCTGTTCACGGCCTA
GCTGTACCTACCGTCTTTTTTTTGGGGTCAATATCAGCAATGCAGTTCATCCAACGATAA

>psbH
ATGAATACAATAGGATTTATGGCTACACAAACTGTTGAGAAGAGTTCTAGATCTGGTCG
TCCAAGACGAACTAATGTAGGGAGTTTATTAAAACCCTTGAATTCGGAGTATGGTAAAG
TAGCTCCTGGGTGGGGAACGACTCCATTGATGGGGGTTGCAATGGCTCTATTTACGGTA
TTTCTATCTATTATTTTGGAGATTTATAATTCTTCCGTTTTATTGGATGGAATTTTAATGAA
TTAG

>psbI
ATGCTTACTCTTAAACTTTTCGTTTACACAGTAGTAATATTTTTTGTTTCTCTCTTCATCT
TTGGATTCCTATCTAATGATCCAGGACGTAATCCTGGACGTGAAGAATAA

>psbJ
ATGGCCGATACTACGGGAAGGATTCCTCTTTGGATAATAGGTACTGTAGCTGGTATTCTT
GTGATTGGTTTAATAGGTATTTTCTTTTATGGTTCATATTCCGGATTGGGTTCATCCCTGT
AG

>psbK
ATGCTTAATATCTTTAGTTTGATCTGTCTTAATTCTGCCCTTTATTCGAGTAGTTTTTTCTT
AGGCAAATTGCCTGAGGCCTATGCTTTTTTGAATCCAATCGTAGATTTTATGCCAGTCAT
ACCTTTGTTTTTTTTTCTCTTAGCCTTTGTTTGGCAAGCTGCTGTAAGTTTTCGATAA

>psbL
ATGACACAATCAAACCCAAACGAACAAAATGTTGAATTGAATCGTACCAGTCTCTACT
GGGGGTTATTACTCATTTTTGTACTTGCTGTTTTATTTTCCAATTATTTCTTCAATTAA

>psbM
ATGGAAGTTAATATTCTTGCCTTTATTGCTACTGCACTATTCATTCTAGTTCCTACCGCTT
TTCTACTTATCATTTATGTAAAAACAGTTAGTCAAAATAATTAG

>psbN
ATGGAAACAGCAACCCTAGTCGCCATCTTTATATCTGGTTTACTTGTAAGTTTTACTGGG
TACGCCTTATATACCGCTTTTGGGCAACCTTCTCAACAACTAAGAGATCCATTCGAGGA
ACATGGAGACTAG

>psbT
ATGGAAGCATTGGTTTATACATTTCTCTTAGTCTCGACTCTAGGAATCATTTTTTTCGCTA
TTTTTTTTCGAGAACCGCCTACAATTTCATCTAAAAAGATGAAATAA

>psbZ
ATGACTCTTGCTTTCCAATTGGCCGTTTTTGCATTAATTGCTACTTCATTAATCTTATTAA
TTAGCGTACCCGTTGTATTTGCTTCTCCTGATGGTTGGTCAAGTAATAAAAATGTTGTAT
TTTCCGGTACATCATTATGGGTTGGTTTAGTCTTTCTAGTAGGTATCCTTAATTCTCTTAT
CTCTTGA

>rbcL
ATGTCACCACAAACAGAGACTAAAGCAAGTGTTGGATTCAAAGCTGGTGTTAAAGAG
TACAAATTGACTTATTATACTCCTGAATACGAAACCAAGGATACTGATATCTTGGCAGCA
TTTCGAGTAACTCCTCAACCTGGAGTTCCTCCAGAAGAAGCAGGGGCCGCAGTAGCTG
CCGAATCTTCTACTGGTACATGGACAACTGTGTGGACCGATGGACTTACCAGTCTTGAT
CGTTACAAAGGGCGATGCTACCACATCGATGCCGTTCCCGGAGAAGAAAATCAATATAT
ATGTTATGTAGCTTATCCTTTAGACCTTTTTGAAGAAGGTTCTGTTACTAACATGTTTACT
TCCATCGTAGGTAATGTATTTGGGTTCAAAGCCCTGCGCGCTCTACGTCTGGAAGATCT
GCGAATCCCTCCTGCTTATGTTAAAACTTTCCAAGGCCCACCTCATGGGATCCAAGTTG
AAAGAGATAAATTGAACAAGTACGGCCGTCCTTTGTTGGGATGTACTATTAAACCTAAA
TTGGGCTTATCCGCTAAAAACTACGGTAGAGCAGTTTATGAATGTCTTCGCGGTGGGCT
TGATTTTACCAAAGATGATGAAAACGTGAACTCCCAACCGTTTATGCGTTGGAGAGAC
CGTTTCTTATTTTGTGCCGAAGCTATTTATAAAGCACAGGCTGAAACAGGTGAAATCAA
AGGACATTACTTGAATGCTACTGCAGGTAACTGCGAAGAAATGATCAAAAGAGCTGTA
TTTGCTAGAGAATTGGGAGTCCCTATCGTCATGCACGACTACCTAACAGGGGGATTCAC
TGCAAATACTACCTTGGCTAGTTATTGCCGAGATAATGGTCTACTTCTTCACATTCACCG
TGCAATGCATGCGGTTATTGATAGACAGAAGAATCATGGTATGCACTTCCGTGTACTAG
CTAAAGCGTTACGTATGTCTGGTGGAGATCATATTCACTCTGGTACCGTAGTAGGTAAA
CTTGAAGGGGAAAGAAACATCACTTTGGGTTTTGTTGATTTATTGCGTGATGATTTTATT
GAAAAAGATCGAACTCGCGGTATCTATTTCACTCAAGATTGGGTCTCTCTACCAGGTGT
GTTGCCTGTAGCTTCAGGAGGTATTCACGTTTGGCATATGCCGGCTTTGGTTGAAATCT
TTGGAGACGATTCCGTACTACAGTTCGGTGGAGGAACTTTAGGACACCCTTGGGGTAA
TGCGCCAGGTGCCGTAGCTAACCGAGTAGTTCTAGAAGCATGTGTACAAGCCCGTAAT
GAAGGGCGTGATCTTGCTCGTGAGGGTGGTGATATTGTTCGTGAGGCTACCAAATGGA
GTCCTGAACTAGCTGCAGCTTGTGAGGTATGGAAGGAGATCACATTTAATTTCGAGGA
AGTGGATAAATTGGATTATTGA

>rpl14
ATGATTCAACCTCAGACCCATTTAAATGTAGCGGATAACAGCGGGGCTCGAGAATTGAT
GTGTATTCGAATCATAGGAGCTAGCAATCGCCGATATGCTCATATCGGCGACGTTATTGT
TGCTGTGATCAAAGAAGCAGTGCCAAACATGCCTCTAGAAAGATCAGAAGTAGTCAG
AGCTGTAATTGTACGTACTTGTAAAGAACTCAAACGTGATAACGGTATGATAATACGAT
ATGACGACAATGCTGCAGTTGTTATTGATCAAGAAGGAAATCCAAAAGGAACTCGAAT
TTTTGGTGCCATCGCTCGGGAATTGAGACAATTAAATTTTACTAAAATAGTTTCATTGGC
TCCTGAGGTATTATAA

>rpl16

ATGCTTAGTCCCAAAAGAACCAGATTCCGCAAACAACATAGAGGAAGAATGAAAGGA
ATATCTTATCGAGGTAATCATATTTGTTTCGGTAAATATGCTCTTCAGGCACTTGAACCC
GCTTGGATCTCATCTAGACAAATAGAAGCAGGCCGACGAGCAATGACACGAAATGCGC
GTCGTGGTGGAAAATTATGGGTACGCATTTTTCCAGACAAACCAGTTACACTAAGACC
CGCGGAAACCCGGATGGGTTCCGGAAAAGGATCCCCTGAATATTGGGTAGCTGTTGTT
AAACCCGGTCGAATACTTTATGAAATGAGTGGAGTAACAGAAAATATAGCTAGAAGGG
CTATTTCAATAGCAGCATCTAAAATGCCTATACGAACTCAATTCATTACGGGATAA

>rpl20
ATGACTAGAATTAAACGGGGATATGTAGCTCGGAGACGTAGAACAAAAATTCGTTTATT
TGCATCAAGCTTTCGAGGAGCTCATTCAAGACTTACTCGAACTATTACTCAACAGAAA
ATAAGAGCTTTGGTTTCGGCTCATCGGGATAGGAATAGGCAAAAGCGAAATTTTCGTCG
TTTGTGGATCACACGAATAAACGCAGTAATTCGCGAAAATAGAGTAACTTATAGTTATA
GTAGATTAATACACGATCTATATAAGAAACAGTTGCTTCTTAATCGTAAAATACTTGCAC
AAATAGCTATATTCAATAGGAATTCTCTTTACATGATTTCCAATGAGATCATAAACGAAG
TAGAATCCACCGGAATAATTTAA

>rpl22
ATGCTAAAGAAGAAAAAAACAGAGGTATATGCTTTAGGTCAACATATATCTATGTCTGC
TGACAAAGCACGAAGGGTAATTGATCAGATTCGTGGACGTTCCTACGAGGAAACACTT
ATGATACTAGAACTCATGCCTTATCGGGCATGCTATCCTATTTTAAAATTAGTTTATTCAG
CAGCTAATGCTAGTCACAATATGGGTTCTAATGAACCCAACTTTGTTATTAGCCAAGCC
GAAGTCAACGAGGGCAGCACTGTAAAGAAATTAAAACCTCGAGCTAGAGGACGTAGT
TTTCCGATAAAAAGATCAACCTGTCATATAACTATTGTAATGAAAGACATATCTTTAGAT
GATGAATATGTAGAGATAGATTCGTTAAAAAAACCTAAATGA

>rpl23_copy2
ATGGATGGAATCATATATGCAGTATTTACAGACAAAAGTATTCGGTTATTGGGGAAAAAT
CAATATACTTCTAATGTCGAATCAGGATCAACTAGGACAGAAATAAAGCATTGGGTCGA
ACTCTTCTTTGGTGTCAAGGTAATAGCTATGAATAGTCCTTCCGGGAAAGGGTAG

>rpl23
ATGGATGGAATCATATATGCAGTATTTACAGACAAAAGTATTCGGTTATTGGGGAAAAAT
CAATATACTTCTAATGTCGAATCAGGATCAACTAGGACAGAAATAAAGCATTGGGTCGA
ACTCTTCTTTGGTGTCAAGGTAATAGCTATGAATAGTCCTTCCGGGAAAGGGTAG

>rpl2_copy2
ATGGCGATACATTTATACAAAACTTCTACCCCGAGCACACGCAATGGAACCGGAAACA
GTCAAGTGAAATCCAATCCACGAAATAATTTGATCTACGGACAGCATCATTGTGGTAAA
GGTCGTAATGCCAGAGGAATCATTACCGCAAGGCATAGAGGGGGAGGTCATAAGCGTC
TATACCGTAAAATCGATTTTCGGCGGAATGAAAAAGACATATATGGTAGAATCGTAACC
ATAGAATACGACCCTAATCGAAATGCATCCATTTGTCTCATACACTATGGGGATGGTGAG
AAGAGATATATTTTACATCCCAGAGGGGCTAGAATTGGAGATACCATTGTTTCTGGTAC
AGAAGTTCCTATAAAAATGGGAAATGCCCTACCTTTGACCGATATGCCCTTAGGCACGG

CCATACATAACATAGAAATCACACTTGGAAAGGGTGGACAATTAGCTAGAGCAGCAGG
TGCTGTAGCGAAACTGATTGCAAAAGAGGGGAAATCGGCCACATTAAAATTACCTTCT
GGAGAGGTCCGTTTGATATCCAAAAACTGCTCAGCAACAGTCGGACAAGTGGGGAAT
GTTGGGGTGAACCAGAAAAGTTTGGGTAGAGCCGGATCTAAGCGTTGGCTAGGTAAG
CGTCCTGTAGTAAGAGGGGTAGTTATGAACCCTGTAGACCATCCCCATGGGGGTGGTG
AAGGGAGAGCCCCAATTGGTAGAAAAAAACCCACAACCCCTTGGGGTTATCCTGCACT
TGGAAGAAGAAGTCGAAAAAGGAAGAAATATAGTGAGAATTTGATTCTTCGTCGTCGT
AAATAG

>rpl2
ATGGCGATACATTTATACAAAACTTCTACCCCGAGCACACGCAATGGAACCGGAAACA
GTCAAGTGAAATCCAATCCACGAAATAATTTGATCTACGGACAGCATCATTGTGGTAAA
GGTCGTAATGCCAGAGGAATCATTACCGCAAGGCATAGAGGGGGAGGTCATAAGCGTC
TATACCGTAAAATCGATTTTCGGCGGAATGAAAAAGACATATATGGTAGAATCGTAACC
ATAGAATACGACCCTAATCGAAATGCATCCATTTGTCTCATACACTATGGGGATGGTGAG
AAGAGATATATTTTACATCCCAGAGGGGCTAGAATTGGAGATACCATTGTTTCTGGTAC
AGAAGTTCCTATAAAAATGGGAAATGCCCTACCTTTGACCGATATGCCCTTAGGCACGG
CCATACATAACATAGAAATCACACTTGGAAAGGGTGGACAATTAGCTAGAGCAGCAGG
TGCTGTAGCGAAACTGATTGCAAAAGAGGGGAAATCGGCCACATTAAAATTACCTTCT
GGAGAGGTCCGTTTGATATCCAAAAACTGCTCAGCAACAGTCGGACAAGTGGGGAAT
GTTGGGGTGAACCAGAAAAGTTTGGGTAGAGCCGGATCTAAGCGTTGGCTAGGTAAG
CGTCCTGTAGTAAGAGGGGTAGTTATGAACCCTGTAGACCATCCCCATGGGGGTGGTG
AAGGGAGAGCCCCAATTGGTAGAAAAAAACCCACAACCCCTTGGGGTTATCCTGCACT
TGGAAGAAGAAGTCGAAAAAGGAAGAAATATAGTGAGAATTTGATTCTTCGTCGTCGT
AAATAG

>rpl32
ATGGCAGTTCCAAAAAAGCGTACTTCTATATCAAAAAAGCGTATTCGTAAAAATATTTG
GAAAGGGAGGGGATATTGGACAGCGTTAAAAGCTTTTTCGTTAGGAAAGTCTCTTTCT
ACAGGAAAGTCAAAAAGTTTTGTTGTGCGGCAAACAACTCAATAA

>rpl33
ATGGCCAAGGGTAAAGATGTCCGACTAAGGGTAATTTTGGAATGTACCGCTTGTGTCCG
AAACGGTGCTAATAAGAGTAAGAGATCGATGGGCATTTCCAGATATATTACTCAAAAGA
ACCGACAGAATATGCCTAATCGATTAGAATTGAGAAAATTCTGTCGCTATTGTTCCAAA
CATACCATTCATGGGGAGATAAAAAAATAG

>rpl36
ATGAAAATAAGAGCTTCTGTTCGTAAAATTTGTGAAAAATGTCGATTAATCCGGAGACG
CGGACGAATTATAGTAATTTGTTCCAACCCGAAACATAAACAAAGACAGGGATAA

>rpoA
ATGGTTCGAGAGAAAGTAACAGTATCTACTCGGACACTACAGTGGAAGTGTGTTGAAT
CAAGAACAGACAGTAAACGTCTTTATTATGGACGCTTTATCCTGTCTCCACTTATGAAA

GGTCAAGCCGACACAATAGGCATTGCGATGCGAAGAGCTTTGCTTGGAGAAATAGAAG
GAACGTGTATTACACGTGTAAAATTGGAATCTGATAAAATCCTACATGAATATCATGAAT
ATTCTACCATAGGGGGTATTCAAGAATCGGTACATGAAATTTTCATGAATTTGAAAGAA
ATTGTATTGAGAAGTAATATATATGGAACTTGTCACGCGTCTATTTGTGTCAAGGGTCCT
GGATATGTAACTGCCCAAGACATCATCCTACCGACTTATGTAGAAATCGTTGATAATACA
CAACATATAGCTAGCTTGACGGAACCAATTGATTTGTGTATTGGATTACAAATTGAGAA
AAATCGAGGATATCTTATAAAAACGCCGCATCACTTTCAAGATGGCAGTTATCCTATAGA
TGCCGTATTCCTGCCTGTTCGAAATGCGAATCATAGTATTCATTCCTACGGGAATGGGAA
TGAAAAACAAGAGATACTTTTTCTCGAAATATGGACAAATGGAAGTTTAACTCCGAAA
GAAGCACTTCATGAAGCCTCTCGGAATTTGATTGATTTATTTATTCCTTTTTTACATATGG
AAGAATAA

>rpoB
ATGCTCGGCAATGGAAATGAGGGGATGTCTACAATACCTGGATTTAATCAGATCCAATT
TGAAGGATTTTGTAGGTTCATTGATCAGGGTTTGATGGAAGAACTTTATAAGTTTCCAA
AAATTGAAGATACAGATCAAGAAATCGAATTTCAATTATTTGTGCAAACATATCAATTG
GTAGAACCTTTGATAAAGGAAAGAGATGCTGTGTATGAATCACTCACATATTCTTCTGA
ATTATATATATCTGCTGGATTAATTTGGAAAACCGGCAGGGATATGCAAGAACAAACCAT
TTTTATTGGAAATATTCCTCTAATGAATTCCTTGGGAACTTTTGTAGTAAATGGAATATAT
AGAATTGTGATCAATCAAATATTGCAAAGCCCCGGTATTTATTACCGATCAGAATTGGAC
CATAACGGAATTTCGGTCTATACCGGTACCATAATATCAGATTGGGGAGGAAGATCAGA
ATTAGAGATTGATAGAAAAGCAAGGATATGGGCTCGTGTGAGTAGGAAACAAAAAATA
TCTATTCTAGTTCTATTATCAGCTATGGGTTCGAATCTAAGAGAAATTCTAGAGAATGTT
TGTTATCCTGAAATTTTTTTGTCTTTTCTGAATGATAAGGAGAGAAAAAAAATTGGGTC
AAAAGAAAATGCCATTTTGGAGTTTTATCAACAATTTGCTTGTGTAGGTGGGGATCCGG
TTTTTTCTGAATCCTTATATAAGGAATTACAAAAGAAATTCTTTCAACAAAGATGTGAAT
TAGGAAAGATTGGTCGGCGAAATATGAATCGAAGACTGAACCTTGATATACCTCAAAA
CAACACATTTTTGTTACCACGAGATATATTGGCGGCTGCCGATCATTTGATCGGACTTAA
ATTTGGAATGGGTACACTTGACGATATGAATCATTTGAAAAATAAACGTATTCGTTCTGT
AGCCGACCTTTTACAAGATCAATTCGGGTTGGCTCTGGCTCGTTTAGAAAATGTGGTTC
GAGGAACTATATGCGGAGCAATTCGACATAAATTGGTACCGACTCCTCAGAATTTGGTA
ACTTCAACTCCATTAACAACTACTTATGAGTCTTTTTTCGGGTTACACCCACTATCTCAA
GTTTTGGATCGAACGAATCCATTGACACAAATAGTTCATGGAAGAAAATTGAGTTATTT
GGGACCTGGAGGACTGACAGGGCGAACTGCGAGTTTTCGAATACGAGATATCCATCCT
AGTCACTATGGGCGTATTTGCCCAATTGACACATCTGAGGGAATCAATGTTGGACTTAT
TGGATCCTTAGCAATTCATGCGAGGATTGGTCATTGGGGATCTCTAGAAAGCCCCTTTT
ATGAAATTTCTGAGAGATCAACAGGGGGGGTACGGATGCTTTATTTATCACCAGGTAGA
GATGAATACTATATGGTAGCAGCAGGAAATTCTTTGGCTTTGAATCATAATATTCAGGAA
GAACAGGTTGTTCCAGCTCGATACCGTCAAGAATTCTTAACTATTGCATGGGAACAGGT
TCATTTTCGAAGTATTTATCCCTTTCAATATTTTTCGATTGGAGCTTCCCTCATCCCTTTT
ATCGAACATAATGATGCGAATCGAGCTTTAATGAGTTCTAATATGCAACGTCAAGCAGT
TACTCTTTCTCAGTCCGAGAAATGTATTGTTGGAACTGGGTTAGAACGACCGGCAGCTC
TAGATTCAGGGGCTCTTGCTATAGCCGAAGTCGAGGGAAGGGTCATTTCTACTGATACT
GACAAGGTCCTTTTATTAGGTAATGGAGATACTCTAAGCATTCCATTAGTTATTTTTCAA

CGTTCCAACAAAAATACTTGTATGCATCAAAAACCCCAGGTTCGGCGAGGTAAATGCA
TTAAAAAAGGACAAATTTTAGCGGATGGTGCTGCTACGGTTGGCGGCGAACTTGCTTT
GGGGAAAAACGTATTAGTAGCTTATATGCCGTGGGAAGGTTACAATTCTGAAGATGCAG
TACTCATTAGCGAGCGTTTGGTATATGAAGATATTTATACTTCTTTTCACATACGGAAATA
TGAAATTCAGACTCATGTGACAAACCAAGGCCCTGAAAGGATCACTAATGAAATACCG
CATTTAGAGGCTCATTTATTACGCAATTTAGACAAAAAAGGAATTGTGATGCTGGGATC
TTGGGTAGAGGCGGGTGATATTTTAGTAGGTAAATTAACACCCCAGGTGGTGAAAGAA
TCGTCGTATGCCCCCGAAGATAGATTGTTACGAGCCATACTTGGCATTCAGGTATCTACT
TCAAAAGAAACTTGTCTAAAACTACCTATAGGTGCTCGGGGTCGGGTTATTGATGTGAG
GTGGATTCAGAAAAGGGGGGGTTCTAGTTATAATCCAGAAACGATTCGCGTATATATTT
CACAGAAACGTGAAATCAAAGTAGGCGATAAAGTAGCTGGAAGACACGGAAATAAGG
GTATCATTTCAAAAATATTGCCTAGACAAGATATGCCTTATTTGCAAGATGGAAGACCTG
TTGATATGGTCTTCAACCCGTTAGGAGTCCCTTCACGAATGAATGTAGGACAGATATTT
GAATGTTCGCTCGGGTTAGCGGGGAGTCTACTAGACAGACATTATCGAATAGCACCTTT
TGATGAGAGATATGAACAAGAAGCTTCAAGAAAACTAGTGTTTTCTGAATTATATGAAG
CTAGTAAGCAAACAGCGAATCCATGGGTATTTGAACCTGAGTATCCAGGAAAAAGCAG
AATCTTTGATGGAAGGACGGGGAATCCTTTTGAACAACCCGTTATAATAGGAAAGCCTT
ATATCTTGAAATTAATCCATCAAGTTGATGATAAAATCCACGGACGTTCCAGCGGGCATT
ATGCCCTTGTTACACAACAACCTCTTAGAGGAAGGGCCAAACAAGGGGGGCAACGGG
TAGGAGAAATGGAGGTTTGGGCTCTAGAAGGATTTGGTGTTGCTCATATTTTACAAGAG
ATGCTTACTTATAAATCTGATCATATTAGAGCCCGCCAGGAAGTACTTGGTACTACGATC
ATTGGAGGAACAATACCTAATCCTGAGGATGCTCCAGAATCTTTTCGATTACTCGTTCG
AGAACTACGATCTTTAGCTCTGGAACTGAACCATTTCCTTGTATCTGAGAAGAACTTCC
AGATTAATAGGAAGGAAGCTTAA

>rpoC1
ATGATCGATCGGTATAAACATCAACAGCTCAGGATTGGATCAGTTTCTCCTCAACAAAT
AAGTGCTTGGGCCACAAAAAATTTTACCTAATGGAGAGATAGTTGGAGAGGTGACAAAA
CCCTACACTTTTCATTACAAAACCAATAAACCGGAAAAAGATGGATTATTTTGTGAAAG
AATTTTTGGACCTATAAAAAGTGGAATTTGTGCTTGTGGAAATTATCGAGTAATCGGAG
ATGAAAAAGAAGACCCGAAATTTTGTGAACAATGCGGAGTTGGATTTGTTGATTCTCG
AATACGAAGGTATCAAATGGGCTACATCAAACTCGCATGCCCCGTAACCCATGTGTGGT
ATTTAAAACGCCTTCCTAGTTATATTGCGAATCTTTTAGATAAACCTCTTAAAGAATTGG
AGGGACTAGTATACTGCGATTTTTCTTTTGCTAGGCCCATAACTAAAAAACCTACTTTCT
TACGATTACGAGGTTCATTCGAATATGAAATCCAATCTTGGAAATACAGCATTCCACTTT
TTTTTACTACCCAGGGCTTCGATACATTTAGAAATCGAGAAATCTCTACTGGGGCAGGC
GCTATACGAGAACAATTAGCTGATCTAGATTTACAAATTATTATAGATAAGTCGTTGGTA
GAATGGAAAGAATTACGGGAAGGAGGGCCCACAGGGAATGAATGGGAAGATCGAAAA
GTTGGAAGAAGAAAGGATTTTTTGGTTAGACGCATGGAATTGGCTAAGCATTTTATTCG
AACAAATATAGAACCGGAATGGATGGTTTTGTGTCTATTACCAGTTCTTCCTCCTGAGTT
GAGACCGATCATTCAGATCGATGGGGGTAAACTAATGAGTTCGGATATTAACGAACTCT
ATAGAAGAGTTATCTATCGGAACAATACTCTTACCGATCTATTAACAACAAGTAGATCTA
CGCCAGAAGAGCTAGTAATGTGTCAGGAGAAATTAGTACAAGAAGCCGTGGATACACT
TCTTGATAATGGAATCCGTGGACAACCCATGAGGGACGGTCATAATAAAGTTTACAAAT

CTTTTTCAGATGTAATTGAAGGCAAAGAGGGAAGATTTCGTGAGACTCTGCTTGGTAA
ACGGGTGGATTATTCAGGGCGTTCCGTCATTGTCGTGGGCCCCTCACTTTCATTACATC
GATGTGGATTGCCTCGCGAAATAGCAATAGAGCTTTTCCAGACATTTGTAATTCGGGGT
CTAATTAGACAACATCTTGCTTCGAACATAGGAGTTGCTAAGAATAAAATTCGGGAAAA
AGAGCCCATTGTATGGGAAATACTTCAGGAAGTTATGCACGGGCATCCTGTATTGCTGA
ATAGAGCACCCACTCTGCATAGATTAGGTATACAGGCATTTCAGCCCGTTTTAGTGGAG
GGGCGCGCTATTTGTTTACACCCATTAGTTTGTAAGGGGTTCAATGCAGATTTTGATGG
GGATCAAATGGCTGTTCATGTGCCCTTATCTTTGGAGGCTCAAGCGGAGGCCCGTTTAC
TTATGTTTTCTCATATGAATCTTTTGTCTCCAGCTATTGGGGATCCCATTTCCGTACCAAC
TCAAGATATGCTTATTGGACTCTATGTATTAACGAGCGGAAATCGTCAAGGTATTTGTGT
AAATAGGTATAACCCATGGAATCGCAGAAACTATCAAAATAAAAAAAATGACAATAATA
ACTATAAGTATACGAAAGAGCCCTTTTTTTGTAATTCTTATGATGCAATTGGAGCTTATC
GGCAGAAACGAATCAATTTAGATAGTCCTTTGTGGCTCCGGTGGCGACTAGATCAGCG
CGTTATTGCTTCAAGAGAAACTCCCACCGAAGTCCATTATGAATCTTTAGGTACTTATTA
TGAGATTTATGGACACTATCTAATTGTAAGAAGTATAAAAAAAAAAATTATTTTATATAT
ATCCGAACCACTGTTGGTCATATTTCTCTTTATCGAGAAATCGAAGAAGCCATACAGGG
GTTTTCTCAAGCCTGTTCATATGGTATACCTAATTAA

>rpoC2
ATGGCAGAACGCGCCAATCTGGTCTTTCACAATAAAGTGATAGACGGAACTGTCATGA
AACGACTTATTAGTAGATTAATAGATCACTTCGGAATGGCATATACATCACACATCTTGG
ATCAAGTGAAAACCCTGGGTTTTCAACAAGCTACTGCTACATCCATTTCATTAGGAATT
GATGATCTTTTAACAATACCTTCTAAAGGATGGCTAGTCCAAGATGCTGAACAACAAAG
TTTTATTTTGGAAAAACATCACCATTATGGGAATGTACACGCGGTAGAAAAATTACGCC
AATCCATTGAAATATGGTATGCTACAAGTGAATATTTGCGACAAGAAATGAATCCTAATT
TTAGGATGACTGACCCTTTTAATCCAGTTCATATGATGTCTTTTTCGGGAGCTCGAGGA
AACGCATCTCAGGTACATCAATTAGTAGGTATGAGAGGATTAATGTCAGACCCTCAAGG
GCAAATGATTGATTTACCCATTCAAAGCAATTTACGCGAAGGGCTCTCTTTAACAGAAT
ATATAATTTCTTGCTACGGAGCCCGTAAAGGGGTCGTGGATACTGCTGTACGAACATCA
GACGCTGGATATCTCACGCGCAGACTTGTTGAAGTAGTTCAACACATTGTTGTACGTCG
AACAGATTGCGGCAGCGTCCGGGGTATTTCTGTTAGTCCTCGGAATGGGATGATGCCG
GAAAGGATTTTTACCGAAACATTAATTGGTCGTGTATTAGCGGATGATATATATATGGGC
TTGAGATGTATTGCCACTAGAAATCAAGACATTGGAATTGGACTTGTAAATAGATTTATA
ACTTTTCGAGCACAACCAATATCTATTCGAACTCCCTTTACTTGTAGGAGTACATCTTGG
ATTTGTCGATTATGTTATGGCCGGAGTCCAACTCATGGCGACCTGGTTGAATTGGGAGA
AGCTGTAGGTATTATTGCGGGCCAGTCAATTGGAGAACCGGGTACTCAATTAACATTAA
GAACTTTTCATACGGGTGGAGTATTCACAGGGGGTACTGCAGAACATGTCCGAGCCCC
TTCTAATGGAAAAATCAAATTTAATGAGGATTTGGTTCATCCGACACGTACACGTCATG
GACATCCCGCCTTTTTATGTTCTATAGACTTGTATGTAACTATTGAGAGTGAAGATATTAT
ACATAATGTGAATATTCCACAAAAAAGTTTTCTTTTAGTTCAAAATGATCAATATGTAGA
ATCCGAACAAGTGATTGCCGAAATTCGCGCGGGAACATCCACTTTGAATTTTAAAGAG
AAGGTTCGAAAACATATTTATTCTGACTCAGATGGCGAAATGCACTGGAGTACTGATGT
GTATCATGCACCAGAATTTATATACGGTAATGTTCATCTATTACCAAAAACAAGTCATTT
ATGGATATTATTGGGGGGACCATACAGATACAGTCTAGTCTCCCCCTCACTCCACAAGG

ATCAAGATCAAATAAACGCGCATTCTCTTTCTGTCAAGCGGGGGGAGACTTCTAATCTC
TCGGTAACTAATGATCCGGCGATACACAAATTCTTTAGTTCGGATTTTTCTGGTAAAGA
AGAAGGTAGAATTGCTGATTATTCAGACCTTAATCGAATCATATGTGTTGATTCTTGTAA
TCTAATATATTCACCCATTCTCCACGATAACTTAGATTTATTGTCAAAGAGACGAAGAAA
TAGATTCATCATTCCACTCCAACGGACTCAAGAGTGCGAGAACAAACTAATGTCCCGT
TCAGGTATCTCTATTGAAATCCCTACAAATGGCATTTTCCGTAGAAATAGTATTCTTGCT
TATTTCGACGATCCTCGATACAGAAGAAAAAGATCGGGAATTACTAAATATGGAACTAT
AGAAATGCATTCAATCTTCAAAAAAGAGGATTTGATTGAATATCGAGGAATAGGGGAAT
TTAGGCCAAAATACCAAATGAAAGTAGATCGGTTTTTTTCATTCCCGAGGAAGTCTAT
ATCTTTCCCGGATCTTCTACCATAATGGTACGTAACAACAGCCTGATTGGGGTAGAGAC
CCCAATCACTTTAAATATAAAAAGCCGGGTGGGCGGATTGGTCCGGATGGAGAAAAAA
AAAGGAATTGAACTAAAAATTTTTTCTGGAGATATCCATTTTCCGGGCGAGACAGATAA
GATATCCCGACATAGCGGCGTTTTGATACCACCAGGGGCGGGAAAAAAAAATTCCAAA
AAATCAAAAAAGTTGAAAAATGGGATCTATGTCCAACGGATCACACCTAGTAAGAAAA
AGTATTTTGTTTTTGTTCGACCTGTGGTTACATATGAAATAACGGACGGTATAAATTTATC
AACACTTTTCCCCCGGGATCTGTTGCAGGAAAGGGATGATGTGCAACTTCGAGTTGTT
AATTATATTCTTTATGGAAATGGCAAACCAATTCGAGGAATTTCTGATACAAGTATTCAA
TTAGTTCGGACTTGTTTAGTATTGAGCTGGGACCAAGAAAAAAAAAGTGCTTCTATCG
AAGAAGCCCGCGCTTCCTTTGTTGAAATAATGACAAATGGTTTGATTCGACATTTCTTA
AGAATTGACTTATTGAAATTTCCTATTTCGTATATCAGAAAAAGGAATGATCCGTCTGAT
TCAGGATTATTCTCTGATAATGGATCAGATTGTACCAATATCAATCCCTTTTCTTCCATCT
ATTCCAAAGCAAGAATTCAACAAAATCAAGGAACTATTCATACATTGTTGAATAGAAAT
AAGGAATGCCAATCGTTGATTCTTTTATCATCATCCAATAGTTTTCGAATGGGTCCGGTA
AAAAATCACAGTGTGATAAAAGAATCAATTCACAAAAATCCTCGAATTCCAATTAGGA
ATTCGTTGGGCCCCTTAGGAACAGCCTTTCCTATTGCGAATTGTTATTCATTTTATCATTT
ATTAACTCATAATCATATATTGATAACTAACTATTTACAACTTGACAATTTAAAACAAAC
GTTTCAAATAATTAAATATTATTTAATGGATGAACATGGGAAAATTTATAATCCCGACCTC
TCTAGTAATATTTTTTTGAATCCATTCCATTTGAATTGGTATTTTCTCGATCATAATTTTTA
TGAAAAGACATCTACAATAATGAGTCTTGGGCAGTTGATTTGTGAAAATGTATGTATAG
CCAAAAAAAGACCCCGCCTAAAATCGGGTCAAGTTATACTTGTTCAAGTTGACTCTATA
GTAATACGATCCGCAAAGCCTTTTTTGGCCACCCCGGGAGCAACTGTTCATGGCCATTA
TGGGGAAATCCTTTACGAAGGAGAAACTTTAGTTACATTTTTTTATGAAAAATCGAGAT
CTGGTGATATAACGCAAGGTCTTCCAAAAGTAGAACAGGTATTAGAAATGCGTTCGATC
GATTCAATATCGATGAATCTAGAAAAAAGGATTGCGGTTTGGAACGAATGTATAACAAG
AATTCTTGGAATTCCCTGGGGATTTTTGATTGGTGCTGAACTAACTATAGTGCAAAGCC
GTATCTCTTTGGTTAATAAGATACAAAAAGTTTATCGATCCCAGGGGGTGCGGATTCATA
ATAGGCATATAGAAATTATTGTACGTCAAATAACATCAAAGGTTTTGGTTTCAGAAGATG
GAATGTCTAATGTTTTTTCACCCGGAGAACTAATTGGATTGTTACGAGCGGAACGAATG
GGGCTCGCTTTAGAAGAAGCGATCTGTTACCGAGCCATCTTATTGGGAATAACAAGAG
CATCGCTCAATACTCAAAGTTTCATATCTGAGGCGAGTTTTCAAGAAACTGCTCGGGTT
TTAGCAAGGGCGGCTCTCCGGGGCCGTATTGATTGGTTGAAGGGTCTGAAAGAGAACG
TTGTTTTGGGGGGGATGGTACCTGTTGGTACCGGATTCAAAGGATTAGTATACCCTTCA
ACTTCAAAACAACATAACAGCATTCCTTTGGAAACAAAAAAGAATCTATTCGGGGGGG

AAATGCGAGATATTTTGTTCCACCATAGAAAATTTTTGGATTCGTCCCTTATCAAAGAAT
GA

>rps11
ATGGCAAAAGCTATACCGAGACGTAGAAATGGACGTATTGGTTTACGTAAGAGTACAC
GTAGAATACCAAAGGGAGTTATTCACGTTCAAGCAACTTTTAATAACACCATTGTTACT
GTTACAGATATACGGGGTAGGGTGGTTTCTTGGGCCTCTGCTGGTACTTGTGGATTCAA
GGGTACGAGAAGGGGGACGCCATTTGCTGCTCAAATGGCAGCAGGAAATGCTATTCGT
ACAGTAGTAGATCAAGGTATGCAACGAGCAGAAGTCATGATAAAAGGCCCTGGTCTCG
GAAGAGACGCAGCATTACGAGCTATTCGTAGAAGTGGTATACTATTAACTTTTGTACGG
GATGTCACTCCTATGCCACATAATGGCTGTAGACCTCCGAAAAAAAGACGTGTGTAG

>rps12_copy2
ATGCCAACGATTAAACAACTTATTAGAAATACAAGACAGCCAATCAGAAATGTCACCA
AATCACCCGCGCTTGGGGGATGCCCCCAGCGCCGAGGAACATGTACTAGGGTGTATAT
CACCCCCAAAAAACCAAACTCTGCCTTACGTAAAGTTGCCAGAGTACGATTAACCTCT
GGATTTGAGATCACTGCTTATATACCCGGTATTGGCCATAATTTACAAGAACATTCTGTA
GTCTTAGTAAGAGGGGGAAGGGTTAAGGATTTACCCGGTGTGAGATATCACATTGTTCG
AGGAACCCTAGATGCTGTCGGAGTAAAGGATCGTCAACAAGGGCGTTCTAAATATGGG
GTAAAAAAGCCAAAATAA

>rps12
ATGCCAACGATTAAACAACTTATTAGAAATACAAGACAGCCAATCAGAAATGTCACCA
AATCACCCGCGCTTGGGGGATGCCCCCAGCGCCGAGGAACATGTACTAGGGTGTATAT
CACCCCCAAAAAACCAAACTCTGCCTTACGTAAAGTTGCCAGAGTACGATTAACCTCT
GGATTTGAGATCACTGCTTATATACCCGGTATTGGCCATAATTTACAAGAACATTCTGTA
GTCTTAGTAAGAGGGGGAAGGGTTAAGGATTTACCCGGTGTGAGATATCACATTGTTCG
AGGAACCCTAGATGCTGTCGGAGTAAAGGATCGTCAACAAGGGCGTTCTAAATATGGG
GTAAAAAAGCCAAAATAA

>rps14
ATGGCAAGGAAAGGTTTGATTCAGCGGGAGAAGAAAAGGAAAAATTTGGAACAGAA
ATATCATTTGATTCGCCGATCCTCAAAAAAAGAAATAAGCAAAGTTCCCTCATTGAGTG
ACAAATGGGAAATTTATGGAAAGCTACAATCCCCACCGCGGAATAGTGCACCGAACCG
CCTTCATCGACGTTGTTTTTCAACTGGAAGATCGAGAGCTAATTATCGAGACTTTGGAC
TATCCGGGCACATACTTCGTGAAATGGTTTATGCATGTTTGTTGCCGGGAGCAACAAGA
TCAAGTTGGTAA

>rps15
ATGGTAAAAAATTCATTCATTTCAATTATTTCTCAAGAAGAAAACAAAGGGTCAGTTGA
ATTTCAAGTATTCAGTTTTACCAATAAGATACGAAAACTTACTTCTCATTTAGAATTACA
CAAAAAGGACTATTTATCTCAGAAGGGTTTGCGGAAAATTTTGGGAAAACGTCAACGA
CTACTGGTTTATTTGTCAAAAAAAAAATAGAGTACGTTATAAAGAATTAATTGATCAGTTG
GATATTCGAGAAATAAAAACTCGTTAA

>rps16
ATGGTAAAACTTCGTTTGAAACGATGTGGTAGAAAGCAACGAGCCGTCTATCGAATCG
TTGCAATTGATGTTCGATCCCGACGAGAAGGAAAAAATCTTCAGAAACTGGGTTTTTAT
GATCCGATAAAGAATGAAACTTATTTAAACGTTCCTTCTATTCTATACTTCCTTGAAAGG
GGTGCTCAACCTACAGGAACTGTTCGGGATATTTTAAAGAAGAAGGGGCTTTTTAAGG
AACTTCGCCTTAATTAA

>rps18
ATGGATAAATCCAAGCGACCTTTTCTTAATAAATCCAAGAGAACTTTTCGTAAGCGTTT
GCCCCCGATTGAATCGGGGGATCGAATTGATTATAGAAACATGAGTTTAATTAGTCGATT
TATTAGTGAACAAGGAAAAATATTATCTAGACGGGTGAATAGATTAACCTTGAAACAAC
AACGATTAATTACTACTGCCATAAAACAAGCTCGTATTTTATCTTTGTTACCCTTTCTCA
ATAATGAGAAACAATTTGAAAGAACCGAGTCGGCCCCCAGAACTACTGGTCTTAGAAC
TAGTAATAACTAG

>rps19
GTGACACGTTCACTAAAAAAAAAATCCTTTTGTAGCCAATCATTTATTACAAAAAATTGA
AAAGCTTAATACAAAAGCAGAAAAAAAAAATAATAGTAACTTGGTCCCGGGCATCTACC
ATTATACCCACAATGATCGGCCATACGATTGCTATCCATAATGGGAAAGAGCATTTGCCT
GTTTATATAACAGATCGTATGGTAGGACACAAATTGGGAGAATTCGCGCCTACTTTAAAT
TTCCGAGGACATGCCAAAAGCGATAATAGATCTCGTCGTTAA

>rps2
ATGACAAGAAGATATTGGAACATCTGTTTGGAAGAGATGATGGAGGCAGGAGTTCATT
TTGGCCATGGTACTAGGAAATGGAATCCAAAAATGGCACCTTATATTTCTGCAAAGCGT
AAAGGTATTCATATTATAAATCTTACTAAAACTGCCCGTTTTTTATCAGAAGCTTGTGATT
TGATTTTTGATGCGGCAAGTAGGGGAAAACAATTCTTAATTGTTGGTACCAAAAATAAA
GCAGCTGATTCAGTAGCGTGGGCTGCAATAAGGGCCCGGTGTCATTATGTTAATAAAAA
ATGGCTTGGCGGTATGTTAACGAATTGGTCCACTACTGAAACCAGGCTTTATAAGTTCC
GGGACTTAAGAATGGAACAAAAAACAGGGAAATTCAATCGTCTTCCGAAAAGAGATG
AAGCTATGCTGAAAAGACAATTATCTCGCTTGCAAACATATCTGGGCGGAATTAAATAT
ATGACAGGGTTACCCGATATTGTAATCATCGTCGATCAGCACGAAGAATATACGGCCTT
GCGAGAGTGTATCACTTTGGGAATTCCAACAATTTGTTTAATCGATACAAATTGTGACC
CCGATATCGCAGATATTTCGATTCCAGCAAATGATGACGCTATATCTTCAATCCGATTAAT
TCTTAACAAATTAGTATTCGCAATTTGTGAAGGGCGTTCTAGCTATATAAGAAATCCTTG
A

>rps3
ATGGGACAAAAAATAAATCCACTTGGTTTCAGACTTGGTACAACTCAAGGTCATCACT
CCCTTTGGTTTGCCCAACCAAAAAAGTATTCTGAGGGTTTACAAGAAGATCAAAAAAT
AAGAAATTGTATCAAGATATATATACAAAAAAATATGAGAATACCCTCTGGAGTCGAGG
GAATTGCACGTATAGAGATTCAAAAAAGAATCGATCTGATACAGGTCATAATCTTTATG
GGATTTCCAAAGTTATTAATAGAAAGTCGTCCGCGGGGAATCGAAGAATTACAAACGA

ATTTACAAAAAGAATTTAATTGTGTAAACATAAACCGAAAACTAAATATTGCTATCACA
AAAATTGCAAAACCTTATGGAAACCCTAATATTCTTGCAGAATTTATAGCCGGACAATT
AAAGAATAGAGTTTCATTTAGAAAAGCAATGAAAAAAGCTATTGAATTAACTGAACAA
GCAGATACAAAAGGAATTCAAGTGCAAATTGCAGGGCGTATCGACGGAAAAGAAATT
GCACGTGTTGAATGGATCAGAGAGGGTAGGGTTCCCCTACAAACCATCCGAGCTAAAA
TTGATTATTGTTCCTATACAGTTCAAACAATCCATGGGGTATTAGGCATCAAAATTTGGA
TATTTATAGACGAGGAATGA

>rps4
ATGTCACGTTATCGAGGACCTCGTTTCAAAAAAATACGCCGGCTGGGGGTTTTACCAG
GATTAACTAATAAAAGGCCTAGAGCCGGAAGTGATCTTAGAAACCAATCGCGTTCCGG
GAAAAAATCTCAATATCGTATTCGTCTAGAAGAAAAACAAAAATTGCGTTTTCATTATG
GTCTTACAGAACGACAATTACTTAAATATGTTCGTATCGCCAGAAAAGCCAAGGGCTCA
ACAGGTCAAGTTTTACTACAATTACTTGAAATGCGTTTGGATAACATTCTTTTTCGATTG
GGTATGGCTTCGACTATTCCCGCAGCCCGTCAATTAGTTAACCATAGACATATTTTAGTT
AATGGTCGTATAGTAGATATACCAAGTTATCGCTGCAAACCCCAAGATATGATTACAGCA
AAGGACGAACAAAAATCCAGAACTCTGATTCAAAATTCTCTCCATTCATCTCCTACTGA
GGGATTGCCAAACCATTTGACCCTTCACCCATTCGAATATAAAGGATTAGTCAATCAAA
TAATAGATAGTAAATGGGTCGCTTTGAAAATAAATGAATTGCTAGTCGTAGAATATTATT
CTCGTCAGACTTAA

>rps7_copy2
ATGTCACGCCGAGGTACTGCAGAAAAAAAAACAGCAAAATCCGATCCAATTTATCGTA
ATCGATTAGTTAACATGTTGGTTAACCGTATTATGAAACACGGAAAAAAATCATTGGCT
TATCAAATTATCTATCGAGCCGTGAAAAAGATTCAACAAAAGACAGAAACAAATCCAC
TATCCGTTTTACGTCAAGCAATACGTGGAGTAACTCCCGATATAACAGTAAAAGCAAGA
CGTGTAGGTGGATCGACTCATCAAGTTCCCATTGAAATAGGATCCACGCAAGGAAAGG
CACTTGCCATTCGTTGGTTATTAGCGGCATCCCGAAAACGTCCGGGTCGAAATATGGCT
TTCAAATTAAGTTCCGAATTAGTGGATGCTGCCAAAGGGAGTGGCGATGCCATACGCA
AAAAGGAAGAGACTCATAGAATGGCAGAGGCAAATAGAGCTTTTGCACATTTTCGTTA
A

>rps7
ATGTCACGCCGAGGTACTGCAGAAAAAAAAACAGCAAAATCCGATCCAATTTATCGTA
ATCGATTAGTTAACATGTTGGTTAACCGTATTATGAAACACGGAAAAAAATCATTGGCT
TATCAAATTATCTATCGAGCCGTGAAAAAGATTCAACAAAAGACAGAAACAAATCCAC
TATCCGTTTTACGTCAAGCAATACGTGGAGTAACTCCCGATATAACAGTAAAAGCAAGA
CGTGTAGGTGGATCGACTCATCAAGTTCCCATTGAAATAGGATCCACGCAAGGAAAGG
CACTTGCCATTCGTTGGTTATTAGCGGCATCCCGAAAACGTCCGGGTCGAAATATGGCT
TTCAAATTAAGTTCCGAATTAGTGGATGCTGCCAAAGGGAGTGGCGATGCCATACGCA
AAAAGGAAGAGACTCATAGAATGGCAGAGGCAAATAGAGCTTTTGCACATTTTCGTTA
A

>rps8

ATGGGTAGGGACACTATTGCTGAGATAATAACCTCTATACGAAATACCGATATGGATAGA
AAAAGAGTGGTTCGAATAGGATCTACTAATATTACCGAAAGTCTTGTTAAAATCCTTTTA
CGAGAGGGATTTATCGAAAACGTGAGAAAACATCGCGAAAACAACAAACCTTTTTTG
GTTTTAACCTTACGACATAGAAGGAATAGAAAAAGACCCTATAATAGAAATATTTTAAA
TTTAAAACGGATCAGTCGACCTGGTCTACGAATCTATTCTAACTATCAACGAATTCCTAG
AATTTTAGGTGGGATGGGAATTGTAATTCTTTCTACTTCTCGAGGTATAATGACAGACCG
AGAAGCTCGAGAAGCAAAAATCGGCGGAGAAATTTTGTGTTATATATGGTAA

>ycf1_copy2
ATGATTTTTCAATCTTTTCTACTAGGTAATCTAGTATCCTTATGCATGAAGATAATCAATT
CGGTCGTTGTGGTCGGACTCTATTATGGATTTCTGACCACATTCTCCATAGGGCCCTCTT
ATCTCTTCCTTCTCCGAGCTCAGGTTATGGAAGAAGGAACCGAGAAGAAGGTATCAGC
AACCACTGGTTTTATTACGGGACAGCTCATGATGTTCATATCTATCTATTATGCGCCAATG
CATCCAGCATTGGGTAGACCTCATACAATAACTGTCCTAGCTCTACCATATCTTTTGTTT
CATTTCTTCTGGAACAATCACAAACACTTTGGATCTACTACCAGAAATTCAATGCGCAA
TCTCAGCATTCAATGTGTATTTCTGAATAATCTCATTTTTCAATTATTCCACCATTTCATTT
TACCAAGTTCAATGTTAGCCAGATTAGTCAACATTTATCTGTTTCGATGCAACAATAAGA
TGTTATTTTTAACAAGTAGTTTTCTTGGTTGGTTAATTGGTCACATTTTATTCATGAAATG
GCTTGGATTGGTATTAGTCTGGATACGGCAAAATAATTCTATTAGATCGAATGTACTTATT
CGATCTAATAAGTACCTTGTGTCAGAATTGACAAATTCTATGGCTCGGATCTTTAGTATT
CTCTTATTTATTACCTGTGTCTACTATTTAGGCAGAATACCGTCACCCATTCTGACTAAG
AAACTGACAGAAACCTCAAAAACGGAAGAAAGGGTGGAAAGTGAGGAAGAAAGAG
ATGTAGAAATAGAAACCGCTTCCGAAATGAGGGGGGTGAAACAGGAACAAGAGGGAT
CCACCGAAGAAGATCCTTCCCCTTTTTCGGAAGAAAGGGAGGAAAAAATCGATGAAA
CGGAAGAAATCCGAGTGAATGGAAAGGAAAAAACAAAGGATGAATTCCACTGTCACT
TTACAGTGACAGGCTATAACAATAGCGAAGATTCAGATAAGCATTTTCATAGAAATCCA
GAAAATTCAAAATTTAAAATACTTGATCAAAAAGATTTAGCTGAGAATAAAGACTTCTG
GTGGTTTTGGTTTGAAAAACCTCTTGTAACTCTTCTTTTCGATTATAACCGATGGAATCG
ACCGTTTCGATACATAAAAAATAATCGATTTGAAAGGGCTGTAAGAAATCAAATGTCAC
AATACTTTTTTGACACATGCAAAAGTGATGGAAAACAAAGAATATCTTTTACATACCCG
CCAAGTTTGTCAACTTTTTGGGAAATTATAAAAAAAAAAATACCTTTTTATCAGCTAGA
CAATCGTTGGATTTTTATCAACAAACAAAAAAGTAATAATCTAAACAACGAATTTAGAA
ATAGAATTGAAGCTCTAGACAACAAGTCTTTTTTTATGGATAGAATCCAAACAAGAACT
AGGTTGTGTAATGACGATAAAGAATATTTGCCTATAATGTATGATCCTTTCTTGAACGGG
CCATATCGCGGAACAATTAAAAAAAGGTTTTCACTTTCAATCATAACTTCTATAGAAAAT
TTCAAAAAGAGAGTTGGAATAAATAGGATTCATAGTATTCTTCTTCCGTATACAGATTCC
CAAGAATTAATCAATGAAATTATAACTGATGCTAATGATCAAAAAATTATAAAAAAAGA
TATTGGAATAAAAGAAATCAGTAAAACCCCCCCCCGCTGGTCATACAAATTAATCACCG
AATTGGAACAACAATCGGGAGAAGATCAAGAAGACGTGCCCTTGGATCATCAAATTCG
ATCAAGAAAAGCTAAGCGTGTGGTCATTTTTACTGATAACAAGCAAGATACTAATTCTA
AGAATACCGAAACTTCGGATCAAACAGACGAACTAGCTTTGATACGTTATTCACAACA
ATCTGATTTTCGACGAGGCATAATCAAAGGTTCTATACGTGCTCAAAGGCGTAAAATAG
TAATTTGGGAGTTGTTTCAAGCAAATGTGCACTCCCCGCTTTTTTTGGACAGGATCAAA
TCGCCCCCTTTTTCTTTTGATATCTTTGAACTAACGAAACCCCAATTTAGAAATTGGATA

AAGGGCATAGGAGAGGTCAAAATGGGGGAGTATGCAGCAGAACAGACAAAAAGAGA
AGAGAAGAAAAGAAAAGAAATAGTACAGATAGAAATAGCAGAAGCCTGGGATAACAT
TCCCTTTGCACAAGGAATAAGAGGTTACATGTTAATAACTCAATCAATTCTTAGAAAAT
ATTTTCTATTGCCTTCGTTGATAATAGCAAAAAATATTGGACGTATGTTATTATTTCAACT
TCCGGAATGGTTTGAGGATTTACAGGAATGGAATAGAGAAATGCATGTTAAATGTACCT
ATAATGGTGTTCAATTATCAGAAACAGAATTTCCGCAAAATTGGGTAACAGACGGTATT
CAGATTAAAATTCTATTTCCTTTCCATCTAAAACCTTGGCAGAGATCTAACTCTCCTAGA
GATCTAATGAAAAAAAAAAAGACAAAATCTGATTTTTGTTTTTTGACAGTTTGGGGAAT
GGAAGCTGAACTTCCTTTTGGTTCTCCTAGAAAGCGCCCCTCATTTTTTGAACCCATTA
TTAAAGAGCTCAATAAAAAAAATGGAAAAATTTAAAAAGAAATATTTTCTTGCTCTAAAG
ATTTTAAAAGGAAAAACAAAATTACTTATAAAAATTTTAAAAGAAATAAAAAAAATGGAT
TATGAAAAATGTTTTATTTATAAAAAAAACGAATAAAAGAACTTAATACAATTCTATTATT
TAGGTTTAGATTAAGAGAAGTATATGAATCGAATGAAACTAAAAAAGAAAAAGATTTTC
TGATCAGAAATCAAATAATTGATGAATCATTTAATCAGATTCAATCTCCGGCTTGGTCAA
AATCTTCACTGACAAAAAAAAAAATGAAAGATCTGACTAATAGAATCAATATAATTCGA
AATCAAATAGAAAGAATTACAAAAGAGAAAAAAAAAACTCCAGCAATAAATATTAGTC
TTAACAAAATAAGTGTTAATCCTAAAAAATTAGAGTCACCAAAAAAAATTTGGGCAAATA
TTAAAAAGAATAAATGCTCGATTAACCTATAAATTTTATTTTATAAAAATTGTCATTGAA
AAAATATACATAGATATTTGTTTATCTATCATTAATATTCCCCTAATCAATACACAACTTTT
TCTTGAATCAACAAAAAAAATTATTGATCAATACATTTACAATAATGAACCAAATCAAG
AAAAAGTAAATAAAAAAAATCCAAATACAAGTAGTTTTATTTCGACTATAAAAAAGTCA
CTTGATATTGTTAGTAACAAAAATTCACATATTTTTAGTGATTTATCCTGCTTGTCACAAG
CATATGTATTTTATAGGTTATCTCAAGCCCAAGTTAGAAGTTTGTATAAATTACAATCTGT
TCTTCAATATCAGGGAACATCTTTATTTCTTAAGACTGAAATAAAGGATTTTTTTGGAAC
ACAAGGAATATTTCAGACCGAATTAGGGCATAAAAAACCTAAAAATTCTGCAATGAAT
GACTGGAAAAAATGGTTAAGAGGACATTATCAATATGATTTATCTCAGATTAGATGGTCT
AGATTAATACCACAAAGATGGCGGAATAATCGAATTAATAAACGACGTTGTATGACTAA
AAAAAATTATAAATGGGAGTCATATGAGAAAGACAAATTCAATTTTTACAGAAAAGAA
AATGTTTCTGAAGTATATTCATTATTGAATGAAAAAGAAAATTTTTCAAAATACAATAGA
TATGACCTTTTATCATATAAATCTCTTAATTATGAAAAAAAGGCCTCTTCTATTTATTCTAT
TTATAGGTATGGATTACGATTGGAAATAAATAAGAACCAAGAGCTTTTTTACAATTCTAA
CATACATAAAGACAACTTTTTTGATATCCTGGAGAGTACTCTTATCAATAGTTATCTAGG
AAAAGGCAGTTTTTTATATATCGAAAAAAATGCAGATAGAAAATATTTTGATTGGAAAA
TCTTAAAATTTTATCTTAAAAAAAAAGCTGATCTTGAAACCTGGATACAGATCGATACC
AAGATTAACCAAAGTACTAAGACGGGTACTAATAATTACCAAATAGTGGATCAATTTGA
TAAAAAAGATCTTTTTTATCTTACGATTCATCAAGATAAAAAAAATATCAAAAGGGTATT
TTTTGATTGGATGGGAATGAATGAAGAAATACTAAACCGTCCAATACCGAATCTGGAAC
TTTGGTTATTCCCAGAATTTTTACAACTATATAATGTATATAAAATAAAACCGTGGATTAT
ACGAAGCAAATTTCTTCTTTTAAATTCGAATCAAAATAAAAATATTGGGGTAAGTACGA
ATAAAAATACCAATGAAAAACAAAAAAGGAATTTTTTGATTCGATGGTATAAAAAAATA
AAGAATAAAAATAAAGAAGAACCCGTAGGACAAGGCAATCTGGGATCTGTTCTATCAA
ACCAACAAAAGGATATTGAAGAAAATGATGCGGAATCAAACAGTAAAAAGCCTAAAA
AGAAAAAACAATACAAAAGTAAAACAGAAGCAGAAATGGATCTCTTCCTGAAACGTT
ATTTGCTTTTTCAATTGAGATGGGACGATTCTTTGAATCAAAGAATAATCAATAATATCA

AGGTATATTGTCTCCTGCTTAGACTGAATAATCCAAGAAAAATTGCTATATCCTCGATTC
AACGGGGAGAACTCTGTTTGGATATAATGTTGATTGAACACAATTTAACTTTTCCAGAA
TTGATGAAAAAGGGACTTTTTATTATCGAACCCACTCGTGTGTCTGTAAAAAATGATGG
ACAATTTATTATGTATCAAACCATGGGTATTTCCTTGGTTCATAAAAGTAAATACAAAAC
AAGTAATCAAAGATACCACGAACAAGGATATATTGATAAGACTCATTTTAATGAGTCCA
TTTCAGAATATCAAAAAAGAATCAGAAATAGAGATAAAAATGATTTTGATTTGCTTGTT
CCTGAAAATATTTTATCATCTAGACGTCGTAGAGAGTTGAGAATTCTCATTTGTTTCAAT
TCAAAAAATGGTAAGAGTGCGGATAGAAATCCAGTATGTTATAACGAGAACTGGGCAA
AAAATATTAGAAATAAAAATGAATTAATTCAATTAAAGTTTTTCTTTGGCCTAATTATCG
ATTAGAAGATTTAGCTTGTATTAATCGTTATTGGTTTAATACCAATAACGGCAGTCGTTTT
AGTATGTTAAGGATACATATGTATCCGCGGTTAAAAACTTTCATTTTTCTTTTACCATAG

>ycf1
ATGATTTTTCAATCTTTTCTACTAGGTAATCTAGTATCCTTATGCATGAAGATAATCAATT
CGGTCGTTGTGGTCGGACTCTATTATGGATTTCTGACCACATTCTCCATAGGGCCCTCTT
ATCTCTTCCTTCTCCGAGCTCAGGTTATGGAAGAAGGAACCGAGAAGAAGGTATCAGC
AACCACTGGTTTTATTACGGGACAGCTCATGATGTTCATATCTATCTATTATGCGCCAATG
CATCCAGCATTGGGTAGACCTCATACAATAACTGTCCTAGCTCTACCATATCTTTTGTTT
CATTTCTTCTGGAACAATCACAAACACTTTGGATCTACTACCAGAAATTCAATGCGCAA
TCTCAGCATTCAATGTGTATTTCTGAATAATCTCATTTTTCAATTATTCCACCATTTCATTT
TACCAAGTTCAATGTTAGCCAGATTAGTCAACATTTATCTGTTTCGATGCAACAATAAGA
TGTTATTTTTAACAAGTAGTTTTCTTGGTTGGTTAATTGGTCACATTTTATTCATGAAATG
GCTTGGATTGGTATTAGTCTGGATACGGCAAAATAATTCTATTAGATCGAATGTACTTATT
CGATCTAATAAGTACCTTGTGTCAGAATTGACAAATTCTATGGCTCGGATCTTTAGTATT
CTCTTATTTATTACCTGTGTCTACTATTTAGGCAGAATACCGTCACCCATTCTGACTAAG
AAACTGACAGAAACCTCAAAAACGGAAGAAAGGGTGGAAAGTGAGGAAGAAAGAG
ATGTAGAAATAGAAACCGCTTCCGAAATGAGGGGGGTGAAACAGGAACAAGAGGGAT
CCACCGAAGAAGATCCTTCCCCTTTTTCGGAAGAAAGGGAGGAAAAAATCGATGAAA
CGGAAGAAATCCGAGTGAATGGAAAGGAAAAAACAAAGGATGAATTCCACTGTCACT
TTACAGTGACAGGCTATAACAATAGCGAAGATTCAGATAAGCATTTTCATAGAAATCCA
GAAAATTCAAAATTTAAAATACTTGATCAAAAAGATTTAGCTGAGAATAAAGACTTCAC
TAAATACTAA

>ycf2_copy2
ATGAATTCCATTGGACCCAGAAATGATACATTGGAAGAATCCATTGGGTCTTCCAATATC
AATAGGTTGATTCTTTCGCTCCTGCATCTTCCAAAAGGGAAAAAGATCTCTGGGAGTTG
TTTCCTGAATCCGAAAGAGAGTACTTGGGTTCTCCCAATAACTAAAAAGTGTAGCATGC
CTGAATCTAACAGGGGTTCGCGGTGGTGGAGGAACTGCTGGATCGGAAAAAGGAGGG
ATTCTAGTTGTAAGATATCTAATGAAACCGTCGCTGGAATTGAGATCTTATTCAAAGAG
AAAGATATCAAATATCTGGAGTTTCTTTTTGTATATTATATGGAAAATCCGATCCGCAAG
GACCATGATTGGGAATTGTTTGATCGTCTTTCTCTGAGGAAGAGGCGAAATCGAATCA
ACTTGAATTCGGGACCGCTATTCGAAATCTTAGTGAAACAATGGATTTCTTATCTCATGT
CTGCTTTTCGTGAAAAAATACCCATTATTGAAGTGGAGGGTTTCTTCAAACAACAAGG
GGCTGGGTCAACTATTCAATCAAATGATATTGAGCGTGTTTCCCATCTCTTCTCGAGAA

ACAAGTGGGCTTTTTCTTTGCAAAATTGTGCTCAATTTCAGATGTGGCAATTCCGCCAA
GATTTCTTCGTTAGTTGGGGAAAGAATCCGCACGAATCGGATTTTGTGTCGAGAGAGG
ATTTGATTTGGTTAGACAATGCGTGGTTGGTAACCAAGGATCCGTTTTTTAGCAAGGTA
CGGAATGTATCGTCAAATATTCAATATGATTCCAATTCCACAAGATCTAGTAGTTTCGTT
CAAGTAACGGATTCTAGCCAACTGAAAGGATCTTCTGATCAATCCAGAGATCGTTTGG
ATTCCATTAGTGAGGATTCGGAATATCACACATTGATCAATCAAAGAGAGATTCAACAA
CTAAAAGAAAGATCGATTCTTTGGGATCCTTCCTTTCTTCAAACGGAACGAACAGAGA
TAGAATCAGACCGATTCCCGAAATGCCTTTCTGGATATTCCTCAATGTCCCGGCTATTCA
CGGAACGTGAGAAGCAGATGATTAATCATCTGCTTCCGGAAGAAATCGAAGAATTTCT
TGGGAATCCTACAAGATCCGTTCGTTCTTTTTTCTCTGATAGATGGTCAGAACTTCATCT
GGGTTCGAATCCTACTGAGAGGTCCACTAGAGATCAGAAATTGCTGAAGAAACAACA
AGATCTTTCTTTTGTCCCTTCCAGGCGATCGGAAAATAAAGAAATGGTTCCTATTTTCA
AGATAATTACATATTTACAAAATACCGTCTCAATTCATCCTATTTCATCAGATCCGGGATG
TGATATGGTTCCGAAGGATGAACCGGATATGGACAGTTCCAATAAGATTTCATTCTTGA
ACAAAAATACATTTTTTGATTTATTTCATCTATTCCATGACCGGAACAGAGGAGGGTAC
GCGTTACACCACGATTTTGAATCAGAAGAGAGATTTCAAGAAATGGCAGATCTATTCAC
TCTATCAATAACCGAGCCGGATCTGGTGTATCATAAGGGATTTGCCTTTTCTATTGATTC
CTACGGATTGGATCAAAAACAATTCTTGAATGAGGTATTCAACTCCAGGGATGAATCGA
AAAAGAAATCTTTATTGGTTCTACCTCCTATTTTTTATGAAGAGAATGAATCTTTTTCTC
GAAGGATCAGAAAAAAATGGGTCCGCCGGATCTCCTGCGGGAATGATTTGGAAGATCC
AAAACCCAAAATAGTGGTATTTGCTAGCAACAACATAATGGAGGCAGTCAATCAATATA
GATTGATCCGAAATCTGATTCAAATCCAATATAGTACCTATGGGTACATAAGAAATGTAT
TGAATCGATTCTTTTTAATGAATAGATCCGATCACAACTTCGAATATGGAATTCAAAGGG
ATCAAATAGGAAAGGATACTCTGAATCATAGAACTCTAATAAAATATACGATCAACCAA
CATTTCTCGAATTTTAAAAAGAGTCGGAAGAAATGGTTTGATCCTCTTATTTGGATTTCT
CGAACCGAGAGATCCATGAATCGGGATCCTGATGCATATAGATACAAATGGTCCAATGG
GAGTCAGAATTTCCAGGAACATTTTGTTTCTGAGCAGAAGAACCGTTTTCAAGTAGTG
TTCGATCGATTACGTATTAATCAATATTCGATTGATTGGTCTGAGGTTATCGACAAAAAA
GATTTGTCTAAGCCACTTCGTTTCTTTTTGTCCAAGTCACTTCTTTTTTTGTCCAAGTTG
CTTTTCTTTTTGTCTAACTCACTTCCTTTTTTCTGTGTGAGTTTCGGGAATATCCCCATGC
ATAGGTCCGAGATCCACATCTATGAGAAAGGTCCGAATGATCAACTCTGCAATCAGTTG
TTAGAATCAATAAGTCTTCTAATTGTTCATTTGAACAAATGGAAACCCTTTTTATTGGAT
GATCATGATACTTCTCAAAAATCGAAATTCTTGATCAATGGAGGAACAATATCACCATTT
TTGTTCAATAAGATACCAAAGTGGATGATTGACTCATTCCATACTCGAAATAATCGGAA
ATCCTTTGATAACACGGATTCCTATTTCTCAATGATATTCCACGATCAAGACAATTGGCT
GAATCCCGTGAAACCATTTCATAGAAGTTCATTGATATCTTCTTTTTATAAAGCAAATCG
ACTTCGATTCTTGAATAATCCACATTACTTCTGCTTCTATTATAACACAAGATTCCCCTTT
TATTATGTGGAAAAGGACCGTATCAATAATTCTGATTTTACGTATGGACAATTCCTCAAT
ATCTTGTTCATTCGCAAGAAAATATTTTCTTTGTGCGTCGGTAAAAAAAAACATGCTTT
TGGGAGGAGAGATACTATTTCACCAATCGAGTCACAGGTATCTAACATATTCATACCTAA
TGATTTTCCACAAAGTGGTGACGAAGCGTATAACTTGTACAAATCTTTCCATTTTCCAA
GTCGATCCGATCCATTCGTTCATAGAGCTATTTACTCGATCGCAGACATTTCCGGAATAC
AGGGACAAATAGTCCATTTTGAAAGAACTTATTGTCAACCTCTTTCAGATATGAATCTAT
CTGATTCAGAAGGGAAGAACTTGCATCAGTATCTCAATTTCAATTCAAACGTGGGTTTG

ATTCAGACTCCATGTTCTGAGAAATATTTACCATCCGAAAAGAGGAAAAAACGGAGTC
TTTGTCTAAAGAAATGCGTTAAGAAAGGGCAGATGTATAGAACCTTTCAACGAGATAG
TGCTTTTTCAACTCTCTCCAAATGGAATCTATTCCAAACATATATGCCGTGGTTCCTTAC
TTCGACAGGGTACAAATATCTAAATTTTCTATTTTTAGATACTTTTTCAGACCTATTGCCG
ATACTAAGTAGCAGTCAAAAATGGGTATCCATTTTTCATGATATTATGCATGGATCAGGT
ATATCATGGCGAATTCTTCAGAAAAATTTGCGTCTTCCACAATGGAATCCGATAAGTGA
GATTTCGAGTAAGTGTTTACATAATCTTCTTCTGTCCGAAGAAATGATTCATCGAAAGA
ATGAGTCACCATTGATATCGACACATCTGAGATCGCCAACGCCAAAGGTTCGGGAGTT
CCTCTATTCAATCCTTTTCCTTCTTCTTGTTGCTGGATATCTTCTCGTTCGTACACACCTT
CTCTTTGTTTCCGGGGCCTATAGTGAGTTACAGACAGAGTTCGAAAAGGTCAAATCTTT
GATGATTCCATCATCTATGATTGAGTTGCGAAAACTTCTGGATAGGTATCCTACAGAACC
GAATTCTTTCTGGTTAAAGAATCTCTTTCTAGTTGCTCTGGAACAATTAGGATATTCTCT
AGAAGAAATACGGGATTCTGATTCTCGCGGCAACGTGCTTGGTCCCACTTATGGGGTC
AAATCAATACGTTCTAAGAATAAATATTTGAATATCAATCTCATTGATCTCATACCAAATC
CCATTAATCGAATCACTTTTTCGAGAAATACGAGACATCTAAGTCATACAAGTAAAGAG
ATCTATTCATTGATAAGAAAAAGAAAAGACGTGAACGGGGATTGGATTGATGATAAAAT
AGAATCTTGGGTCGCGAACAGTGATTCGATTGATGATGAAGAAAGAGAATTCTTGGTT
CAGTTCTCCACCTTAACGACAGAAAAAAGGATTGATCAAATTCTATTGAGTCTGACTCA
TAGTGATCCTTTATCAAAGAATGACTCTGGTTATCAAATGATTGAACAACCGGGAGCAA
TTTACTTACGATACTTAGTTGACATTCATAAAACGTATCTAATGAATTATGAGTTCAATAC
ATCCTGTTTAGCAGAAAGACGGATATTCCTTGCTCATTATCAGACAATCACTTATTCACA
AACTTCGTGTGGGGCTAATAGTTTTCATTTCCCATCTCATGGAAAACCCTTTTCGCTCCG
CTTAGCCTTATCCCCCTCTAGGGGTATTTTAGTGATAGGTTCTATAGGAACTGGACGATC
CTATTTGGTCAAATACCTAGCGACAAACTCCTATGTTCCTTTCATTACGGTATTTCTGAA
CAAGTTCCTGGATAACAAGCCTAAAGGTTTTCTTATTGATGATATCGATATTGATGCTAG
TGACGATATCGATGCTAGTGACGATATCGATGCTAGTGACGATATCGATCGTGACCTTGA
TACAGAGCTGGAACTGCTAACTAGGATGAATGCGCTAACTATGGATATGATGCCGGAAA
TAGACCTATTTTATATCACCCTTCAATTCGAATTAGCAAAAGCAATGTCTCCTTGCATAA
TATGGATTCCAAACATTCATGATCTGGGTGTGAATGAGTCGAATTACTTATCCCTCGGTC
TATTAGTGAACCATCTATCCAGGGATTGTCAAAGATGTTCCACTAGAAATATTCTTGTTA
TTGCTTCGACTCATATTCCCCCAAAAGTGGATCCCGCTCTAATAGCTCCGAATAAATTAA
ATACGTGCATTAAGGTACGAAGGCTTCTTATTCCACAACAACGAAAGCACTTTTTCACT
CTTTCATATACTAGGGGATTTCACTTGGAAAAGAAAATGTTCCATACTAACGGATTCGG
GTCCATAACCATGGGTTCCAATGCACGAAATCTTGTAGCACTTACCAATGAGGCCCTAT
CGATTAGTATTACACAGAAGAAATCAATTATAGACACTAATACAATTAGATCCGCTCTTC
ATAGACAAACTTGGGATTTGCGATCCCAGGTAAGATCGGTTCAGGATCATGGGACCCTT
TTCTATCAGATAGGAAGGGCTGTAGCGCAAAATGTACTTCTAAGTAATTGTCCCATAGAT
CCTATATCTATCTATATGAAGAAGAAATCATGTAATGAAGGGGATTCTTATTTGTACAAAT
GGTACTTCGAACTTGGAACGAGCATGAAGAAATTAACGATACTTCTTTATCTTTTGAGT
TGTTCTGCCGGATCGGTCGCTCAAGATCTTTGGTCTCTACCCGCCGGACCCGATGAAAA
AAATGGAATCACTTCTTATTATGGACTTGTTGAGAATGATTCTGATCTAGTTCATGGCCT
ATTAGAAGTAGAAGCCGCTCTGGTGGGATCTTCATGGACAGAAAAAGATTGCAGTCAG
TTTGATAATGATCGAGTGACATTGCTTCTTCGGCCCGAACCGAGGAATCCCTTAGATGT
GATGCAAAACGGATCTTGTTCTATCCTTGATCAGAGATTTCTCTATGAAAAATACGAATC

GGAGTTTGAAGAAGGGGAGGGAGAAGGAGCCCTTGACCCGCAACAGATAGAGGAGG
ATTTATTCAATCACATAGTTTGGGCTCCTAGAATATGGCGCCCTTGGGGCTTTCTATTTG
ATTGTATCGAAAGGCCCAATGAATTGGGATTTCCCTATCAGTCCAGGTCATTTCGAGGC
AAGCGGATCATTTATGATGAAGAGGGTGCGCTTCAAGAGAATGATTCGGCGGAGTTCT
TGCAGATAACCACGCAGTACCAGACACGAGATAGATCTTCCAAAGAACAAGGCTTTTT
TCGAATAAGCCAATTCATTTGGGACGCAGATCCACTCTTTTTCCTATTCAAAGATCAGC
CCCCTGGCTCTGTGTTTTCACATCGAGAATTAGTTGCAGATGAAGAGATGTCAAAGGG
GCTTCTTACTGTCCAAACAGATCCTCCTACATCTATATATAAACGCTGGTTTATCAAGAA
TACGCAGGAAAAGCACTTCGAATTGTTGATTAATCGTCAGAGATGGCTTAGAACCAATA
GTTCATTATCTAATGGATCTTTCCGTTCTAATACTCTATCCGAGAGTTATCAGTATTTATC
AAACCTGTTCCTATCTAACGGAACGCTATTGGATCAAATGACAAAGACATTGTTGAGAA
AGAGATGGCTTTTCCCGGATGAAATGAAAATGGGATTCATGTAA

>ycf2

ATGAATTCCATTGGACCCAGAAATGATACATTGGAAGAATCCATTGGGTCTTCCAATATC
AATAGGTTGATTCTTTCGCTCCTGCATCTTCCAAAAGGGAAAAAGATCTCTGGGAGTTG
TTTCCTGAATCCGAAAGAGAGTACTTGGGTTCTCCCAATAACTAAAAAGTGTAGCATGC
CTGAATCTAACAGGGGTTCGCGGTGGTGGAGGAACTGCTGGATCGGAAAAAGGAGGG
ATTCTAGTTGTAAGATATCTAATGAAACCGTCGCTGGAATTGAGATCTTATTCAAAGAG
AAAGATATCAAATATCTGGAGTTTCTTTTTGTATATTATATGGAAAATCCGATCCGCAAG
GACCATGATTGGGAATTGTTTGATCGTCTTTCTCTGAGGAAGAGGCGAAATCGAATCA
ACTTGAATTCGGGACCGCTATTCGAAATCTTAGTGAAACAATGGATTTCTTATCTCATGT
CTGCTTTTCGTGAAAAAATACCCATTATTGAAGTGGAGGGTTTCTTCAAACAACAAGG
GGCTGGGTCAACTATTCAATCAAATGATATTGAGCGTGTTTCCCATCTCTTCTCGAGAA
ACAAGTGGGCTTTTTCTTTGCAAAATTGTGCTCAATTTCAGATGTGGCAATTCCGCCAA
GATTTCTTCGTTAGTTGGGGAAAGAATCCGCACGAATCGGATTTTGTGTCGAGAGAGG
ATTTGATTTGGTTAGACAATGCGTGGTTGGTAACCAAGGATCCGTTTTTTAGCAAGGTA
CGGAATGTATCGTCAAATATTCAATATGATTCCAATTCCACAAGATCTAGTAGTTTCGTT
CAAGTAACGGATTCTAGCCAACTGAAAGGATCTTCTGATCAATCCAGAGATCGTTTGG
ATTCCATTAGTGAGGATTCGGAATATCACACATTGATCAATCAAAGAGAGATTCAACAA
CTAAAAGAAAGATCGATTCTTTGGGATCCTTCCTTTCTTCAAACGGAACGAACAGAGA
TAGAATCAGACCGATTCCCGAAATGCCTTTCTGGATATTCCTCAATGTCCCGGCTATTCA
CGGAACGTGAGAAGCAGATGATTAATCATCTGCTTCCGGAAGAAATCGAAGAATTTCT
TGGGAATCCTACAAGATCCGTTCGTTCTTTTTTCTCTGATAGATGGTCAGAACTTCATCT
GGGTTCGAATCCTACTGAGAGGTCCACTAGAGATCAGAAATTGCTGAAGAAACAACA
AGATCTTTCTTTTGTCCCTTCCAGGCGATCGGAAAATAAAGAAATGGTTCCTATTTTCA
AGATAATTACATATTTACAAAATACCGTCTCAATTCATCCTATTTCATCAGATCCGGGATG
TGATATGGTTCCGAAGGATGAACCGGATATGGACAGTTCCAATAAGATTTCATTCTTGA
ACAAAAATACATTTTTTGATTTATTTCATCTATTCCATGACCGGAACAGAGGAGGGTAC
GCGTTACACCACGATTTTGAATCAGAAGAGAGATTTCAAGAAATGGCAGATCTATTCAC
TCTATCAATAACCGAGCCGGATCTGGTGTATCATAAGGGATTTGCCTTTTCTATTGATTC
CTACGGATTGGATCAAAAACAATTCTTGAATGAGGTATTCAACTCCAGGGATGAATCGA
AAAAGAAATCTTTATTGGTTCTACCTCCTATTTTTTATGAAGAGAATGAATCTTTTTCTC
GAAGGATCAGAAAAAAATGGGTCCGCCGGATCTCCTGCGGGAATGATTTGGAAGATCC

AAAACCCAAAATAGTGGTATTTGCTAGCAACAACATAATGGAGGCAGTCAATCAATATA
GATTGATCCGAAATCTGATTCAAATCCAATATAGTACCTATGGGTACATAAGAAATGTAT
TGAATCGATTCTTTTTAATGAATAGATCCGATCACAACTTCGAATATGGAATTCAAAGGG
ATCAAATAGGAAAGGATACTCTGAATCATAGAACTCTAATAAAATATACGATCAACCAA
CATTTCTCGAATTTTAAAAAGAGTCGGAAGAAATGGTTTGATCCTCTTATTTGGATTTCT
CGAACCGAGAGATCCATGAATCGGGATCCTGATGCATATAGATACAAATGGTCCAATGG
GAGTCAGAATTTCCAGGAACATTTTGTTTCTGAGCAGAAGAACCGTTTTCAAGTAGTG
TTCGATCGATTACGTATTAATCAATATTCGATTGATTGGTCTGAGGTTATCGACAAAAAA
GATTTGTCTAAGCCACTTCGTTTCTTTTTGTCCAAGTCACTTCTTTTTTTGTCCAAGTTG
CTTTTCTTTTTGTCTAACTCACTTCCTTTTTTCTGTGTGAGTTTCGGGAATATCCCCATGC
ATAGGTCCGAGATCCACATCTATGAGAAAGGTCCGAATGATCAACTCTGCAATCAGTTG
TTAGAATCAATAAGTCTTCTAATTGTTCATTTGAACAAATGGAAACCCTTTTTATTGGAT
GATCATGATACTTCTCAAAAATCGAAATTCTTGATCAATGGAGGAACAATATCACCATTT
TTGTTCAATAAGATACCAAAGTGGATGATTGACTCATTCCATACTCGAAATAATCGGAA
ATCCTTTGATAACACGGATTCCTATTTCTCAATGATATTCCACGATCAAGACAATTGGCT
GAATCCCGTGAAACCATTTCATAGAAGTTCATTGATATCTTCTTTTTATAAAGCAAATCG
ACTTCGATTCTTGAATAATCCACATTACTTCTGCTTCTATTATAACACAAGATTCCCCTTT
TATTATGTGGAAAAGGACCGTATCAATAATTCTGATTTTACGTATGGACAATTCCTCAAT
ATCTTGTTCATTCGCAAGAAAATATTTTCTTTGTGCGTCGGTAAAAAAAAACATGCTTT
TGGGAGGAGAGATACTATTTCACCAATCGAGTCACAGGTATCTAACATATTCATACCTAA
TGATTTTCCACAAAGTGGTGACGAAGCGTATAACTTGTACAAATCTTTCCATTTTCCAA
GTCGATCCGATCCATTCGTTCATAGAGCTATTTACTCGATCGCAGACATTTCCGGAATAC
AGGGACAAATAGTCCATTTTGAAAGAACTTATTGTCAACCTCTTTCAGATATGAATCTAT
CTGATTCAGAAGGGAAGAACTTGCATCAGTATCTCAATTTCAATTCAAACGTGGGTTTG
ATTCAGACTCCATGTTCTGAGAAATATTTACCATCCGAAAAGAGGAAAAAACGGAGTC
TTTGTCTAAAGAAATGCGTTAAGAAAGGGCAGATGTATAGAACCTTTCAACGAGATAG
TGCTTTTTCAACTCTCTCCAAATGGAATCTATTCCAAACATATATGCCGTGGTTCCTTAC
TTCGACAGGGTACAAATATCTAAATTTTCTATTTTTAGATACTTTTTCAGACCTATTGCCG
ATACTAAGTAGCAGTCAAAAATGGGTATCCATTTTTCATGATATTATGCATGGATCAGGT
ATATCATGGCGAATTCTTCAGAAAAATTTGCGTCTTCCACAATGGAATCCGATAAGTGA
GATTTCGAGTAAGTGTTTACATAATCTTCTTCTGTCCGAAGAAATGATTCATCGAAAGA
ATGAGTCACCATTGATATCGACACATCTGAGATCGCCAACGCCAAAGGTTCGGGAGTT
CCTCTATTCAATCCTTTTCCTTCTTCTTGTTGCTGGATATCTTCTCGTTCGTACACACCTT
CTCTTTGTTTCCGGGGCCTATAGTGAGTTACAGACAGAGTTCGAAAAGGTCAAATCTTT
GATGATTCCATCATCTATGATTGAGTTGCGAAAACTTCTGGATAGGTATCCTACAGAACC
GAATTCTTTCTGGTTAAAGAATCTCTTTCTAGTTGCTCTGGAACAATTAGGATATTCTCT
AGAAGAAATACGGGATTCTGATTCTCGCGGCAACGTGCTTGGTCCCACTTATGGGGTC
AAATCAATACGTTCTAAGAATAAATATTTGAATATCAATCTCATTGATCTCATACCAAATC
CCATTAATCGAATCACTTTTTCGAGAAATACGAGACATCTAAGTCATACAAGTAAAGAG
ATCTATTCATTGATAAGAAAAAGAAAAGACGTGAACGGGGATTGGATTGATGATAAAAT
AGAATCTTGGGTCGCGAACAGTGATTCGATTGATGATGAAGAAAGAGAATTCTTGGTT
CAGTTCTCCACCTTAACGACAGAAAAAAGGATTGATCAAATTCTATTGAGTCTGACTCA
TAGTGATCCTTTATCAAAGAATGACTCTGGTTATCAAATGATTGAACAACCGGGAGCAA
TTTACTTACGATACTTAGTTGACATTCATAAAACGTATCTAATGAATTATGAGTTCAATAC

ATCCTGTTTAGCAGAAAGACGGATATTCCTTGCTCATTATCAGACAATCACTTATTCACA
AACTTCGTGTGGGGCTAATAGTTTTCATTTCCCATCTCATGGAAAACCCTTTTCGCTCCG
CTTAGCCTTATCCCCCTCTAGGGGTATTTTAGTGATAGGTTCTATAGGAACTGGACGATC
CTATTTGGTCAAATACCTAGCGACAAACTCCTATGTTCCTTTCATTACGGTATTTCTGAA
CAAGTTCCTGGATAACAAGCCTAAAGGTTTTCTTATTGATGATATCGATATTGATGCTAG
TGACGATATCGATGCTAGTGACGATATCGATGCTAGTGACGATATCGATCGTGACCTTGA
TACAGAGCTGGAACTGCTAACTAGGATGAATGCGCTAACTATGGATATGATGCCGGAAA
TAGACCTATTTTATATCACCCTTCAATTCGAATTAGCAAAAGCAATGTCTCCTTGCATAA
TATGGATTCCAAACATTCATGATCTGGGTGTGAATGAGTCGAATTACTTATCCCTCGGTC
TATTAGTGAACCATCTATCCAGGGATTGTCAAAGATGTTCCACTAGAAATATTCTTGTTA
TTGCTTCGACTCATATTCCCCCAAAAGTGGATCCCGCTCTAATAGCTCCGAATAAATTAA
ATACGTGCATTAAGGTACGAAGGCTTCTTATTCCACAACAACGAAAGCACTTTTTCACT
CTTTCATATACTAGGGGATTTCACTTGGAAAAGAAAATGTTCCATACTAACGGATTCGG
GTCCATAACCATGGGTTCCAATGCACGAAATCTTGTAGCACTTACCAATGAGGCCCTAT
CGATTAGTATTACACAGAAGAAATCAATTATAGACACTAATACAATTAGATCCGCTCTTC
ATAGACAAACTTGGGATTTGCGATCCCAGGTAAGATCGGTTCAGGATCATGGGACCCTT
TTCTATCAGATAGGAAGGGCTGTAGCGCAAAATGTACTTCTAAGTAATTGTCCCATAGAT
CCTATATCTATCTATATGAAGAAGAAATCATGTAATGAAGGGGATTCTTATTTGTACAAAT
GGTACTTCGAACTTGGAACGAGCATGAAGAAATTAACGATACTTCTTTATCTTTTGAGT
TGTTCTGCCGGATCGGTCGCTCAAGATCTTTGGTCTCTACCCGCCGGACCCGATGAAAA
AAATGGAATCACTTCTTATTATGGACTTGTTGAGAATGATTCTGATCTAGTTCATGGCCT
ATTAGAAGTAGAAGCCGCTCTGGTGGGATCTTCATGGACAGAAAAAGATTGCAGTCAG
TTTGATAATGATCGAGTGACATTGCTTCTTCGGCCCGAACCGAGGAATCCCTTAGATGT
GATGCAAAACGGATCTTGTTCTATCCTTGATCAGAGATTTCTCTATGAAAAATACGAATC
GGAGTTTGAAGAAGGGGAGGGAGAAGGAGCCCTTGACCCGCAACAGATAGAGGAGG
ATTTATTCAATCACATAGTTTGGGCTCCTAGAATATGGCGCCCTTGGGGCTTTCTATTTG
ATTGTATCGAAAGGCCCAATGAATTGGGATTTCCCTATCAGTCCAGGTCATTTCGAGGC
AAGCGGATCATTTATGATGAAGAGGGTGCGCTTCAAGAGAATGATTCGGCGGAGTTCT
TGCAGATAACCACGCAGTACCAGACACGAGATAGATCTTCCAAAGAACAAGGCTTTTT
TCGAATAAGCCAATTCATTTGGGACGCAGATCCACTCTTTTTCCTATTCAAAGATCAGC
CCCCTGGCTCTGTGTTTTCACATCGAGAATTAGTTGCAGATGAAGAGATGTCAAAGGG
GCTTCTTACTGTCCAAACAGATCCTCCTACATCTATATATAAACGCTGGTTTATCAAGAA
TACGCAGGAAAAGCACTTCGAATTGTTGATTAATCGTCAGAGATGGCTTAGAACCAATA
GTTCATTATCTAATGGATCTTTCCGTTCTAATACTCTATCCGAGAGTTATCAGTATTTATC
AAACCTGTTCCTATCTAACGGAACGCTATTGGATCAAATGACAAAGACATTGTTGAGAA
AGAGATGGCTTTTCCCGGATGAAATGAAAATGGGATTCATGTAA

>ycf3
ATGCCTAGATCGCGGATAAATGGAAATTTTATTGATAAGACCTTTTCAATTGTAGCCAAT
ATCTTATTACGAATAATTCCGACAACTTCAGGAGAAAAAGAGGCATTTACCTATTACAG
AGATGGTATGTCAGCTCAATCCGAAGGAAATTATGCGGAAGCTTTACAGAATTATTATG
AAGCTATGCGACTAGAGATTGATCCCTATGATCGAAGTTATATACTCTATAATATAGGTCT
TATCCACACAAGTAATGGAGAACATACGAAAGCTTTAGAATATTATTTTCGAGCACTAG
AACGAAACCCATTCTTACCACAAGCTATTAATAATATGGCCGTGATCTGTCATTACCGAG

GAGAACAGGCCATTCGGCAGGGAGATTCTGAAATTGCAGAGGCTTGGTTTGATCAAGC
CGCTGAGTATTGGAAACAAGCTATAGCGCTTACCCCAGGTAATTATATTGAAGCACATA
ATTGGTTGAAGATCACGAGACGTTTTGAATAA

>ycf4
ATGAGTTGGCGATCAGAACATATATGGATAGAACTTATAAGGGGATCTCGAAAAATAAG
TAATTTCTGCTGGGCCTTTATCCTTTTTTTAGGTTCGTTAGGATTCTTATTGGTTGGAATT
TCCAGTTATCTTGGTAAAAATTTGATATCTTTTTTTCCGTCCCAGCAAATCATTTTTTTCC
CACAAGGAATCGTGATGTCTTTCTACGGAATTGCGGGTCTCTTTATTAGTTCCTATTTGT
GGTGCACAATTTCCTGGAATGTAGGTAGTGGTTATGATCGATTCGATAAAAAGGAAGGA
ATAGTATGTATTTTTCGTTGGGGATTTCCTGGAAAAAATCGTCGCATATTCCTCCAATTC
CTTATAAAAGATATTCAGTCCGTTAGAATAAGAATAGAAGTTAAAGAGGGTATTTATGCT
CGCCGTGTCCTTTATATGGACATAAGAGGCCAAGGGGCCATTCCCTTGGCCCGTACTGA
TGAGAATTTGACTCCACGAGAAATTGAACAAAAAGCTGCCGAATTGGCCTATTTCTTG
CGTGTACCAATTGAAGTATTTTGA
